# Craniofacial development: genetics, signaling pathways, teratogenic mechanisms, and clinal significance

**DOI:** 10.1186/s43556-026-00574-6

**Published:** 2026-09-24

**Authors:** Xinli Lv, Juan Du

**Affiliations:** 1 Laboratory of Orofacial Development, Laboratory of Molecular Signaling and Stem Cells Therapy, Capital Medical University School of Stomatology, Fanjiacun Road No.9, Beijing, 100070 China; 2 Department of Geriatric Dentistry, Capital Medical University School of Stomatology, Fanjiacun Road No.9, Beijing, 100070 China

**Keywords:** Craniofacial development, Craniofacial disorders, Genetic regulators, Signaling pathway, Teratogenic mechanisms

## Abstract

Craniofacial development is a highly coordinated and stage-specific process involving neural crest cell migration, proliferation, differentiation, and epithelial-mesenchymal transition. Genetic mutations, signaling pathway abnormalities, and exposure to environmental teratogens can disrupt normal development. These anomalies can lead to cleft palate, craniosynostosis, and other common congenital craniofacial malformations. Although significant progress has been made in understanding craniofacial development in recent years, the systematic integration of these factors remains limited. Here, we provide a comprehensive review of the cellular and molecular mechanisms underlying normal craniofacial development, focusing on the coordinated regulation of cranial neural crest cells by transcription factors, signaling pathways and epigenetic programs. We further summarize major pathogenic mechanisms implicated in craniofacial disorders. Additionally, the teratogenic mechanisms underlying maternal endogenous and external environmental exposures are explored, with particular emphasis on their interactions with genetic factors in reshaping developmental trajectories. We emphasize the central role of gene-environmental interactions in craniofacial pathogenesis by integrating evidence from developmental biology, multi-omics studies and clinical studies. Subsequently, we outline current clinical diagnostic techniques and management strategies for craniofacial malformations, pointing out the existing challenges and future directions. This review establishes a comprehensive framework linking gene regulation, metabolic environment, and clinical implications. It is expected to facilitate deeper insights into disease mechanisms and promoting the translation of basic research findings into clinical diagnosis, prevention, and management of craniofacial disorders.

## Introduction

Craniofacial development commences during early embryonic stages, representing a complex, meticulously orchestrated, and evolutionarily conserved morphogenetic event. Its formation originates from a unique population of pluripotent stem cells known as neural crest (NC) [1]. The coordinated growth of craniofacial components relies on the migration, proliferation, and differentiation of neural crest cells (NCCs). Cranial neural crest cells (CNCCs) start at the edge of the neural plate, go through epithelial-mesenchymal transition (EMT), move to the facial prominences and pharyngeal arches, and then make craniofacial bone, cartilage, connective tissue, teeth, and other neural structures around them [2, 3]. Craniofacial development is highly controlled. Genetic, environmental, or metabolic disruptions that lead to imbalances at any point can cause congenital anomalies, including cleft lip and palate, craniosynostosis, and jaw dysplasia [4]. The occurrence of craniofacial deformities can have adverse effects on patients' breathing, eating, speaking, and mental health, and it also imposes a heavy economic burden on families and society [5]. Therefore, figuring out how molecules control craniofacial development and looking into all the things that can cause craniofacial deformities are still important scientific questions in developmental biology and oral medicine. Such research has the potential to enhance clinical prevention and treatment of craniofacial deformities, consequently decreasing the prevalence of congenital craniofacial anomalies.

Research on craniofacial development has evolved from descriptive embryology to the genetic and molecular mechanism levels [1]. With the advancement of multi-omics and high-throughput technologies, numerous key genes, signaling pathways, and pathogenic mutations associated with craniofacial development have been identified, significantly advancing our understanding of its genetic basis [6]. Moreover, classical signaling pathways including fibroblast growth factor (FGF), bone morphogenetic protein (BMP), wingless-type (WNT), and sonic hedgehog (SHH) have been shown to play major roles in NCCs fate determination, epithelial-mesenchymal interactions and bone tissue formation [7]. However, malformations are often associated with environmental factors and variations in a single gene or signaling pathway which cannot always fully explain their occurrence [8]. This suggests that normal craniofacial development relies on the complex interplay of a genetic-molecular regulatory network composed of key genes, regulatory factors, signaling pathways, and many environmental factors [8]. However, existing literature predominantly focuses on exploring individual genes, pathways, or specific disease types. While such studies are rapidly accumulating, they often lack clear theoretical frameworks and comprehensive systematic reviews. Therefore, it is essential to summarize current research, construct a multi-level, multidimensional framework integrating developmental biology, molecular genetics, and clinical pathological phenotypes, and further explore its clinical translational significance.

Based on the above background, this review focuses on the genetic and molecular mechanisms that jointly drive craniofacial morphogenesis, including key pathogenic genes and complex regulatory factors, key signaling pathways and their interactions, important environmental teratogenic factors and their genetic mechanisms. We finally discussed the pathological significance, clinical application value and potential intervention strategies of craniofacial malformations, so as to lay a foundation for the formulation of prevention-oriented and precise treatment strategies.

## Genetic and epigenetic regulation of craniofacial development

**Fig. 1:**
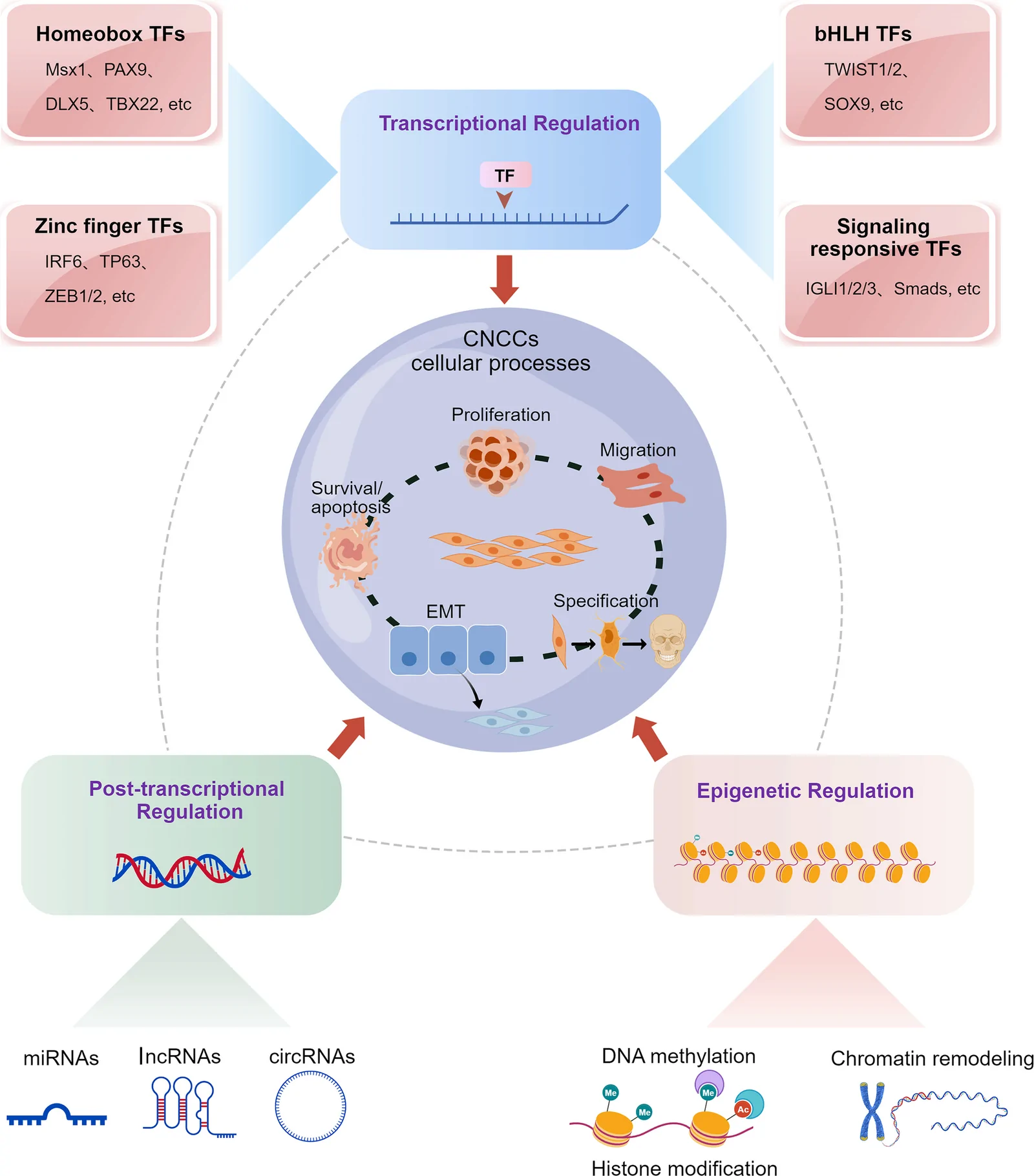
Integrated transcriptional, post-transcriptional, and epigenetic regulatory networks governing cranial neural crest cell function. Craniofacial development is governed by a hierarchical and highly coordinated system of gene regulatory mechanisms. At the transcriptional level, evolutionarily conserved transcription factor families exert precise spatiotemporal control over lineage commitment and craniofacial patterning through dynamic, context-dependent gene expression programs. Post-transcriptionally, non-coding RNAs, including microRNAs, long non-coding RNAs, and circular RNAs modulate mRNA stability, translational efficiency, subcellular localization, and decay kinetics, thereby adding a critical layer of regulatory precision. Concurrently, epigenetic mechanisms, including DNA methylation, covalent histone modifications, and ATP-dependent chromatin remodeling complexes, dynamically regulate chromatin architecture, enhancer promoter interactions, and transcriptional competence across developmental time windows. Critically, these three regulatory tiers do not operate in isolation. Rather, they engage in extensive crosstalk and functional interdependence, converging on core CNCCs cellular processes including proliferation, migration, EMT, specification and survival, to ensure robust execution of morphogenetic processes, which are essential for proper craniofacial morphogenesis. RNAs, Ribonucleic acids; microRNAs, Micro ribonucleic acids; mRNA, Messenger ribonucleic acid; DNA, Deoxyribonucleic acid; ATP, Adenosine triphosphate; CNCCs, Cranial neural crest cells; EMT, Epithelial-mesenchymal transition

The genetic regulation of craniofacial development should be regarded as a multi-level, dynamically integrated molecular regulatory system (Fig. 1). Core transcription factors, non-coding RNAs, and epigenetic modifications collectively act upon key developmental genes [9, 10]. By precisely controlling the timing, intensity, and spatial distribution of gene expression, they ensure the orderly development of CNCCs and craniofacial structures [11]. Unraveling these regulatory layers and their interactions provides the logical starting point for understanding craniofacial development mechanisms and linking subsequent signaling pathways to discussions on teratogenesis.

### Transcriptional regulation

The core transcriptional regulators are the central hub of the gene regulatory network of craniofacial development. It can regulate the survival, migration, differentiation and other key processes of CNCCs by directly regulating target gene transcription or mediating chromatin remodeling [12]. A research team has established a high-resolution epigenetic map of embryonic craniofacial development. This map has found that there are a wide range of bivalent promoter characteristics in key transcription factors, indicating that the craniofacial gene regulatory network is in a rapid and staged activation state during morphogenesis [13]. Given that craniofacial development is highly spatiotemporally specific, different developmental stages rely on the coordinated regulation of specific transcription factors to precisely orchestrate key biological processes such as NC formation, migration, and craniofacial morphogenesis. Therefore, this section will base on the developmental stages to systematically describe the regulatory roles of relevant transcription factors in craniofacial development. The representative transcription factors discussed in this section and their teratogenic phenotypes are summarized in Table 1.

**Table 1:** Key transcriptional regulators involved in craniofacial development and their associated teratogenic phenotypes

Transcription factor	Major biological functions	Craniofacial phenotypes	Teratogenic mechanisms	References
TFAP2	Define the neural plate border and participate in early NC lineage induction, regulate ectodermal differentiation and craniofacial skeletal development	Branchio-oculo-facial syndrome, Char syndrome, and various craniofacial skeletal malformations	Impaired NCCs induction and survival leading to abnormal differentiation of NC derived tissues	[14–16]
Pax	Regulate proliferation, migration, and lineage specification of CNCCs and involved in craniofacial skeletal and tooth development	Cleft palate, tooth agenesis or hypoplasia, craniofacial skeletal abnormalities	Loss of transcriptional activation disrupts NC derived lineage development and craniofacial patterning	[17–21]
Sox	Maintain NCCs multipotency and stemness, regulate migration and chondrogenic lineage differentiation	Cartilage developmental defects and severe craniofacial malformations	Impaired maintenance of NCCs multipotency and disrupted chondrogenic differentiation	[22–24]
FoxD3	Maintains NCCs multipotency and regulates stem cell differentiation	NC developmental defects and associated craniofacial abnormalities	Dysregulation of WNT-dependent transcriptional programs affecting NCCs fate determination and differentiation	[25, 26]
Snail/Slug	Defective mesenchymal transition, impaired NCCs migration and cranial suture development	NC defects, cleft palate	Failure of EMT and NCCs migration due to persistent epithelial characteristics	[27–30]
TWIST	EMT regulation, CNCCs migration, craniofacial skeletal development, Craniofacial morphogenesis and palatal fusion	Saethre-Chotzen syndrome, craniosynostosis, craniofacial dysplasia, Ablepharon-Macrostomia syndrome, Barber-Say syndrome	Defective mesenchymal transition, impaired NCCs migration, cranial suture development, disrupted mesenchymal differentiation and palatal development	[31–39]
ZEB	EMT regulation, palatal elevation, NC development, cell migration	Ablepharon-Macrostomia syndrome, Barber-Say syndrome, cleft palate	Disrupted mesenchymal differentiation and palatal development, impaired epithelial plasticity and palatal shelf elevation	[40–44]
Dlx	Regulate proximodistal patterning of pharyngeal arch structures and craniofacial skeletal morphogenesis	Pharyngeal arch patterning defects, mandibular malformations, and forebrain developmental abnormalities	Disruption of proximodistal patterning in pharyngeal arches affecting craniofacial morphogenesis	[45, 46]
Msx	Regulate epithelial-mesenchymal interactions and participate in the development of the skull, jaw, and teeth	Cleft lip and palate, tooth agenesis or abnormal tooth morphology, cranial skeletal defects	Act as downstream transcriptional regulators of the BMP signaling pathway controlling cell differentiation and craniofacial morphogenesis	[47–49]
Irf6	Regulate epithelial differentiation and palatal epithelial fusion and involved in neural tube and craniofacial development	Van der Woude syndrome, cleft lip and/or palate, mandibular malformations, and neural tube defects	IRF6 expression is regulated by TGF-β/SMAD signaling and interacts with TFAP2A and GRHL3 to coordinate epithelial differentiation and tissue fusion	[50–53]

*TFAP2* Transcription factor AP2, *Pax* Paired Box, *CNCCs* Cranial neural crest cells, *Sox* SRY-Box, *FoxD3* Forkhead box D3, *WNT* Wingless-Type MMTV Integration Site Family, *NC* neural crest, *NCCs* neural crest cells, *EMT* Epithelial-Mesenchymal Transition, *ZEB* Zinc-finger E-box-binding homeobox factors, *Dlx* Distal-less Homebox, *Msx* Msh homeobox, *BMP* Bone Morphogenetic Protein, *Irf6* Interferon regulatory factor 6, *TGF-β* Transforming growth factor-β, *GRHL3* Grainyhead like transcription factor 3

#### Key transcription factors of NCCs specification

During vertebrate embryogenesis, pluripotent cell states emerge transiently prior to lineage commitment and morphogenetic patterning. NCCs, which play a central role in craniofacial development, exemplify this plasticity through their capacity to differentiate into diverse ectodermal and mesodermal derivatives [54]. The signaling networks governing pluripotency maintenance and lineage specification are highly conserved across vertebrates; accordingly, the transcription factors VENT homeobox/Nanog Homeobox (VENTX/NANOG) and octamer-binding transcription factor 4 (OCT4), identified as vertebrate-specific developmental potential guardians (vsDPGs), have been functionally characterized as key regulators of cellular potency [55]. During neurulation, vsDPGs confer dual developmental competence upon ectodermal cells at the neuroepithelial border (NEB): they sustain pluripotency while simultaneously enabling acquisition of extraembryonic mesenchymal identity via an endogenous reprogramming mechanism, thereby specifying the NCC lineage. Furthermore, vsDPGs are expressed in bipotent neural mesodermal progenitors (NMPs), a population essential for posterior body axis elongation and formation. Besides, Transcription factor AP2 (TFAP2) and Pax belong to the neural plate boundary-specific factors, which are mainly involved in the definition of the neural plate boundary region and the early induction of the NC lineage [56, 57]. The homeobox transcription factors (TFs) are key factors in the regulation of the first branchial arch (BA1) pattern formation [58]. TFAP2α and TFAPβ are highly expressed in early NCCs, and are involved in cell differentiation and survival in the facial ectoderm and induce bone formation [14].Two craniofacial malformations associated with TFAP2 abnormalities, human branchiofacial syndrome and Char syndrome, show defects in NCC derivatives in the face, heart and skin [15, 16].

The Paired Box (Pax) family transcription factors are important regulators involved in NC induction, craniofacial patterning, and lineage differentiation during embryonic development [59]. A variety of Pax proteins, such as Pax3,7,9, are considered to be able to rely on their transcriptional activation characteristics to fine-tune cell proliferation, migration and differentiation in a variety of NC derived lineages [60]. Among them, Pax3 and Pax7 are primarily expressed at the neural plate border during early embryogenesis [17]. During craniofacial development, Pax3 is involved in the migration and specification of embryonic muscle progenitor cells, while Pax7 keeps the balance between proliferation and differentiation of myogenic precursor cells [18]. They also interact with myogenic regulatory factors to influence the formation of the facial skeletal muscles of the jaw [18]. Studies have shown that Pax3 deficiency impairs neural tube closure and NC development, leading to severe developmental defects and embryonic lethality [19]. In addition, Pax9 has been shown to be mainly expressed in craniofacial bones and teeth, and it can regulate the proliferation of palatal mesenchymal cells and participates in the morphogenesis of the palate [20]. Its functional loss can lead to craniofacial malformations such as cleft palate formation, skeletal abnormalities, tooth loss or hypoplasia [21].

The SRY-Box (Sox) protein family is an important regulatory factor in the transcription factor family that responds to NC induction signals and is expressed at the boundary of the neural plate [61]. Studies have shown that Sox10 can be directly activated by TFAP2 to promote the emergence and evolution of NCCs [22]. Sox2, Sox3, Sox8, Sox9, Sox11 and Sox15 are expressed in the boundary of neural plate, which can participate in the formation of NC and non-neuroectoderm [23]. In addition, Sox transcription factors are important for controlling the developmental potential of NC progenitor cells and their subsequent lineage determination. SoxC, SoxG, SoxD and SoxE family members are expressed in the NC before migration, while Sox8, Sox9 and Sox10 are required for normal migration [23]. It is worth noting that there is functional redundancy between different Sox factors. When individual factors mutate, other factors will compensate to maintain regulatory functions. In addition, Sox9 and Sox10 play a key role in maintaining the stemness of NC and promoting the differentiation of cartilage lineage. When the gene is deleted, cartilage formation will be inhibited, resulting in severe craniofacial deformity [24].

Another important regulator of NCCs migration and pluripotency maintenance is FoxD3, the forkhead box D3 [25]. Studies have shown that the transcriptional activation and function of FoxD3 affect stem cell differentiation and are regulated by the intracellular WNT signaling pathway [26] This suggests that there are also mutual feedback and dynamic coupling between transcriptional regulators and classical developmental signaling pathways, forming a complex regulatory network to fine-tune the pluripotency and lineage differentiation of NCCs.

#### EMT associated transcriptional regulators

EMT is a well-defined cellular process in which the cells lose their epithelial features and acquire mesenchymal character with migratory potential [62]. EMT plays a critical role in both developmental and pathological processes, including embryonic morphogenesis, wound healing, and cancer progression. In recent years, as the field of EMT has attracted significant interest, particularly in the context of cancer biology, it has been proposed that the EMT processes involved in embryogenesis and cancer progression exhibit similarities in terms of phenotypic changes, molecular mechanisms, and core transcription factors [63]. Consequently, it is proposed that the initiation and development of cancer may be attributed to the abnormal activation of factors required for normal embryonic development [64]. A focus on the molecular mechanisms and regulatory networks involved in EMT during embryonic development has the potential to both advance our understanding of the complex regulatory networks governing craniofacial development and facilitate the exploration of related mechanisms in fields such as cancer research.

During the process of EMT, there is a gradual loss of epithelial markers and an increase in mesenchymal markers. A hallmark of EMT is the loss of the major epithelial marker E-cadherin, a tight junction protein encoded by the cadherin 1 (CDH1) gene [65]. Three distinct types of transcription repressors, the Snail, TWIST, and Zinc-finger E-box-binding homeobox factors (ZEB) families, directly bind to the promoter region of CDH1 and are collectively known as EMT-transcription factors (EMT-TFs) [66]. Snail and Slug are members of the Snail family of zinc-finger transcription factors [27]. They are involved in initiating EMT during the development of the NC and the mesoderm [27]. The mutation of Snail factors leads to excessive amounts of E-cadherin remaining throughout embryonic development, preventing EMT from occurring correctly and resulting in developmental abnormalities such as NC malformations and cleft palate [28, 29]. Studies have shown that snail factors are induced by the Transforming growth factor-β (TGF-β) family and both of them jointly regulate the fusion process during the development of the palate, which are closely related to the occurrence and development of cleft palate [30].

TWIST acts as a basic helix-loop-helix transcription factor in a signaling cascade that triggers mesoderm formation and is essential for normal craniofacial morphogenesis [31]. TWIST1 and TWIST2 are members of the TWIST family, share a high degree of homology, and exhibit broad and highly overlapping expression patterns during development. During craniofacial development, TWIST1 promotes mesenchymal transition and enhances the migratory capacity of CNCCs by regulating cytoskeletal remodeling, cell adhesion, and extracellular matrix-associated gene expression [32]. Moreover, TWIST1 can influence the patterning of craniofacial development by shaping the expression of other protein molecules, such as Distal-less Homebox 5 (Dlx5) and Heart and Neural Crest Derivatives Expressed 2 (Hand2) [33]. TWIST2 can interact with environmental factors such as smoking and jointly influence the normal fusion process of the palate [34]. Functional studies have demonstrated that TWIST is indispensable for proper craniofacial skeletal development and cranial suture formation. Dysregulation of TWIST expression can impair NC derived mesenchymal development and disrupt craniofacial morphogenesis [35–37]. Human studies have demonstrated that mutations in the TWIST1 gene are responsible for Saethre-Chotzen syndrome (SCS) [38], while recurrent mutations in the basic domain of TWIST2 cause Ablepharon Macrostomia and Barber-Say Syndromes [39]. They are all classified as congenital embryonic developmental diseases, distinguished by abnormal craniofacial abnormalities.

ZEB family is comprised of ZEB1 and ZEB2, encoded by Zfhx1a and Zfhx1b, respectively [67]. ZEB1 is prominently expressed throughout the mesoderm, neural tube, brain, and NC derived tissues, including the palate [40]. ZEB2 is expressed in multiple tissues, including NC, heart, palate, and limb buds [40]. Prior research indicates that the ZEB1 knockout mouse has a cleft palate due to compromised palatal elevation [41]. Additionally, the ZEB2 mutant mouse is lethal at E9.5-E10.5 due to abnormal expression of E-cadherin, resulting in developmental defects in NC formation [42]. A mutation causing ZEB2 haploinsufficiency during embryogenesis is linked to Mowat-Wilson syndrome, which is characterized by intellectual incapacity, dysmorphic facial features, microcephaly, seizures, and organ anomalies [43, 44]. Collectively, these findings demonstrate that ZEB family transcription factors are necessary for NC development, EMT and craniofacial morphogenesis. ZEB proteins contribute to the precise coordination of CNCCs development by regulating epithelial plasticity, cell adhesion and migratory capacity. Dysregulation of these proteins may ultimately lead to severe craniofacial malformations and multisystem developmental disorders.

#### Craniofacial patterning and morphogenesis regulators

During the formation of craniofacial pattern, transcription factors such as Dlx, Msh homeobox (Msx), T-Box transcription factor (TBX) and Interferon regulatory factor 6 (Irf6) are involved in the spatial organization and partition of craniofacial bones and soft tissues. The Dlx and Msx families belong to the NKL homeobox gene and play a decisive role in the facial morphogenesis of embryonic development. Dlx1 and Dlx2 are expressed in the proximal and distal ends of the first and second branchial arches, and are involved in the morphogenesis of eyes, nose, ears and teeth [45]. Changes in Dlx1 or Dlx2 influence the proximal and distal patterns of the gill arch, which causes the forebrain to grow in an abnormal way. During embryonic development, the Dlx5 gene is active in the gill arch, certain parts of the brain, long appendages, and bones. Dlx5 knockout mice die soon after birth, accompanied by vestibular organ malformations and craniofacial malformations, including anencephaly, nasal sac dysplasia and proximal mandibular arch skeletal dysplasia [46].

The Msx gene family includes Msx1-3. Msx1 and Msx2 are found in many places where epithelial and mesenchymal cells interact, and they are very active in developing cranial bones, meninges, jawbones, teeth, and other structures. The Msx homeotic domain directly interacts with the TATA-binding protein to repress transcription [47]. Additionally, heterodimers composed of Msx1 and other homodomain proteins, including Dlx2, Dlx5, Lhx2, and Pax3, demonstrate reciprocal functional antagonism in vitro [47]. Msx genes are also downstream of BMP signaling, and their expression is controlled by BMP activity gradients [48]. This means that the BMP signaling pathway and Msx genes work together to control the formation of NC. Deletion of Msx genes leads to craniofacial malformations, including cleft lip and palate, as well as dental anomalies and tooth loss [49].

Irf6, a crucial regulator of epithelial development and fusion, plays a key role in the differentiation and fusion of the palatine epithelium. Its dysfunction is closely associated with cleft lip and palate as well as mandibular deformities [50]. Irf6 has been identified to interact with multiple signaling molecules. Among these, the Smad4-Irf6 gene interaction and TGFβ-mediated Irf6 signaling cascade are essential for palatal fusion in mice [51]. Analysis of genome-wide association study data identified two functional single nucleotide polymorphisms near Irf6, which account for the majority of cleft lip and palate risk at this locus [52]. Further studies indicate Irf6 also regulates neural tube development. Overexpression of Irf6 suppresses Tfap2a and Grhl3 expression, both essential regulators for mouse neural tube formation [53], suggesting these three genes form a genetic pathway synergistically controlling neural tube formation and development.

In summary, these transcriptional regulators precisely regulate the key biological processes of CNCCs through hierarchical and networked interactions, and participate in the establishment of axial characteristics and regional specificity of craniofacial structures. The functional imbalance of these key factors not only disrupts the development process, but also may amplify the developmental disturbance effect by changing the chromatin state and transcriptional potential, and ultimately lead to a variety of craniofacial malformations.

### Post-transcriptional regulation

#### MiRNA-mediated regulation

In addition to the classical transcriptional regulatory network and epigenetic regulatory network, microRNA (miRNA) mediated post-transcriptional regulatory network is also considered to be a key level to maintain the spatiotemporal accuracy of craniofacial development [68]. miRNA is a kind of endogenous non-coding RNA with a length of about 20–24 nt. It mainly regulates its stability or translation efficiency by binding to the 3' untranslated region of the target mRNA, and inhibits gene expression at the post-transcriptional level [69]. Compared with transcription factors, the regulation of miRNA emphasizes more refinement, and its expression usually shows obvious stage changes, which is particularly important in the process of embryonic development [10, 70]. Systematic analysis of the maxillary process of E10.5-E14.5 mice showed that miRNA, as a post-transcriptional regulator, formed a highly structured regulatory network in craniofacial development, and formed a negative feedback loop with target genes through precise expression timing to maintain the balance of cell proliferation, differentiation and morphogenesis [71]. A large number of studies have shown that miRNAs play a central role in regulating key biological processes such as cell proliferation, migration, differentiation and cell fate determination, which are the basis for craniofacial development [10, 72, 73]. Impaired miRNA biosynthesis can lead to severe craniofacial malformations, suggesting that miRNA is not an auxiliary regulator, but an important part of the craniofacial development regulation system [74].

It is important to emphasize that miRNAs can only target downstream effector genes, and can also directly regulate core transcription factors and key signaling pathways. A number of studies have shown that the core components of FGF, BMP, WNT and SHH signaling pathways are important targets of miRNAs, and these signaling pathways can also reversely regulate the transcription and processing of miRNAs [75–77]. By constructing feedforward and negative feedback control loops, miRNAs play a key role in maintaining the stability of craniofacial development. In addition, in addition to microRNA-mediated regulation, emerging evidence suggests that RNA modifications, such as N6-methyladenosine, N1-methyladenosine, 5-methylcytosine modification and so on, may further regulate mRNA stability and translation processes during development [78]. However, the specific role of these RNA modifications in craniofacial morphogenesis remains to be fully elucidated.

#### Long non-coding RNAs and circular RNAs in craniofacial development

Long non-coding RNAs (lncRNAs) are a class of transcripts that are longer than 200 nucleotides in length without an open reading frame. The expression of lncRNAs is specific to tissue and developmental stage, indicating their critical roles in development, differentiation and disease [79]. Technological advances in the styudy of lncRNA structure, function, and action sites have improved our understanding of these RNAs. For instances, studies have used lncRNA microarray analysis to identify 36 lncRNAs specific to human embryonic stem cells [80]. It was found that their expression is regulated by pluripotency transcription factors, such as SOX2 and OCT4 [80]. This indicates that lncRNAs play a vital role in the regulation of embryonic development and the maintenance of stem cell characteristics. LncRNAs serve as dynamic regulators of pluripotency, tissue differentiation and germ layer specification throughout embryogenesis, where precise spatiotemporal control of lineage commitment is essential [81]. Mechanistically, lncRNAs regulate gene expression through diverse molecular mechanisms, including cis/trans regulation, chromatin epigenetic remodeling, transcriptional and post-transcriptional modulation, and signaling cascade control [82]. Through these multilayered regulatory mechanisms, lncRNAs contribute to the coordinated regulation of CNCCs proliferation, migration, differentiation and epithelial–mesenchymal interactions during craniofacial morphogenesis.

Emerging evidence further suggests that dysregulation of lncRNAs is associated with craniofacial developmental abnormalities, particularly cleft lip and palate [83]. Research has demonstrated that the lncRNA MEG3 suppresses cell growth in 2,3,7,8-Tetrachlorodibenzo-p-dioxin (TCDD) and regulates the retinoic acid (RA) and Smad pathway, consequently leading to cleft palate [84, 85]. Besides, lncPSMB1 can activate the apoptosis pathway in human embryonic palatal mesenchyme cells and is associated with the risk of NSCL/P [86]. Numerous functions and mechanisms of lncRNAs remain to be clarified and need further comprehensive investigation in the future.

Circular RNAs (circRNAs) are a category of endogenous non-coding RNAs which are characterized by a covalently closed-loop structure. The majority of circRNAs are evolutionarily conserved among species, stable, and frequently possess cell-specific and tissue-specific or developmental-stage-specific expression [87]. Certain circRNAs can modulate gene regulation by functioning as decoys or competing with miRNAs and proteins. For example, circFOXP1 could stimulate osteogenic differentiation by sponging miR-33a-5p in adipose-derived mesenchymal stem cells (ADSCs) [88]. Others establish large networks of ribonucleoprotein complexes or encode functional peptides which are translated in response to specific cellular stressors. They influence intracellular gene expression via distinct pathways, thus modulating diverse physiological processes, including embryonic development, immunological responses, and tumorigenesis [89]. The genome-wide sequencing results of a rabbit embryo indicate that circRNAs may modulate embryonic development via signaling pathways, including WNT and PI3K-AKT [90]. Through these regulatory mechanisms, circRNAs participate in the meticulous regulation of developmental signaling pathways and various essential cellular behaviors, including proliferation, senescence, apoptosis, and osteogenic differentiation of stem cells, such as embryonic stem cells, periodontal ligament stem cells, and mesenchymal stem cells [91].

Recent integrative omics analyses have identified aberrant circRNA expression profiles in tissues associated with craniofacial malformations, particularly cleft lip and palate [92]. Certain circRNAs are suggested to modulate developmental processes via circRNA-miRNA-mRNA regulatory networks, consequently affecting signaling pathways associated with cell proliferation, death, and tissue fusion [93, 94]. While the precise biological functions of many circRNAs in craniofacial development are not fully elucidated, more evidence indicates that circRNAs may be important post-transcriptional regulators contributing to developmental homeostasis.

### Epigenetic regulation

Epigenetic regulation involves mechanisms that modify gene expression through alterations in chromosomal architecture rather than DNA sequences, including DNA methylation, histone modifications, and chromatin remodeling [95]. This regulation does not directly modify gene sequences but profoundly affects transcriptional potential in particular cell types and developmental stages [96].

#### DNA methylation

Dynamic histone modifications and DNA methylation emphasize a dedicated system that maintains and regulates the epigenetic code, which was established to indicate the determinants of gene properties other than DNA sequence [97, 98]. DNA methylation establishes gene silencing and provides reinforcement by maintaining chromatin in a reasonably stable long-term repression state, whereas histone changes produce reversible local chromatin structures.

DNA methylation is one of the most extensively studied epigenetic modification and is predominantly enriched within CpG dinucleotide-rich regions of gene promoters. s. It is often linked to gene silencing and provides reinforcement by maintaining chromatin in a reasonably stable long-term repression state [97]. It is especially important for controlling gene expression during the development of an embryo [99, 100]. DNA methylation patterns can be relatively stable transmitted during mitosis, thereby preserving cell type-specific gene expression states [101]. DNA methylation plays a regulatory role in embryonic development by undergoing dynamic remodeling, which occurs in two main phases: extensive erasure of DNA methylation before embryo implantation and subsequent reconstruction of methylation patterns during early embryonic development [102, 103]. The coordinated action of DNA methyltransferases and DNA demethylases is what mostly makes this demethylation and remethylation process happen [104, 105].

Researchers have identified the expression of the crucial DNA methyltransferase enzyme DNMT1 in the epithelium and CNCCs mesenchyme throughout the entire process of palatal morphogenesis. Conditional Dnmt1 deficiency results in diminished intracellular DNA methylation, hence impeding the proliferation and differentiation of CNCCs [106]. Furthermore, a team performed a genome-wide association analysis (EWAS) on blood samples from children with cleft lip and palate to investigate the relationship between methylation at each site and cleft lip and palate subtypes [107]. Results revealed that varied methylation profiles were present in various subtypes of cleft lip and palate. These studies further demonstrated that aberrant DNA methylation within cells can cause craniofacial congenital abnormalities, particularly cleft lip and palate [107, 108].

#### Histone modification

Histone modifications are another major epigenetic regulation mechanism that dynamically affects chromatin shape and gene transcription throughout embryonic development [109, 110]. Common histone modifications encompass acetylation, methylation, phosphorylation, ubiquitination, and various newly discovered acylation-related modifications, all mediated by specific epigenetic enzymes including histone acetyltransferases, histone deacetylases, methyltransferases, and demethylases [111]. Among these modifications, histone acetylation is generally associated with transcriptional activation through relaxation of chromatin structure [112]. In addition, research indicates that histone methyltransferases and demethylases dynamically impact osteogenic differentiation in mesenchymal stem cells by epigenetically modifying lineage-specific transcriptional programs. This regulatory system, which is mediated by histone changes, is thought to be necessary for bone growth and craniofacial bone differentiation [113]. Previous study has demonstrated that histone methylation controls the generation of NC during embryonic development. For example, PRDM12 enhances H3K9me3 enrichment at the promoter regions of NC-related genes Foxd3, Slug, and Sox8, enabling the development of the anterior ectoderm and NC in Xenopus embryos [114].

In recent years, new histone changes like lactylation and butylation have also gotten a lot of attention. While the biological importance of these newly discovered histone modifications in craniofacial development is not entirely understood, increasing evidence indicates that abnormal histone modification patterns may interfere with NCCs development, epithelial–mesenchymal interactions, and craniofacial tissue differentiation, thus contributing to congenital craniofacial anomalies [115].

#### Chromatin remodeling

Chromatin remodeling is another epigenetic regulatory mechanism. Its main job is to change the position, density, and composition of nucleosomes using energy from adenosine triphosphate (ATP) [116]. Chromatin remodeling is different from the other two types because it directly changes the structure of nucleosomes. This makes it a faster and reversible way to control gene expression [117]. Chromatin remodeling frequently establishes synergistic regulatory networks with histone modifications and transcription factors [118]. Histone modifications act as signals to attract chromatin remodeling complexes, and chromatin remodeling either increases or decreases the effects of these modifications on transcription. This way, chromatin remodeling brings together regulatory information from all over the genome [119–121].

Mutations in essential enzymes or complexes associated with these three epigenetic regulatory pathways interfere with various transcription factor programs or vital signaling pathways. Mutations in Microrchidia family CW-type zinc finger 2 (MORC2), a chromatin remodeling-associated ATPase, have been linked to developmental defects involving craniofacial dysmorphism. MORC2 mutations can cause aberrant epigenetic silencing and transcriptional dysregulation, highlighting the critical role of chromatin remodeling in embryonic and craniofacial development [122]. Besides, variations in BRD4 interacting chromatin remodeling complex associated protein (BICRA), a subunit of the SWI/SNF-related non-canonical Brg1/Brm-associated factors (ncBAF) chromatin remodeling complex, have been associated to developmental defects and craniofacial dysmorphism. Functional investigations in zebrafish have shown that bicra loss causes craniofacial abnormalities, indicating that chromatin remodeling plays an important role in craniofacial development [123]. The BAF complex can also regulate the migration, proliferation, and differentiation of NCCs by modulating the expression of numerous signaling pathway genes, such as Hippo and Notch pathway [124]. There are also studies showing that Chromodomain helicase DNA-binding protein 7 (CHD7), a chromatin remodeling factor implicated in CHARGE syndrome, plays an essential role in NC development and craniofacial morphogenesis. Dysregulation of CHD7-associated enhancer activity and transcriptional networks may disrupt NC gene regulatory programs, thereby contributing to congenital craniofacial abnormalities [125].

### Genetic abnormalities in craniofacial disorders

Genetic factors underlying craniofacial malformations exhibit significant heterogeneity, arising either from syndromes caused by single-gene mutations or from the combined effects of polygenic or complex genetic structures. Current clinical and basic research indicates that diverse genetic variations, through complex gene regulatory networks, ultimately converge on processes such as the formation, migration, proliferation, and differentiation of CNCCs during craniofacial development, as well as the growth, fusion, and ossification of facial structures. Notably, distinct core events affected by gene mutations result in varied craniofacial phenotypes. Clinically, multiple congenital malformations involve craniofacial features. By cataloging these genetic alterations, we not only elucidate the molecular pathophysiology of craniofacial developmental abnormalities but also uncover crucial clues for understanding common mechanisms across different disorders. Representative genetic mutations associated with craniofacial malformations are provided in Table 2.

**Table 2:** Genetic mutations associated with craniofacial malformations

Disorders	Causative Gene(s)	Major Craniofacial Phenotypes	Key Molecular Mechanisms	References
Treacher Collins syndrome (TCS)	TCOF1, POLR1D, POLR1C, POLR1B	Mandibulofacial Dysostosis, malar hypoplasia, periorbital soft tissue anomalies, maxillomandibular hypoplasia, and ear anomalies	Impaired ribosome biogenesis, inhibited the migration of CNCCs, induced p53-mediated stress response and apoptosis	[126–130]
Nijmegen breakage syndrome (NBS)	NBS1	Craniofacial developmental abnormalities	Defective pre-rRNA transcription, nucleolar stress, impaired ribosome synthesis	[131]
Acrofacial Dysostosis Cincinnati type (AFDCIN)	Pol I-associated genes	Craniofacial dysostosis	Reduced rRNA synthesis, RPL5/RPL11-mediated MDM2 inhibition, p53 stabilization	[132, 133]
Ciliopathy-associated craniofacial anomalies	Cilia-related genes, CEP295, Sc5d, Dhcr7, Insig1/2	Facial and dental abnormalities	Defective primary cilium-mediated signaling, disrupted WNT/SHH pathways	[134–138]
Cerebrocostomandibular syndrome (CCMS)	SNRPB	Micrognathia, thoracic anomalies	Splicing factor deficiency, impaired CNCCs development	[139]
Nager and Rodriguez syndrome	SF3B4	Craniofacial and limb abnormalities	RNA splicing defects, NCCs impairment	[140]
FBRSL1-associated syndrome	FBRSL1	Craniofacial malformations, cartilage defects	Impaired CNCCs differentiation and chondrogenesis	[141]
Hemifacial microsomia (HFM)	SF3B2, FOXI3, CTDSP2	Unilateral facial hypoplasia, facial asymmetry	Abnormal p53 signaling, impaired CNCCs development and RNA splicing regulation	[142–147]
Non-syndromic cleft lip/palate (NSCL/P)	Non-coding variants, IRF6, FOXE1 enhancers, etc	Cleft lip and/or palate	Cis-regulatory element disruption, altered gene dosage	[148–150]
HOXA regulatory defects	HOXA	Craniofacial clefts, cranial base anomalies	Long-range chromatin dysregulation, altered HOXA expression	[151]
Roberts syndrome	ESCO2	Cleft lip/palate, craniofacial anomalies, limb defects	Defective chromatid cohesion, cell cycle dysregulation	[152]
Van der Woude syndrome (VWS)	IRF6	Cleft lip and/or palate, lower lip pits	Loss of transcription factor function, altered TGF-β/SMAD2/3 signaling pathway and epigenetic dysregulation	[153–156]

*TCS* Treacher Collins syndrome, *TCOF1* Treacle ribosome biogenesis factor 1, *POLR1B* RNA polymerase I subunit B, *POLR1C* RNA polymerase I and III subunit C, *POLR1D* RNA polymerase I and III subunit D, *CNCCs* Cranial neural crest cells, *p53* Tumor protein 53, *NBS* Nijmegen breakage syndrome, *pre-rRNA* Precursor ribosomal RNA, *rRNA* Ribosomal RNA, *AFDCIN* Acrofacial dysostosis Cincinnati type, *Pol I* RNA polymerase I, *RPL5* Ribosomal protein L5, *RPL11* Ribosomal protein L11, *MDM2* Mouse double minute 2 homolog, *CEP295* Centrosomal protein 295, *SC5D* Sterol-C5-desaturase, *DHCR7* 7-dehydrocholesterol reductase, *Insig1* Insulin induced gene 1, *SHH* Sonic hedgehog, *CCMS* Cerebrocostomandibular syndrome, *SNRPB* small nuclear ribonucleoprotein polypeptides B and B1, *SF3B4* Splicing factor 3b subunit 4, *FBRSL1* Fibrosin like 1, *HFM* Hemifacial macrosomia, *SF3B2* Splicing factor 3b subunit 2, *FOXI3* Forkhead box I3, *CTDSP2* CTD small phosphatase 2, *NSCL/P* Non-syndromic cleft lip with or without palate, *FOXE1* Forkhead box E1, *HOXA* Homeobox A gene cluster, *ESCO2* Establishment of sister chromatid cohesion N-acetyltransferase 2

#### Ribosome biogenesis defects

Numerous craniofacial abnormalities arise from heterozygous mutations in general regulators of essential cellular operations, including transcription and ribosome synthesis. Disruptions in ribosome synthesis result in tissue-specific congenital anomalies named ribosomopathies, which often affect craniofacial development [157]. Treacher Collins syndrome (TCS) is a serious birth defect that affects the face and skull and can be passed down in both dominant and recessive ways. The main genes involved in this disease are Treacle ribosome biogenesis factor 1 (TCOF1), RNA polymerase I subunit D (POLR1D), RNA polymerase I subunit C (POLR1C), and RNA polymerase I subunit B (POLR1B), with TCOF1 mutations being the most common [126, 127]. Loss of function or truncation of the TREACLE protein due to TCOF1 mutations, which impairs ribosomal biogenesis and CNCCs motility [128, 129]. These mutations also cause abnormal activation of p53 and its transcription targets in the early embryonic neural epithelium. The cell cycle of CNCCs is arrested, resulting in increased cell death and severe craniofacial malformations [130].

Nijmegen breakage syndrome 1 (NBS1), traditionally acknowledged as a DNA damage repair factor, has recently been shown to bind to ribosomal DNA loci within nucleoli and to interact with the RNA polymerase I transcription machinery, including TCOF1. The absence of NBS1 affects pre-rRNA transcription and promotes nucleolar stress, ultimately hindering ribosome synthesis. NBS, resulting from mutations in the NBS1 gene, is a craniofacial condition linked to compromised ribosome synthesis [131]. The NC-specific deletion of Nbs1 in mice leads to craniofacial defects throughout embryonic development, underscoring the essential role of nucleolar homeostasis and ribosome synthesis in craniofacial morphogenesis.

Ribosomal RNA (rRNA) transcription by RNA polymerase I (Pol I) is an important rate-limiting step in ribosome synthesis that is indispensable for cell survival [132]. Acrofacial Dysostosis-Cincinnati type (AFDCIN) has been linked to ribosomal dysfunction. Studies have shown that defective Pol I-dependent rRNA synthesis in NCC progenitors impairs ribosome biogenesis and causes p53 accumulation, resulting in excessive cell death and craniofacial abnormalities [133]. Mechanically, reduced rRNA transcription alters the equilibrium between rRNA and ribosomal proteins, favoring the binding of ribosomal proteins Rpl5 and Rpl11 to Mdm2 and weakening the Mdm2-p53 connection, eventually leading to p53 stabilization [132].

As a result, ribosome biogenesis defects frequently lead to nucleolar stress and p53 dependent apoptosis, reducing NCCs survival and craniofacial morphogenesis. These findings show that dysregulated ribosome biogenesis is a common pathogenic mechanism in many craniofacial ribosomopathies and related developmental abnormalities.

#### Craniofacial structure formation dysfunction

Craniofacial cells also harbor a solitary sensory organelle known as the primary cilium [134]. This organelle converts external chemical and physical stimuli into intracellular signaling cascades, mediating multiple signaling pathways. Primary ciliary dysfunction directly leads to a series of diseases and syndromes affecting multiple organs, including the face and teeth [135].

It is reported that the deletion of the CEP295 gene in the centrosome protein (CEP) family can lead to a reduction in the number of centrioles and centrosomes, trigger p53 dependent cell cycle arrest, and cause ciliary development disorders [136]. These patients may exhibit severe congenital microcephaly, facial deformities and other craniofacial symptoms [136]. In addition to ciliary structural proteins, genes associated with cholesterol metabolism can also interfere with craniofacial development by impacting primary cilia function [137, 138]. Researchers have discovered that changes in the way cholesterol is broken down within cells can lead to the abnormal development of osteoblasts [137]. Primary cilia, which are receptors that detect signals from outside the cell, control the fusion of ciliary vesicles that are not working properly and their transformation into osteoblasts by changing their number and length. Ciliary dysfunction further alters the WNT/β-catenin and hedgehog signaling pathways, consequently affecting craniofacial development [137].

In addition to the aforementioned disorders, various other genetic mutations can also affect craniofacial development. Cerebrocostomandibular syndrome (CCMS) results from heterozygous deletion of the Snrpb gene in brain and NCCs during development [139]. Pathogenic SF3B4 variants cause acrofacial disorders such as Nager-Rodriguez syndrome, also known as SF3B4-related syndromes [140]. Truncating mutations in the fibrosin-like 1 (FBRSL1) gene impair CNCCs differentiation and cartilage formation, leading to craniofacial malformations and other abnormalities [141]. Besides, Hemifacial microsomia (HFM) belongs to a congenital craniofacial disorder and is characterized by the hypoplasia of unilateral facial structures [142]. The etiology of HFM is complex and multifactorial with significant involvement of genetic factors. In recent years, genes such as splicing factor 3b subunit 2 (SF3B2) and forkhead box I3 (FOXI3) have been identified as susceptibility genes for HFM [143, 144]. Furthermore, a trio whole-exome sequencing (trio-WES) study identified a novel rare variant in CTD small phosphatase 2 (CTDSP2) [145]. Subsequent research confirmed that its deletion may abnormally activate the p53 pathway, thereby contributing to HFM pathogenesis [146]. Notably, this pathway is also implicated in the pathogenesis of multiple craniofacial syndromes, including TCS [147], suggesting common underlying mechanisms among different genetic disorders.

#### Non-coding regulatory variants and epigenetic dysfunction

Additionally, mutations in non-coding regions of genes also carry a certain genetic risk for craniofacial malformations. These variations may involve cis-regulatory elements (CREs) such as enhancers and promoters, untranslated regions (UTRs), and a series of non-coding RNAs (ncRNAs) [148]. Common variants associated with cleft lip and palate risk are preferentially enriched in enhancer regions active during craniofacial development [149]. Through high-throughput functional genomics studies, researchers further identified dozens of single nucleotide polymorphisms (SNPs) with allele-specific enhancer activity. Subsequent validation prioritized a set of functional variants, most notably those in IRF6 and FOXE1 [52, 150]. Furthermore, disruption of craniofacial-specific long-range tissue regulatory structures can also cause severe craniofacial phenotypes. For example, deletion of the global regulatory region (GCR) of the HOXA gene cluster induces craniofacial clefts and skull base abnormalities with high penetrance [151].

Clinically, numerous congenital disorders exhibit craniofacial malformations, exemplified by Roberts-SC cyst syndrome resulting from Esco2 gene mutations, characterized by growth retardation, limb atrophy, and craniofacial anomalies, including cleft lip and palate [152]. The discussion of these various conditions illustrates that many disease-causing genes ultimately affect common cellular and molecular pathways. Besides, stress responses associated with ribosomal biogenesis and preservation of transcriptional and chromatin homeostasis are also involved.

Van-der-Woude Syndrome (VWS) is one of the most common cleft lip and palate syndromes in people. Its main symptoms are cleft lip and/or palate and lower lip pits [153, 154]. We now know that mutations in the IRF6 gene, which is found in the chromosomal region 1q32.2-q32.3, cause VWS [155]. WES of a three-generation family identified a novel acquired stop codon mutation in the IRF6 gene—c.748C > T:p.R250X—which exhibited complete co-segregation with the clinical phenotype [156]. More research showed that this mutation makes it harder for IRF6 to bind to histone-modified proteins. This makes it harder for SMAD2/3 expression and phosphorylation to happen, which in turn affects the development of the face and head [156]. This means that the mutant gene plays a role in the disease's development through both epigenetic and signaling pathways.

## Signaling pathways in craniofacial development

During craniofacial development, specific morphogenetic events must be translated from key genes and transcriptional regulatory networks through intracellular and intercellular signaling pathways. Genetic and epigenetic regulators rarely act in isolation, and signaling pathways tend to act as the bridge connecting gene regulation and cellular behavior and precisely coordinate developmental processes. Extensive research has revealed that pathways including SHH, WNT, BMP/TGF-β, FGF, NOTCH/Hippo, and RA constitute the critical signaling networks governing craniofacial development [158]. These crucial pathways are laid out in Table 3. Disruption of any step in these pathways may induce craniofacial malformations at critical developmental stages. Therefore, it is necessary to systematically elucidate the functions and interactions of these signaling pathways in craniofacial development.

**Table 3:** Major signaling pathways involved in craniofacial development and associated malformations

Signaling pathway	Core components or receptors	Functions in craniofacial development	Representative regulatory features	Associated craniofacial phenotypes	References
SHH	SHH, PTCH1, SMO, GLI, primary cilia	Regulates craniofacial midline formation, facial prominence growth, and CNCCs proliferation, survival, and lineage specification	Morphogen gradient formation in the forebrain, frontonasal ectodermal zone (FEZ), and pharyngeal epithelium; lipid modification and sterol-dependent signaling	Midline defects, facial dysplasia, holoprosencephaly-like phenotypes	[159–162]
WNT	WNT ligands including WNT3, WNT9b, etc., Frizzled receptors, LRP5/6, β-catenin	Controls neural plate border specification, neural crest induction, and proliferation, regulates facial prominence growth and facial width. Regulates osteoblast differentiation	Dose-dependent signaling effects, region-specific ectodermal expression, canonical WNT/β-catenin signaling regulating cell adhesion and cytoskeletal dynamics	Non-syndromic cleft lip and facial midline defects, severe skeletal malformations, Microtia-atresia	[163–171]
BMP/TGF-β	BMP ligands, BMP4, BMP5, BMP7, TGF-β ligands, BMPRs, SMAD1/5/8, SMAD4	Regulates CNCCs survival, osteogenic and chondrogenic differentiation, craniofacial bone formation, and tooth development	Activation of canonical and non-canonical Smad signaling pathways, receptor homeostasis regulating signaling intensity	Craniofacial skeletal dysplasia, abnormal maxillomandibular development, cleft lip/palate	[172–179]
FGF	FGFs, especially FGF8, FGFR1–4, ERK1/2, PI3K–AKT, PLCγ	Promotes CNCCs survival, proliferation, migration, and osteogenic differentiation, regulates facial ectoderm–mesenchyme interactions	Receptor tyrosine kinase signaling cascades, adaptor-independent FGFR functions in cell adhesion and matrix interactions	Craniosynostosis, midfacial growth defects, skeletal abnormalities	[180–189]
NOTCH	NOTCH receptors, ligands (Jagged/Delta-like), ADAM protease, γ-secretase, CSL/RBPJ transcription complex	Controls cell fate determination, tissue patterning, and bone homeostasis during craniofacial development	Cell–cell contact-dependent activation, sequential proteolytic cleavage releasing NICD to regulate transcription	Developmental abnormalities in craniofacial tissues and skeletal structures	[190–193]
Hippo	MST1/2, SAV1, LATS1/2, YAP, TAZ, TEAD transcription factors	Regulates CNCCs proliferation, apoptosis, migration, and osteogenesis, contributes to craniofacial morphogenesis	Kinase cascade controlling nuclear–cytoplasmic localization of YAP/TAZ, crosstalk with BMP and WNT signaling	Cleft lip and palate, mandibular developmental defects, neural tube abnormalities, cranial suture dysregulation and craniosynostosis	[194–204]
Retinoic acid (RA)	Retinoic acid, RAR/RXR nuclear receptors, RA-responsive genes	Regulates cranial neural crest development and craniofacial patterning during embryogenesis	Highly dose- and time-dependent transcriptional regulation, sensitive to vitamin A metabolism, interaction with miRNA regulatory networks	Cleft palate, craniofacial dysplasia, ocular and auditory defects	[205–211]

*SHH* Sonic hedgehog, *PTCH1* Patched 1, *SMO* Smoothened, *GLI* GLI family zinc finger transcription factor, *FEZ* Frontonasal ectodermal zone, *CNCCs* Cranial neural crest cells, *WNT* Wingless-type MMTV integration site family, *WNT3* Wingless-type MMTV integration site family member 3, *WNT9B* Wingless-type MMTV integration site family member 9B, *LRP5* Low-density lipoprotein receptor-related protein 5, *LRP6* Low-density lipoprotein receptor-related protein 6, *BMP* Bone morphogenetic protein, *BMP4* Bone morphogenetic protein 4, *BMP5* Bone morphogenetic protein 5, *BMP7* Bone morphogenetic protein 7, *TGF-β* Transforming growth factor beta, *BMPRs* Bone morphogenetic protein receptors, *SMAD1* SMAD family member 1, *SMAD4* SMAD family member 4, *SMAD5* SMAD family member 5, *SMAD8* SMAD family member 8, *FGF* Fibroblast growth factor, *FGF8* Fibroblast growth factor 8, *FGFR1–4* Fibroblast growth factor receptors 1–4, *ERK1/2* Extracellular signal-regulated kinases 1 and 2, *PI3K* Phosphoinositide 3-kinase, *AKT* Protein kinase B, *PLCγ* Phospholipase C gamma, *NOTCH* Notch receptor, *ADAM* A disintegrin and metalloproteinase, *NICD* Notch intracellular domain, *CSL* CBF1/Suppressor of Hairless/LAG-1, *RBPJ* Recombination signal binding protein for immunoglobulin kappa J region, *Hippo* Hippo signaling pathway, *MST1/2* Mammalian STE20-like protein kinases 1 and 2, *SAV1* Salvador family WW domain-containing protein 1, *LATS1/2* Large tumor suppressor kinases 1 and 2, *YAP* Yes-associated protein, *TAZ* Transcriptional co-activator with PDZ-binding motif, *TEAD* TEA domain transcription factor, *RA* Retinoic acid, *RAR* Retinoic acid receptor, *RXR* Retinoid X receptor

### Major signaling pathways and the corresponding craniofacial malformations

#### SHH signaling pathway

The SHH signaling pathway constitutes the core signaling pathway regulating craniofacial midline morphogenesis and facial protuberance growth [159]. During early embryonic development, the SHH signaling pathway establishes precise concentrations of morphogenetic factors through spatially restricted expression in specific tissue centers. These sites include the forebrain, the frontonasal ectodermal zone (FEZ), and the pharyngeal epithelium [159]. This gradient serves as critical positional information, directing the proliferation, survival, directed migration, and lineage fate determination of CNCCs. Classic SHH signaling is initiated when lipid-modified SHH ligand binds to the receptor Patched (PTCH1), that activates downstream molecules such as GLI transcription factors within primary cilia [160]. These transcriptional outputs coordinate epithelial-mesenchymal interactions, and are essential for midline formation, facial protuberance growth, and osteochondral differentiation [161]. Notably, the intrinsic dependence of SHH signaling on lipid modification and sterol transport highlights the close link between morphogenesis and cellular metabolism [162]. Thus, changes in lipid homeostasis may indirectly regulate craniofacial morphogenesis through the SHH pathway.

#### WNT signaling pathway

The WNT signaling pathway is a highly conserved and important signaling pathway in living organisms. It regulates various biological processes from embryonic development to adulthood [163]. It is indicated that WNT signaling modulates neural plate boundary differentiation, CNCCs induction, and proliferation [164]. WNT signaling pathway can change facial width and protuberance growth in a dose-dependent manner [165]. During critical stages of early facial morphogenesis, multiple WNT family members exhibit region-specific expression in the ectoderm. For example, WNT3 is primarily localized in the maxillary and midnasal ectoderm whereas WNT9b shows higher expression in the midnasal, maxillary, and lateral nasal ectoderm [166]. This expression pattern is useful for regulating the growth of facial protrusions, which keeps the face growing normally.

Numerous studies have validated that mutations in WNT3 and WNT9b are significantly correlated with non-syndromic cleft lip [167, 168], underscoring the essential function of the WNT signaling pathways in upper lip fusion and facial midline closure. The classical WNT/β-catenin signaling axis is essential for various cellular processes, including the regulation of cell fate determination, cell adhesion, and cytoskeletal dynamics. This affects how CNCCs move and how facial prominences grow, which controls how the brain and face develop in embryos [169]. Moreover, the WNT pathway has been shown to be closely associated with osteoblast differentiation. Recent studies have reported that NELL-1 can interact with the WNT/β-catenin pathway to promote osteoblast differentiation [170]. Aberrant upregulation of NELL-1 is linked to craniosynostosis, while its deficiency leads to severe skeletal malformations. In zebrafish models, abnormal activation of the WNT/β-catenin pathway may also prevent CNCCs from differentiating into chondrocytes, resulting in Microtia-atresia [171].

#### BMP/TGF-β signaling pathways

The BMP/TGF-β signaling pathways control the survival, differentiation, and ability of CNCCs to become bone and cartilage [172]. Disruption of these signaling pathways may result in craniofacial malformations and a phenotype resembling suture closure [173]. It sends signals to both the classical and the non-classical Smad-dependent signaling pathway, to control how mesenchymal stem cells change as bones grow and form [174]. BMP binds to its receptors, inducing receptor phosphorylation, which subsequently activates Smad1/5/8. These Smad proteins then form a dimeric complex with Smad4, translocating into the nucleus to activate transcriptional programs [175]. Studies indicate that BMP signaling and its downstream targeting of Msx2 regulate osteogenesis, precursor cell differentiation, and odontoblast formation during early cranial development [176]. This suggests the signaling pathways play a critical role in the differentiation of early mesenchymal precursors into osteogenic and chondrogenic lineages, craniofacial bone formation, and tooth germ development.

Animal model studies demonstrate that BMP signaling influences the spatial organization and proportional coordination of facial development. For instance, BMP4 deficiency causes abnormal maxillary and mandibular development in mice, while BMP5 and BMP7 knockout mice exhibit reduced branchial arch size and craniofacial disproportion [177, 178]. Notably, the output of this pathway is regulated not only by ligands but also depends on receptor homeostasis. Rare NRSN2 variants associated with non-syndromic cleft lip and palate impair lysosome-dependent degradation of TβRI/TβRII, thereby increasing SMAD2 phosphorylation levels [179]. This suggests that disrupted receptor homeostasis may interfere with normal craniofacial development by abnormally amplifying signaling output.

#### FGF signaling pathway

The FGF signaling pathway can regulate the survival, proliferation, migration and bone differentiation of CNCCs by driving facial ectoderm and mesenchymal proliferation, and plays a central role in craniofacial development. Its abnormality is associated with premature closure of cranial sutures and facial growth defects [180, 181]. Genetic analyses of syndromic craniosynostosis have established aberrant activation of FGF signaling as a core pathogenic mechanism [182]. Dysregulated FGF signaling drives premature osteogenic commitment and terminal differentiation of synovial mesenchymal stem cells (SMSCs), resulting in pathological suture fusion and craniosynostosis. This mechanistic model is corroborated by recurrent gain-of-function mutations in FGFR2 and FGFR3 identified across multiple craniosynostosis syndromes, including Apert and Crouzon, and Muenke syndromes [183, 184].

FGFs are key developmental regulators involved in signal transduction cascades through receptor tyrosine kinases, mainly acting on extracellular signal-regulated kinases 1 and 2 (ERK1/2) [185]. In mammals, 22 FGFs have been identified by sequence homology, of which 18 are involved in the regulation of different stages and regions as secretory ligands of four FGF receptors (FGFR1-4) [186]. Research utilizing murine models demonstrates that the activation of classical downstream signaling pathways, including ERK1/2, PI3K-AKT, and PLCγ, contributes to craniofacial morphogenesis. However, the singular loss of function in any one pathway does not suffice to produce severe developmental malformations akin to those observed in Fgfr1 or Fgfr2 receptor-deficient models [187]. On the contrary, FGFR kinase-dependent but independent of signal adaptor functions, especially those regulating cell–matrix and cell–cell adhesion, are essential for midfacial growth, palatal closure, and osteochondral differentiation of NC derived mesenchyme [187]. In addition, FGF8 is a secreted signaling molecule that exhibits a highly dynamic gene expression pattern during embryonic development and is involved in the development of craniofacial structures, limbs, internal organs, and brain [188]. Abnormal FGF8 can induce abnormal embryonic development, resulting in skeletal abnormalities, cilia, and forebrain cleftless malformations [189].

#### NOTCH signaling pathway

Unlike other key intracellular signaling pathways such as WNT, the Notch signaling pathway is activated by the interaction between intercellular receptors and ligands, and is involved in the regulation of physiological processes such as tissue growth, embryonic development, and bone homeostasis [190]. Notch receptors are synthesized in the endoplasmic reticulum and transported to the cell membrane. When the receptor binds to a transmembrane ligand on the surface of adjacent cells, it triggers proteolytic cleavage of the receptor. This process is first performed by ADAM metalloproteinase, and then cleaved by the γ-secretase complex [191]. The latter cleaves and releases the COOH-terminal fragment of the receptor-Notch intracellular domain (Notch ICD), which is then transported to the nucleus [192]. When Notch ICD enters the cell nucleus, it binds to CSL and controls the transcription of genes that come after it. This important transcription factor is similar to Rbpj or CBF1 in mammals and works in the same way as the Hairless repressor protein Su(H) in Drosophila [193]. The resulting signal output is diverse and highly dependent on the cell environment.

#### Hippo signaling pathway

The Hippo pathway is a highly conserved pathway in terms of evolution and function. It is widely involved in cell proliferation, survival, cell fate determination, and regeneration processes [194, 195]. The core of this pathway is the kinase cascade. Its key downstream factor Yes-associated protein (YAP) and its homologous transcriptional co-activator with PDZ-binding motif (TAZ) are considered to be the classical effectors of this pathway, which can respond to various internal and external signals. When the Hippo signaling pathway is activated, its upstream components Mst1/2 and Salv1 are phosphorylated, which in turn activates the Lats1/2 kinase and ultimately promotes the cytoplasmic degradation of YAP and TAZ. Conversely, when the Hippo signaling pathway is inhibited, YAP and TAZ are transferred to the nucleus and form complexes with transcription factors such as the transcription enhancer activation domain (TEAD) family in the nucleus, thereby mediating various cellular biological activities regulated by Hippo signaling [196, 197]. During craniofacial development, this pathway can regulate the proliferation, migration, differentiation and osteogenesis of NCCs, and its dysfunction can lead to a variety of diseases [198, 199]. Studies have shown that patients with heterozygous nonsense mutations in the Yap1 gene exhibit mental retardation and cleft lip and palate [200], suggesting that YAP-mediated transcriptional regulation is critical for the formation of craniofacial structures. In addition, the loss of UAK2 activity caused by the weakening of Hippo signaling pathway can induce anencephaly, and this phenotype is related to the retention of Yap protein in the cytoplasm [201]. Recent studies have further demonstrated that Hippo signaling plays a critical role in cranial suture development and homeostasis. Mechanical tension promotes osteogenic commitment of SMSCs via ROCK-mediated stabilization and nuclear accumulation of TAZ, leading to transcriptional upregulation of Runx2 and other core osteogenic effectors [202].

In animal model studies, conditional knockout of Yap and Taz in the embryonic NC of mice leads to decreased proliferation and increased apoptosis of mandibular arch cells derived from E9.5 and E10.5 NC [203]. In addition, YAP also affects the BMP and WNT signaling pathways. When YAP function is enhanced, these pathways will be activated to promote the migration of NCCs in vivo [204]. On the contrary, its weakened function will inhibit the activity of the signaling pathway and further down-regulate the activity of YAP, thereby hindering cell migration [204], highlighting the key role of the Hippo signaling pathway in craniofacial development. In addition to congenital craniofacial malformations, existing studies have shown that the Hippo signaling pathway also plays an important role in a variety of oral diseases such as periodontal tissue regeneration, periodontitis and odontogenic tumors [196]. These findings enrich the biological significance of the Hippo signaling pathway in oral congenital and acquired diseases, and suggest that there may also be a molecular association between developmental abnormalities and acquired diseases.

#### RA signaling pathway

RA is an active derivative of vitamin A, known as retinol, and is an essential morphogenetic signal molecule. This signaling molecule can regulate the development of multiple organs and tissues including the body axis, spinal cord, heart, limbs, and craniofacial, and is a major regulator of embryonic development [205, 206]. RA directly regulates the transcription of downstream target genes by binding to the retinoic acid receptor/retinoid X receptor (RAR/RXR), and its signal output is highly sensitive to time and dose, and both excess or deficiency are teratogenic [207]. Recent studies have shown that reduced RA signaling caused by Rdh10 deficiency disrupts CNCCs proliferation and survival, leading to midfacial clefts and other craniofacial abnormalities, such as auditory and eye defects [208, 209]. Mechanistically, impaired RA signaling is associated with expanded SHH pathway in the developing ventral forebrain, while partial inhibition of Hh signaling can alleviate RA deficiency-associated craniofacial defects, suggesting functional crosstalk between RA and SHH pathways during midfacial development [209]. However, excessive RA is also one of the common factors that induce cleft palate in offspring, and it is also commonly used to construct a mouse cleft palate model [210]. It is worth noting that phenotypes such as RA-induced cleft palate are not completely irreversible. Studies have shown that the regulation of specific miRNAs, such as miR-124-3p and miR-340-5p, can alleviate the cleft palate phenotype caused by RA overdose to a certain extent [211], suggesting that there is functional coupling between RA signals and post-transcriptional regulatory networks. This finding also provides a new molecular perspective for understanding the interaction between environmental exposure and genetic regulation.

### Interactions between signaling pathways

Based on the complex biological environment in the body, the signaling pathways do not operate in isolation. The BMP, WNT, and FGF pathway networks synergistically regulate cell proliferation and differentiation [212]. The interaction of SHH, BMP, and FGF8 signaling pathways forms a highly coordinated molecular regulatory network to control early facial patterns and growth, while the SHH pathway is coupled with cholesterol metabolism and cilia signaling pathways, integrating morphogenesis and cell metabolism into a unified regulatory system [213, 214]. What’s more, studies have shown that SHH signal and WNT signal jointly regulate upper lip fusion [214]. With the development of technology, researchers have further confirmed that the activation of WNT signaling can affect facial morphology by affecting the expression of SHH in the forebrain and frontonasal ectoderm regions by using computer tomography and three-dimensional geometric morphometry [215]. In addition, an in vitro study of tooth development has shown that the WNT pathway can regulate the activities of Shh, FGF and BMP during tooth germination and morphogenesis to regulate various stages of tooth development [216]. In addition, as an important signaling pathway that regulates embryonic development and bone homeostasis, BMP interacts with the Notch pathway. Some reports suggest that gap signals can enhance BMP-induced osteoblastogenesis. One study found that activation of Notch signaling in adenovirus-overexpressing NICD in MC3T3-E1 cells stimulated BMP2-induced osteoblast formation, which was manifested by increased alkaline phosphatase (ALP) expressing cells and newly formed calcified nodules [217]. However, other studies about MC3T3-E1 cells have shown that if cells overexpress BMP2, stimulation of Notch targets inhibits BMP2-induced osteoblastogenesis [217]. Therefore, there is a complex relationship between the BMP and Notch signaling pathways.

However, the highly connected network also has some problems. The disruption of a singular pathway can induce nonlinear phenotypic consequences via the network interaction amplification effect. For example, genetic model studies have shown that abnormal BMP signaling during craniofacial development can lead to coordination disorders with WNT and FGF pathways, thereby inducing more severe malformations [218].

## Teratogenic mechanisms in craniofacial development

Craniofacial development is not only strictly regulated by internal genetic factors, but also highly sensitive to exogenous environmental factors [219]. Environmental factors can affect craniofacial development in a variety of ways, including maternal nutritional status, metabolic balance, drug or chemical exposure, and other exogenous stress factors [220–222]. It should be noted that different developmental time points and disturbance durations may cause malformations of different severity [223].

### Common teratogens affecting craniofacial development

Known craniofacial teratogens include various drugs, alcohol, smoking, maternal nutritional intake and metabolic disorders, exposure to high temperatures, and physicochemical factors such as ionizing radiation [224, 225]. Various teratogenic factors frequently influence outcomes via unique, albeit partially converging, molecular mechanisms. They can directly disrupt essential biological processes, including the formation and differentiation of CNCCs [34]. Besides, they may modify the expression patterns of essential regulatory genes by triggering oxidative stress, epigenetic reconfiguration, or disruptions in developmental signaling pathways, consequently resulting in craniofacial anomalies at particular phases [224]. Major environmental teratogens and molecular mechanisms affecting craniofacial development are shown in Table 4.

**Table 4:** Major environmental teratogens and molecular mechanisms affecting craniofacial development

Environmental factor	Critical developmental stage	target cells or tissues	Key molecular mechanisms	Affected pathways/regulatory factors	Representative craniofacial phenotypes	References
Valproic acid	Neural tube formation and CNCCs migration	CNCCs	Histone deacetylase inhibition, chromatin remodeling, epigenetic dysregulation, impaired CNCCs formation and migration	WNT/β-catenin, Hoxa2, Sox9, p53, NF-κB	Cleft lip and/or palate, neural tube defects, craniofacial skeletal anomalies	[226–232]
Isotretinoin	Organogenesis	Neural crest derived craniofacial mesenchyme	Dysregulated RA signaling; transcriptional misregulation of developmental genes and apoptosis and cell cycle arrest	RA-RAR/RXR axis	Retinoic acid embryopathy including craniofacial, cardiac, and CNS anomalies	[233–236]
Thalidomide	Early organogenesis	CNCCs and developing vasculature	Anti-angiogenic effects, oxidative stress; binding to cereblon E3 ubiquitin ligase complex and dysregulation of developmental regulators such as SALL4, TBX5	CRBN-SALL4/TBX5 pathway	Hemifacial microsomia and craniofacial asymmetry	[237–242]
alcohol exposure	CNCCs formation, migration, and differentiation stages	CNCCs	Reduced H3K4me3 at the Snail1 promoter, inhibited epithelial–mesenchymal transition, ubiquitin–proteasome system dysfunction, oxidative stress and dysregulation of Nrf2 and WNT signaling	Snail1, ROS/Nrf2, SHH, WNT, miRNA networks	FASD, midfacial hypoplasia, smooth philtrum, thin upper lip	[243–252]
Tobacco smoke exposure	Organogenesis	Palatal mesenchymal cells and placenta	MAPK pathway activation, oxidative stress, NF-κB–mediated inflammatory signaling, mitochondrial dysfunction and increased apoptosis	MAPK, NF-κB, NOS signaling	Non-syndromic cleft lip and/or palate	[253–260]
Folate deficiency	Early embryogenesis	CNCCs and craniofacial mesenchyme	Disruption of one-carbon metabolism, DNA methylation abnormalities	SAM cycle, DNA methylation, miRNA regulation	Cleft lip and/or palate, mandibular anomalies, NTDs	[261–266]
Vitamin A deficiency	Organogenesis	Craniofacial skeletal progenitor cells	Reduced RAR signaling, impaired transcriptional regulation of skeletal development	RA signaling	Craniofacial skeletal defects involving eye, nasal, palatal, and mandibular structures	[267]
Maternal diabetes	Early embryogenesis	CNCCs	Hyperglycemia-induced oxidative stress, inflammatory responses, excessive autophagy, ERK pathway activation, and epigenetic alterations	ERK, RA signaling, chromatin accessibility alterations	Craniofacial skeletal and cartilage abnormalities	[268–273]
Maternal obesity	Throughout gestation	Placenta and embryonic metabolic tissues	Chronic low-grade inflammation, dysregulated lipid metabolism, and placental dysfunction	Metabolic signaling and epigenetic programming	Increased risk of craniofacial malformations	[274–279]
Maternal hyperthermia	Neural tube closure and CNCCs migration stages	Placenta and embryonic epithelial tissues	Heat shock response activation, oxidative stress, mitochondrial dysfunction, and suppression of WNT/β-catenin signaling	WNT/β-catenin suppression	Craniofacial anomalies and neural tube defects	[280–285]
Ionizing radiation	Organogenesis	DNA and placental trophoblast cells	DNA damage and ROS generation, oxidative stress, and radiation-induced epigenetic alterations	DNA damage response pathways	Craniofacial malformations and neurodevelopmental deficits	[286–296]
TCDD	Palatal development and CNCCs differentiation	CNCCs, palatal shelf	AhR activation, oxidative stress, apoptosis, promoter hypermethylation	TGF-β, SHH, WNT, RA, Oct4 epigenetic regulation	Cleft palate and craniofacial dysplasia	[297–301]

*CNCCs* Cranial neural crest cells, *WNT* Wingless-type MMTV integration site family, *β-catenin* Beta-catenin, *HOXA2* Homeobox A2, *Hoxa2* Homeobox A2, *SOX9* SRY-box transcription factor 9, *Sox9* SRY-box transcription factor 9, *p53* Tumor protein 53, *NF-κB* Nuclear factor kappa-light-chain-enhancer of activated B cells, *RA* Retinoic acid, *RAR* Retinoic acid receptor, *RXR* Retinoid X receptor, *CNS* Central nervous system, *CRBN* Cereblon, *E3* E3 ubiquitin-protein ligase, *SALL4* Spalt-like transcription factor 4, *TBX5* T-box transcription factor 5, *H3K4me3* Histone H3 lysine 4 trimethylation, *Snail1* Snail family transcriptional repressor 1, *ROS* Reactive oxygen species, *Nrf2* Nuclear factor erythroid 2-related factor 2, *NRF2* Nuclear factor erythroid 2-related factor 2, *SHH* Sonic hedgehog, *miRNA* MicroRNA, *FASD* Fetal alcohol spectrum disorders, *MAPK* Mitogen-activated protein kinase, *NOS* Nitric oxide synthase, *SAM* S-adenosylmethionine, *DNA* Deoxyribonucleic acid, *NTDs* Neural tube defects, *ERK* Extracellular signal-regulated kinase, *TCDD* 2,3,7,8-tetrachlorodibenzo-p-dioxin, *AhR* Aryl hydrocarbon receptor, *TGF-β* Transforming growth factor beta, *Oct4* Octamer-binding transcription factor 4

#### Drug factors

Valproic acid (VPA) is one of the most teratogenic antiepileptic drugs in clinical practice, which has been associated with 10% of congenital malformations, including neural tube defects, skeletal malformations and cleft lip and palate. These side effects are related to drug dose [226]. Population-based cohort studies have shown that exposure to VPA during pregnancy can significantly increase the risk of craniofacial malformations such as cleft lip and palate [227]. Mechanically, VPA is a histone deacetylase inhibitor, which can regulate chromatin state and decrease the transcriptive activities of genes [228]. Animal model studies have also shown that VPA treatment during pregnancy can influence the expression patterns of cell cycle-related genes through epigenetic mechanisms to disrupt the proliferation, migration and differentiation progresses of stem cells, and thereby affect the developmental stages [229]. Given that valproic acid has a well-documented teratogenic effect on neural development, studies have shown that it can also enhance the expression of WNT1 within cells and activate the GSK-3β/β-catenin pathway [230]. Furthermore, VPA can regulate the protein levels of p53, NF-κB, Pim-1, c-Myb, Bax and Bcl-2 within cells, and to activate apoptotic signaling pathways [231]. With regard to the teratogenic mechanism of craniofacial development, VPA has been demonstrated to affect the expression of Hoxa2 and Sox9, thereby reducing the EMT and delimitation processes of CNCCs, and consequently leading to abnormal migration [232]. However, there has been a paucity of research on the specific impact of VPA on craniofacial development, and the underlying teratogenic mechanism remains to be elucidated.

Isotretinoin is a retinoid medication derived from vitamin A and is metabolized into its active form, RA, in vivo, primarily utilized for the treatment of severe cystic acne. However, it is currently classified as a teratogen [233]. It is predicted that newborns exposed to isotretinoin in utero face a 20%–35% risk of developing congenital problems such as craniofacial anomalies, cardiovascular and neurological malformations, and thymic diseases [234]. These phenotypes have different traits and are all called retinoic acid embryopathy [235]. There are many studies that have been conducted to analyze the mechanism of action of isotretinoin. It can induce apoptosis and cell cycle arrest by regulating the expression of apoptosis-related proteins TNF-related apoptosis-inducing ligand (TRAIL) and Neutropil gelatinase-associated lipocalin (NGAL). This mechanism can exert a therapeutic effect on acne when it acts on sebaceous gland cells. However, when NCCs during embryonic development are exposed to isotretinoin, the increase in cell apoptosis is the main cause of craniofacial malformations [234]. Isotretinoin can regulate genes or other signaling molecular through RA pathway, as mentioned in 3.1.7. This form then controls the transcriptional expression of many genes that are important for development through the retinoic acid pathway [236]. Excessive exposure to exogenous isotretinoin disturbs the equilibrium of these molecular pathways, ultimately resulting in craniofacial and multisystem structural anomalies.

Thalidomide was originally used as a sedative to treat the pregnancy response of pregnant women. Subsequently, it was found that it can induce severe developmental malformations, such as hemifacial microsomia of seal limbs [237]. At present, a variety of possible teratogenic mechanisms have been proposed. For example, thalidomide may cause hemorrhage and hematoma in the craniofacial region by inhibiting angiogenesis, which in turn causes abnormal development of CNCCs [238]. In addition, nitric oxide (NO) is considered to be a potential target for thalidomide, and exogenous NO has been shown to rescue thalidomide-induced limb and eye deformities by reducing oxidative stress and promoting angiogenesis [239]. At the molecular mechanism level, the thalidomide-binding protein cereblon (CRBN) is considered to be the main target of thalidomide teratogenicity, which initiates the teratogenic effect by binding to it and inhibiting the activity of ubiquitin ligase [240, 241]. In addition, recent studies have identified spalt-like transcription factor 4 (SALL4) and TBX5 as the targets of thalidomide [242]. However, due to the complex chemical properties and mechanism of action of the drug, the specific teratogenic mechanism remains to be further explored.

#### Alcohol or tobacco smoke exposure during pregnancy

Prenatal alcohol exposure causes fetal alcohol spectrum disorders, a group of non-hereditary birth defects clinically manifested as a range of cognitive, behavioral, emotional, and functional impairments, along with craniofacial abnormalities and other congenital malformations [243]. Multiple studies indicate that ethanol exposure adversely affects the formation, migration, differentiation, and survival of NCCs during embryonic development [244]. Multiple animal model studies have demonstrated that exposure to low-concentration ethanol reduces the number of migrating cells, shortens migration distances, and impairs symmetrical locomotion, indicating that ethanol exposure inhibits NCCs migration both in vivo and in vitro [245, 246]. Ethanol exposure diminishes H3K4me3 levels in the Snail1 promoter region, which is a key transcriptional factor of EMT. This can result in reduced Snail1 expression, increased E-cadherin levels, and diminished wave-like protein expression, thus inhibiting EMT [247]. Another critical developmental target of ethanol is the ubiquitin–proteasome system (UPS). It reduces proteasome peptidase activity and increases ubiquitinated protein levels, resulting in craniofacial, neural, and endodermal phenotypic abnormalities [248]. Furthermore, many signaling pathways involving miRNAs, reactive oxygen species (ROS), Nrf2, SHH and WNT play crucial roles in ethanol-induced NCCs injury, apoptosis, and Fetal alcohol spectrum disorders (FASD) development [244, 249, 250]. Particularly, recent studies have shown that paternal alcohol exposure prior to conception also induces dose-dependent alterations in offspring craniofacial morphology and symmetry [251]. The effects of alcohol on embryonic development go beyond direct maternal exposure in the uterine environment. It may also transmit complex transgenerational effects through mechanisms of sperm epigenetic modification, which contains DNA methylation pattern remodeling, covalent histone modifications, and alterations in the small RNA profile [252]. This highlights the complexity and long-term risks associated with environmental factors like alcohol exposure in craniofacial developmental abnormalities.

Tobacco smoke (TS) stands as one of the most well-documented avoidable environmental teratogens. Its association with multiple craniofacial malformations, particularly non-syndromic cleft lip and palate, has been consistently validated in both epidemiological and experimental studies [253, 254]. TS contains multiple toxic components, including carbon monoxide, nicotine, polycyclic aromatic hydrocarbons, and heavy metals. Among these, nicotine is addictive and may be the most teratogenic substance in TS [255]. Cross-species multi-omics analysis identified several biomarkers associated with craniofacial cartilage development, the mitogen-activated protein kinase (MAPK) pathway, and oxidative stress [256]. More research showed that tobacco extracts caused abnormal activation of the MAPK pathway in human embryonic palatal mesenchymal cells, which led to growth inhibition, apoptosis, and oxidative stress [256]. TS also turns on inflammatory pathways that are driven by NF-κB, stops the production of extracellular matrix, and lowers the expression of genes that are related to mitochondria or blood vessels, such as NOS1/2. This stops complement activation and lowers macrophage and monocyte signaling, which makes the placenta not work properly and affects fetal development in an indirect way [257]. Additional research indicates that exposure to secondhand smoke inhibits placental cell survival and facilitates apoptosis. This shows up as higher levels of proapoptotic markers like Fas/FasL, and lower levels of antiapoptotic markers like BCL-2, and growth regulators like IGF-1 and IGFBPs. At the same time, the level of cytochrome c, a marker of mitochondrial function, goes down [258]. Additionally, maternal exposure to TS during pregnancy impacts embryonic development not only via direct toxicological effects but may also increase the risk of malformations by enhancing genetic susceptibility [259, 260]. This is a classic example of how genes and the environment work together. It also shows how important it is for pregnant women to quit smoking and stay away from secondhand smoke to prevent craniofacial malformations.

#### Maternal endogenous factors during pregnancy

Maternal nutrition and metabolic homeostasis during pregnancy are two important internal environmental factors that affect the development of the embryo [261, 262]. At the level of maternal nutrition, a lack of folate or its synthetic form, folic acid (FA), is closely linked to a number of craniofacial malformations, including cleft lip and palate, tongue deformities, mandibular abnormalities, and neural tube defects (NTDs) [263]. Folate serves as an essential cofactor in nucleotide biosynthesis and one-carbon metabolism, thereby supporting DNA replication, repair, and methylation via the production of S-adenosylmethionine (SAM), which is the principal cellular methyl donor critical for epigenetic regulation and embryonic patterning [264]. Maternal folate supplementation exerts stage-specific and tissue-selective effects on offspring DNA methylation patterns and transcriptomic profiles [265]. Studies have demonstrated that folate deficiency contributes to NTDs pathogenesis through dysregulation of epigenetic mechanisms, including microRNA methylation [266]. These findings emphasize the importance of one-carbon metabolism and DNA methylation homeostasis in craniofacial development. Furthermore, maternal vitamin A deficiency (VAD) leads to offspring malformations of the eyes, nose, palate, parietal bone, and mandible [267]. This is associated with reduced retinoic acid receptor (RAR) activity. As RAR-mediated transcriptional regulation is essential for craniofacial skeletal development, its dysfunction leads to skeletal malformations similar to VAD craniofacial defects [267].

At the metabolic state level, numerous studies have shown that pregestational and gestational diabetes are both significantly and positively associated with an increased risk of congenital malformations [268–270]. Notably, maternal diabetes provides strong evidence that epigenetic modifications and gene regulatory networks play essential roles in orchestrating craniofacial development. Maternal hyperglycemia directly impairs the survival and migration of CNCCs by inducing oxidative stress, inflammatory responses, and excessive autophagy [271, 272]. Mechanically, hyperglycemic conditions suppress the expression of crucial CNCCs marker genes related to craniofacial osteogenic and chondrogenic differentiation. While aberrant activation of the ERK pathway may cause excessive autophagy and developmental instability [271, 272]. Recent single cell multimodal analyses have demonstrated that maternal diabetes induces broad changes in chromatin accessibility and epigenetic landscapes within embryonic craniofacial progenitor cells [273]. These changes are accompanied by aberrant activation of RA signaling and disruption of axial patterning programs. Therefore, they lead to dysregulated expression of core developmental transcription factors, such as DLX5, Six2, and Meox1, which are responsible for craniofacial cell specification, migration, and differentiation. Maternal hyperglycemia initiates a pathological cascade involving metabolic stress, epigenetic remodeling, dysregulation of signaling pathways, and CNCCs dysfunction, ultimately increasing susceptibility to craniofacial malformations. This integrated mechanism demonstrates the influence of environmental factors on gene regulatory networks and cellular behavior, leading to specific developmental defects.

Additionally, chronic low-grade inflammation and lipid metabolism disorders associated with maternal obesity can alter the embryonic and placental microenvironments. This affects the expression of regulators governing energy and lipid metabolism, leading to abnormalities in placental metabolic status, structural morphology, and function [274]. This provides a metabolic basis for craniofacial malformations., the placenta is important for the circulatory systems of maternity and fetus since it can transport metabolic signals [275]. It dynamically modulates the intake and transport of nutrients based on the mother's metabolic state, which changes the metabolic environment of the embryo [276]. This means that problems with a mother's metabolism or nutrition could affect the structure and function of the placenta, which could stop the embryo from developing properly [277]. Recent research also shows that problems with the structure and composition of the placenta can affect the development of the brain and face [278]. Placental structural problems, low oxygen levels, or problems with blood vessel development can also throw off the fetal immune system, making oxidative stress worse and the fetus more likely to get sick [277, 279].

Notably, maternal metabolic problems can have more permanent consequences on development through epigenetic pathways [302]. Multi-omics studies have revealed that impaired maternal nutrition can alter embryonic transcript expression profiles, metabolomic characteristics, and the functionality of various essential metabolic pathways. The underlying mechanisms of developmental programming are now well accepted to be mediated by epigenetic events, which rely on one-carbon metabolism and micronutrient supply [303]. Therefore, optimization of nutritional interventions during pregnancy, making metabolic monitoring more effective, and standardizing chronic disease management are important for the health of mothers.

#### Physical and chemical environmental teratogenic factors

Maternal hyperthermia exposure during pregnancy is considered to be an important but often underestimated physical teratogenic factor [280, 281]. Epidemiological studies and animal experiments have demonstrated that elevated body temperature during the early stages of embryonic development—specifically during neural tube closure and cranial nerve ridge cell migration—significantly increases the risk of craniofacial malformations, neural tube defects, and cardiac abnormalities [282]. Based on the current research, its teratogenic effect is not dependent on specific toxic molecules, but by leading to placental dysfunction, reduced uterine-placental blood flow, impaired supply of fetal metabolic substrates, activation of maternal stress response systems, and other endocrine and immune system disorders, leading to fetal growth restriction and congenital malformations [283]. Secondly, high temperature exposure mainly destroys the fine regulation of development-related signaling pathways by activating heat shock response, oxidative stress and mitochondrial dysfunction [284]. Previous studies have shown that high temperature can induce endoplasmic reticulum stress and down-regulate the WNT/β-catenin signaling pathway, thereby inhibiting cell proliferation and destroying epithelial integrity [285]. However, it is not clear whether high temperature exposure will induce congenital malformations through the WNT signaling pathway, which remains to be further explored.

High doses of ionizing radiation, as another important physical teratogenic factor, can lead to birth defects, malformations and mental retardation, and the severity of such malformations is related to radiation dose and gestational age [286, 287]. The sources of ionizing radiation are various, including decaying radioactive isotopes, cosmic rays, and artificial medical devices [288]. At present, it is believed that ionizing radiation can indirectly affect DNA by directly acting or producing free radicals. Therefore, it is widely believed that its effect on embryonic development may be closely related to DNA damage and elevated levels of reactive oxygen species [289, 290]. By in vitro photon irradiation of full-term placental trophoblast cells, it was found that high-dose radiation can induce apoptosis of placental cells and inhibit proliferation, indicating that high-dose ionizing radiation can also affect the structure and function of the placenta, which may in turn induce abnormal embryonic development [291–293]. It is worth noting that radiation-induced damage and changes have a trans-generational effect, even continuing to the F3 generation [294]. Animal experiments have shown that ionizing radiation can change the epigenetic modification of developmental genes, such as DNA methylation status, histone modification or chromatin accessibility, to affect the development of offspring [295, 296]. This suggests that environmental factors and genetic factors work together to affect embryonic development.

In addition to physical teratogenic factors, chemical environmental pollutants such as dioxins, polycyclic aromatic hydrocarbons and heavy metals also contribute significantly to craniofacial developmental abnormalities. In particular, TCDD is a highly toxic environmental pollutant and a representative dioxin compound [297]. It has been extensively studied as a model teratogen associated with craniofacial defects. TCDD primarily exerts its biological effects in an aryl hydrocarbon receptor (AhR)-dependent manner [298]. When binding ligand, AhR translocates into the nucleus and regulates the transcription of multiple downstream target genes involved in xenobiotic metabolism, oxidative stress, cell proliferation, and embryonic development. Studies have shown that exposure to TCDD during pregnancy disrupts the development of CNCCs and the morphogenesis of the palatal shelf, ultimately leading to cleft palate and other craniofacial abnormalities [299, 300]. Mechanistically, TCDD-induced teratogenicity has been associated with abnormal epithelial-mesenchymal interactions, oxidative stress, apoptosis, and dysregulation of developmental signaling pathways including TGF-β, SHH, RAR and WNT signaling pathways [298]. What’s more, AhR-mediated transcriptional alterations may further interfere with epigenetic regulatory networks. Recent studies further demonstrated that TCDD suppresses the expression of the Oct4 transcription factor, which is associated with pluripotency. This is potentially achieved through hypermethylation of the Oct4 promoter, thereby disrupting normal palatal development [301]. These findings provide further support for the involvement of epigenetic regulation and aberrant signaling pathways in the development of craniofacial abnormalities in response to TCDD exposure.

### Gene-environment interactions

In fact, non-syndromic birth defects usually show non-Mendelian inheritance, incompletely explicit or variable expression. The inconsistency between genotype and phenotype indicates that exogenous environmental factors usually affect the severity of genetic diseases, and vice versa [223]. Gene-environment interactions (GxE) refer to the interaction of genetic and environmental factors in an accumulative or synergistic manner, resulting in phenotypic effects [304]. Normal craniofacial development similarly relies on the intricate interplay and precise coordination between these two domains. For example, smoking tobacco is a known risk factor for cleft lip and palate. In a large-scale study investigating how genes and smoking affect the risk of cleft lip and palate, researchers found that non-functional and inefficient alleles of the detoxification enzymes GSTT1 and NAT2 were risk factors for cleft lip and palate [305]. Candidate gene studies have also found genetic variants that increase the risk of cleft lip and palate caused by smoking. These include genes linked to nicotine dependence, DNA repair, and cleft formation [306–308]. Prenatal alcohol exposure causes problems with brain and facial development in fetal alcohol spectrum disorders. Genetic differences in some genes make the malformation phenotype worse. When genes like CDH1 and SLC23A2 have genetic problems and are in an environment that is pro-inflammatory or stressful, it can cause problems with NC migration and apoptosis, which can lead to craniofacial malformations like cleft lip and palate [309, 310]. Research on animal models shows that changes that mess up the SHH pathway make teratogenesis more likely to happen after drinking alcohol, which means that mutant mice have a higher chance of having holoprosencephaly-like traits [311]. GxE analysis can also help researchers find more disease risk loci, which gives them new ideas for studying how diseases work and how to stop them from happening in the first place [34].

## Molecular diagnosis and clinical management

Previous sections have summarized the key molecular and cellular mechanisms involved in craniofacial development that collectively regulate critical biological processes such as CNCCs proliferation, migration, differentiation, and tissue morphogenesis. Any disruption ultimately contributes to a broad spectrum of craniofacial anomalies. Importantly, the identification of these pathogenic mechanisms has been closely associated with the continuous advancement of molecular genetic technologies and functional experimental studies. These mechanistic discoveries have not only deepened the understanding of craniofacial developmental biology, but also promoted the clinical translation of molecular diagnosis and prenatal screening strategies. Therefore, this section is designed to elaborate on the clinical and pathological significance of craniofacial development research which will discuss advances in mechanism-based molecular diagnosis, current prenatal imaging and genetic testing approaches, as well as preventive strategies and clinical risk assessment for craniofacial disorders.

### Mechanism-based molecular diagnosis of craniofacial disorders

Advances in molecular genetic technologies have greatly accelerated the identification of pathogenic genes and molecular mechanisms underlying craniofacial disorders. High-throughput genomic approaches, including chromosomal microarray analysis (CMA), targeted sequencing, WES, and whole-genome sequencing (WGS) have enabled rapid detection of disease-causing variants in patients and their families [312, 313]. These approaches have significantly increased the effectiveness of discovering potential genes causing craniofacial deformities, particularly in syndromic and genetically diverse illnesses. Through comprehensive genomic analyses of affected families, researchers have continuously identified novel disease-associated genetic variants, thereby expanding the pathogenic spectrum of craniofacial disorders and providing an important molecular basis for accurate clinical diagnosis and genetic risk assessment [314]. For instance, researchers have investigated IRF6, GRHL3, and TBX22 by Sanger or WES to identify families with VWS and X-linked cleft palate with or without ankyloglossia (CPX), discovering six pathogenic mutations in IRF6, GRHL3, and TBX22 [315]. Subsequently, functional validation experiments enable us to identify the related genetic mechanisms such as p63-IRF6-GRHL3 axis and key signaling pathway such as TGF-β [316]. Similarly, the identification of pathogenic genes associated with TCS, including genes encoding RNA polymerase I and III subunits, was largely achieved through comprehensive genetic analyses of affected patients [317]. The clinical application of genetic testing technology has increased with the identification of pathogenic genes and their corresponding molecular pathways. When risky variants are identified, targeted molecular screening, prenatal diagnosis, genetic counseling, and recurrence risk assessment can be used more efficiently in the clinical practice.

### Current clinical diagnostic methods and risk assessment

**Fig. 2:**
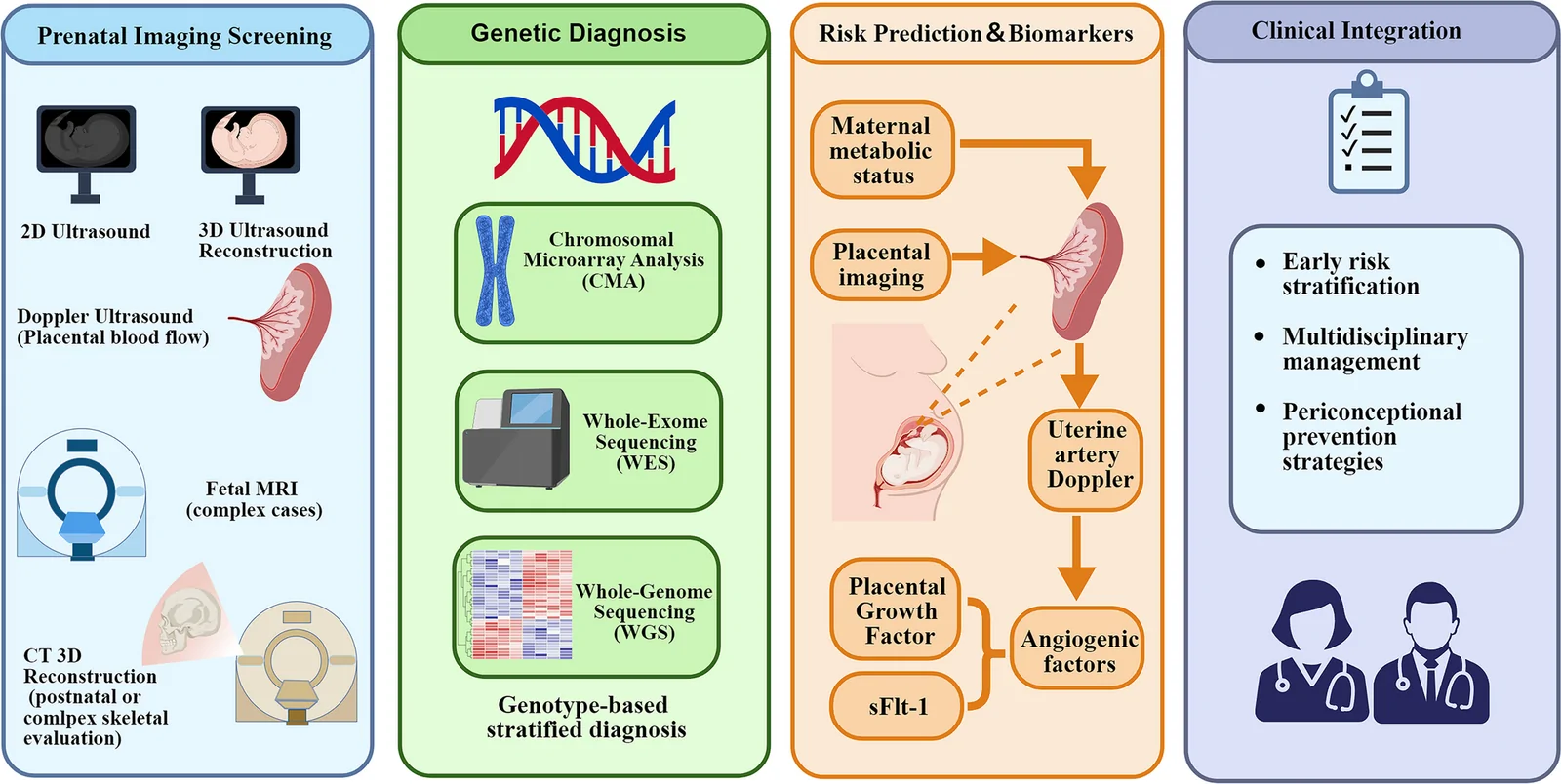
Current clinical diagnostic strategies and risk assessment approaches for craniofacial anomalies. Clinical diagnosis of craniofacial anomalies relies on the integration of prenatal imaging, genetic testing, and emerging biomarker-based risk prediction strategies. Prenatal imaging screening includes conventional 2D ultrasonography, 3D ultrasound reconstruction, and Doppler ultrasound for assessment of placental blood flow, which enable early identification of structural abnormalities during pregnancy. In complex cases, fetal MRI provides additional soft-tissue information, while 3D CT reconstruction is mainly applied postnatally for detailed evaluation of craniofacial skeletal defects. At the genetic level, CMA is widely used for detecting copy number variations associated with structural anomalies, WES and WGS facilitate the identification of pathogenic variants in suspected syndromic cases, enabling genotype-based stratified diagnosis. In addition, emerging risk-prediction approaches incorporate maternal metabolic status, placental imaging findings, uterine artery Doppler indices, and circulating angiogenic biomarkers such as placental growth factor and soluble FMS-like tyrosine kinase-1. The integration of these multimodal diagnostic approaches supports early risk stratification, multidisciplinary clinical management, and the development of periconceptional preventive strategies. 2D, Two-dimensional; 3D, Three-dimensional; MRI, Magnetic resonance imaging; CT, Computed tomography; CMA, Chromosomal microarray analysis; WES, Whole-exome sequencing; WGS, Whole-genome sequencing; FMS, Feline McDonough sarcoma; sFlt-1, Soluble FMS-like tyrosine kinase-1; FLT1, FMS-related receptor tyrosine kinase 1

The clinical diagnostic approach for craniofacial anomalies currently relies primarily on the combined application of prenatal imaging assessments and genetic testing (Fig. 2). Prenatal ultrasound screening continues to be a common method for early detection of developmental abnormalities [318]. Obstetric ultrasound utilizes grayscale imaging to observe structural morphology, umbilical cord insertion site, and uterine position, while Doppler ultrasound is used to evaluate placental blood flow and functional status [319]. Technological progress has introduced high-resolution three dimensions (3D) prenatal ultrasound and multi-slice spiral CT 3D reconstruction imaging. This enables clearer visualization of craniofacial anatomy and its associated soft tissue and skeletal malformations [320]. Additionally, a prospective cross-sectional study showed that early pregnancy ultrasound measurement of fetal facial contours can help the detection and diagnosis of congenital anomalies, with good reproducibility of measurement results [321]. Furthermore, fetal magnetic resonance imaging (MRI) provides more detailed and comprehensive soft tissue information in complex or challenging clinical cases [322, 323], which further improves the accuracy of prenatal diagnosis.

With advances of medical technology, genetic testing has become an important component of clinical diagnosis for craniofacial developmental disorders. At the genetic level, CMA can identify the copy number variations and has become the primary screening tool for structural anomalies [324]. For suspected syndromic cases, exome sequencing or whole-genome sequencing can detect pathogenic or likely pathogenic mutations [325]. With decreasing testing costs and enhanced analytical capabilities, genotype-based stratified diagnostic strategies are progressively integrating into clinical practice [326]. It has not only enhanced the diagnostic yield for syndromic craniofacial malformations but also enabled the identification of novel disease-causing genes and underlying genetic mechanisms. The integration of genomic technologies into pediatric care will enable clinicians to identify novel pathogenic variants, providing a more accurate picture of the prevalence of genetic disorders. Many neonatal genetic disorders have no distinct symptoms and our understanding of these genetic diseases is still limited. Genomic technology can detect these abnormalities during infancy. A study that specifically conducted genetic tests on infants suspected of having diseases demonstrated the significant importance of these genomic-sequencing tests in disease diagnosis and clinical decision-making [327]. Regarding the aforementioned detection methods, some researchers have examined 273 cases of TCS and further confirmed the clinical value of combining prenatal imaging examinations with molecular genetic testing [328]. The comprehensive analysis of TCS patients indicates that prenatal ultrasound examination combined with whole-exome sequencing and the detection of TCOF1 variations can significantly improve the early diagnosis rate of craniofacial abnormalities [328]. Moreover, targeted genetic screening for prevalent pathogenic variants associated with nonsyndromic hearing loss has been integrated into newborn screening programs across multiple jurisdictions [329]. This integrated approach allows for early diagnosis of infants with inherited susceptibility before the presentation of symptoms and mechanism-informed molecular testing allows for prompt diagnosis, evidence-based genetic counseling, and risk-adapted clinical management.

Apart from disease diagnosis, genetic counseling has gradually become an important component of clinical management for craniofacial developmental disorders [330]. For high-risk families who are clearly carrying pathogenic mutations or have a family history of inheritance, standardized genetic counseling can comprehensively analyze the genetic mode, recurrence risk and severity of the disease [331]. Especially in syndromic cleft lip and palate and other monogenic hereditary craniofacial diseases, genetic counseling can help patients and families correctly understand the results of genetic testing [332]. Besides, it can assist in formulating individualized follow-up and reproductive management plans, thereby improving the management level of the disease and reducing genetic risks.

In recent years, risk enrichment strategies based on maternal metabolic status, biochemical markers, or other molecular characteristics have gained attention. Development-related biomarkers hold promise in predicting potential disease risks prior to conventional imaging modalities. For example, the combination of placental imaging and uterine artery Doppler ultrasound with the assessment of angiogenic growth factors, particularly placental growth factor and soluble fms-like tyrosine kinase-1, may provide a way to detect developmental abnormalities [333–335]. However, current evidence still remains limited, with most studies confined to basic experiments. Clinical detection can be confounded and needs to be validated in prospective cohort studies and in well-designed clinical trials.

### Prevention and treatment strategies for craniofacial disorders

Primary prevention is one of the most economical and effective strategies for decreasing the incidence of congenital craniofacial malformations. The current clinical treatment of craniofacial anomalies still emphasizes multidisciplinary collaborative care. However, this therapeutic strategy is mainly aimed at pre-existing structural and functional defects and cannot reduce the occurrence of clinical anomalies. With deepening understanding of craniofacial teratogenic factors, prevention and early intervention during the periconceptional period have become critical measures for lowering the risk of craniofacial disorders.

Regarding early intervention, increasing research is focusing on the role of maternal factors in embryonic development. The concept of maternal-effect genes (MEGs), proposed in recent years, offers a new perspective for understanding developmental abnormalities originating outside the embryo itself [336]. Studies indicate that some MEGs are implicated in the development of severe embryonic and extraembryonic defects [336], including craniofacial malformations, neural tube defects, and congenital heart defects observed in Nlrp2 knockout models [337]. This indicates that maternal genetic factors, have a profound effect on embryonic development and not only the external environment. Therefore, enhancing pre-pregnancy genetic counseling, conducting genetic screening for high-risk families and researching the molecular mechanisms related to maternal effects are of great significance for achieving early prevention of birth defects.

In addition to the genetic determinants, maternal nutritional status and the intrauterine metabolic environment are critical exogenous factors affecting craniofacial development. Evidence suggests that maternal–fetal lipid pathways and placental lipid metabolism affect fetal development, demonstrating that maternal metabolic status influences embryonic development via the placenta [338, 339]. This implies that during embryonic development, it is essential to eliminate various adverse external factors for the mother and closely monitor the maternal indicators. For example, maternal metabolic monitoring, nutritional interventions, and weight management can be implemented during pregnancy [340, 341]. Besides, risk indicators should be incorporated into prenatal risk assessments to facilitate timely identification of risk factors and corresponding adjustments [342]. Clinically, such preventive measures are expected to prevent or interrupt the effects of maternal disturbances on embryonic craniofacial development, potentially significantly reducing malformation rates and alleviating disease burden. However, it must be clarified that current intervention strategies should be regarded as metabolic optimization measures with potential developmental benefits, rather than established craniofacial malformation prevention methods. Their actual impact on craniofacial development remains to be validated through clinical studies and randomized controlled trials using craniofacial phenotype as the endpoint [343].

In recent years, ncRNAs such as miRNA, lncRNA, and circRNA have been regarded as important molecules regulating craniofacial development [10, 344]. Their abnormal expression is closely related to NCCs differentiation, palatal development, and epithelial-mesenchymal interactions [344]. Small molecule RNAs, antisense oligonucleotides (ASO) and RNA delivery systems based on RNA interference technology have made positive progress in various disease studies [345, 346]. It provides new possibilities for correcting abnormal gene expression related to abnormal development in the future. In addition, epigenetic abnormalities such as DNA methylation and histone modification have certain reversibility [347]. It indicates that epigenetic treatment strategies targeting key enzymes such as DNMT or histone deacetylase (HDAC) are also considered to have potential clinical application value. However, it still needs to be further explored.

Furthermore, the development of regenerative medicine has further expanded new directions for the treatment of craniofacial deformities. Mesenchymal stem cells (MSCs), induced pluripotent stem cells (iPSCs) and NC-derived stem cells have shown excellent regenerative potential in bone, cartilage and soft tissue repair [348]. Combined with new technologies such as tissue engineering scaffolds, bioactive materials, and three-dimensional bioprinting, it is expected to achieve functional repair of complex craniofacial tissue defects [349]. At the same time, the development of gene editing technologies such as CRISPR-Cas9 provides new possibilities for precise treatment of hereditary craniofacial diseases [350]. Although current research is mainly at the experimental research and animal model stages, these methods are expected to become important intervention strategies for hereditary craniofacial deformities in the future.

Overall, the clinical significance brought about by studies related to craniofacial development is to promote the development of various clinical diagnoses, intervention measures and treatment strategies. In the future, by integrating multiple advanced technologies, it is expected to enhance the accuracy of early screening and treatment success rates for craniofacial developmental disorders. Therefore, this will not only further deepen our understanding of craniofacial development disorders and its mechanisms, but also provide more effective theoretical basis for the clinical prevention and treatment of congenital craniofacial malformations.

## Current challenges and future directions

Even though there has been a lot of progress in the last few years in understanding the molecular basis of craniofacial development, there are still many problems in this area that make it hard to turn research findings into clinical use. Craniofacial malformations are complex multifactorial disorders characterized by intricate interactions at multiple levels. These include gene regulatory networks, epigenetic mechanisms, maternal endogenous factors, and exogenous environmental exposures [351]. This complexity makes single-gene or single-factor studies insufficient for clinical applications. Craniofacial development is highly susceptible to particular temporal windows, particularly during the critical early prenatal phase [352]. In this phase, minor genetic or environmental perturbations may lead to irreversible consequences. Current clinical studies based on human samples are predominantly retrospective analyses of postnatal phenotypes, which make it difficult to determine specific teratogenic stages and integrate them with basic research. Moreover, although many teratogenic factors associated with craniofacial abnormalities have been identified, complex genetic-environmental interactions obstruct the formulation of definitive causal relationships between particular phenotypes and pathways. Furthermore, extensive studies of environmental exposures, maternal metabolic states, or lifestyle factors primarily rely on observational population data, which limits the ability to accurately pinpoint important cellular targets and molecular mechanisms [353]. Little research has been conducted on specific factors and the connections between teratogenic factors are poorly understood. This makes it difficult to compare results across studies and to correctly predict the risk of clinical illness by algorithms.

Besides, the increasing use of prenatal genetic testing and other advanced diagnostic technologies also raises substantial ethical and clinical concerns. Issues regarding informed consent, data privacy, the psychological impact on families and the correct interpretation of prenatal genetic information require careful consideration during the clinical implementation process [354]. Recent studies about prenatal counselling have further highlighted the ethical complexities of prenatal decision-making in situations of prognostic uncertainty, emotional distress and varying institutional practices [355]. Moreover, the increasing application of genome sequencing and risk prediction models in prenatal diagnosis may generate variants of uncertain significance [356], thereby complicating clinical interpretation and parental decision-making. In some cases, the limited understanding of genotype–phenotype correlations and variable disease expressivity may increase parental anxiety and contribute to potential overdiagnosis or unnecessary medical intervention.

To tackle these challenges, forthcoming research necessitates significant enhancements in study design and technical methodologies. Future research on the fundamental mechanism should establish multifactorial models of craniofacial malformations to clarify the interactions among complex factors and their teratogenic mechanisms. At the same time, clinical phenotypes should be clearly defined and connected to clinical outcomes. Moreover, time-resolved multiomics studies must be performed with specified gestational stages to systematically delineate dynamic alterations in transcriptomes, epigenomes, proteomes, and metabolomes during pivotal developmental phases [357]. Recent advances in spatial transcriptomics and single-cell sequencing technologies have further provided new opportunities for understanding the spatiotemporal regulation of craniofacial morphogenesis. Integration of spatial transcriptomics with high-resolution imaging technologies may facilitate the construction of spatially resolved molecular atlases of craniofacial primordia and associated tissues, thereby accurately identifying pathogenic molecular alterations within specific cell populations and developmental stages [358]. Besides, combining single-cell transcriptomics with spatial transcriptome studies across human developmental stages have improved our understanding of cellular heterogeneity and lineage specification. Researchers have identified distinct mesenchymal, epithelial, and CNCCs subtypes associated with normal facial morphogenesis and orofacial cleft susceptibility [359], highlighting the potential of these two advanced approaches for linking developmental cell states with human craniofacial disease risk.

For clinical evaluation, subsequent studies should develop prospective birth cohorts, improve exposure assessments, and validate findings through functional animal models to establish more rigorous causal chains. To translate fundamental research findings into clinical application, it is imperative to enhance prenatal risk predictors and integrate them with postnatal clinical phenotypes to ensure the accuracy of the research. This will help basic science get closer to being used in the clinic. Additionally, creating AI-assisted imaging detection risk prediction models and AI algorithms can enhance the accuracy and efficiency of clinical diagnosis and treatment [360]. For example, deep learning-based platforms such as U-FISH have demonstrated robust performance in detecting and decoding spatial transcriptomic signals across diverse imaging datasets with subcellular resolution [361]. This clinical approach of early prevention, diagnosis, and intervention will decrease the occurrence of craniofacial malformations, improve patient outcomes, and alleviate the burden on families and society.

Nevertheless, ethical considerations must be carefully addressed when these screening approaches are applied in prenatal medicine. It is essential to obtain informed consent from the prospective parents. Given that prenatal genetic testing results may be difficult to interpret, clear and standardized communication between doctors and families is particularly important [362]. Some researchers have proposed key components of informed consent for genetic testing and identified essential concepts that should be consistently included during the counselling process to ensure accurate and comprehensive information sharing [363, 364]. Therefore, with the continuous advancement of prenatal diagnosis technologies, establishing a standardized ethical framework and a multidisciplinary consultation system will be equally crucial for clinical practice and decision-making.

## Conclusion

**Fig. 3:**
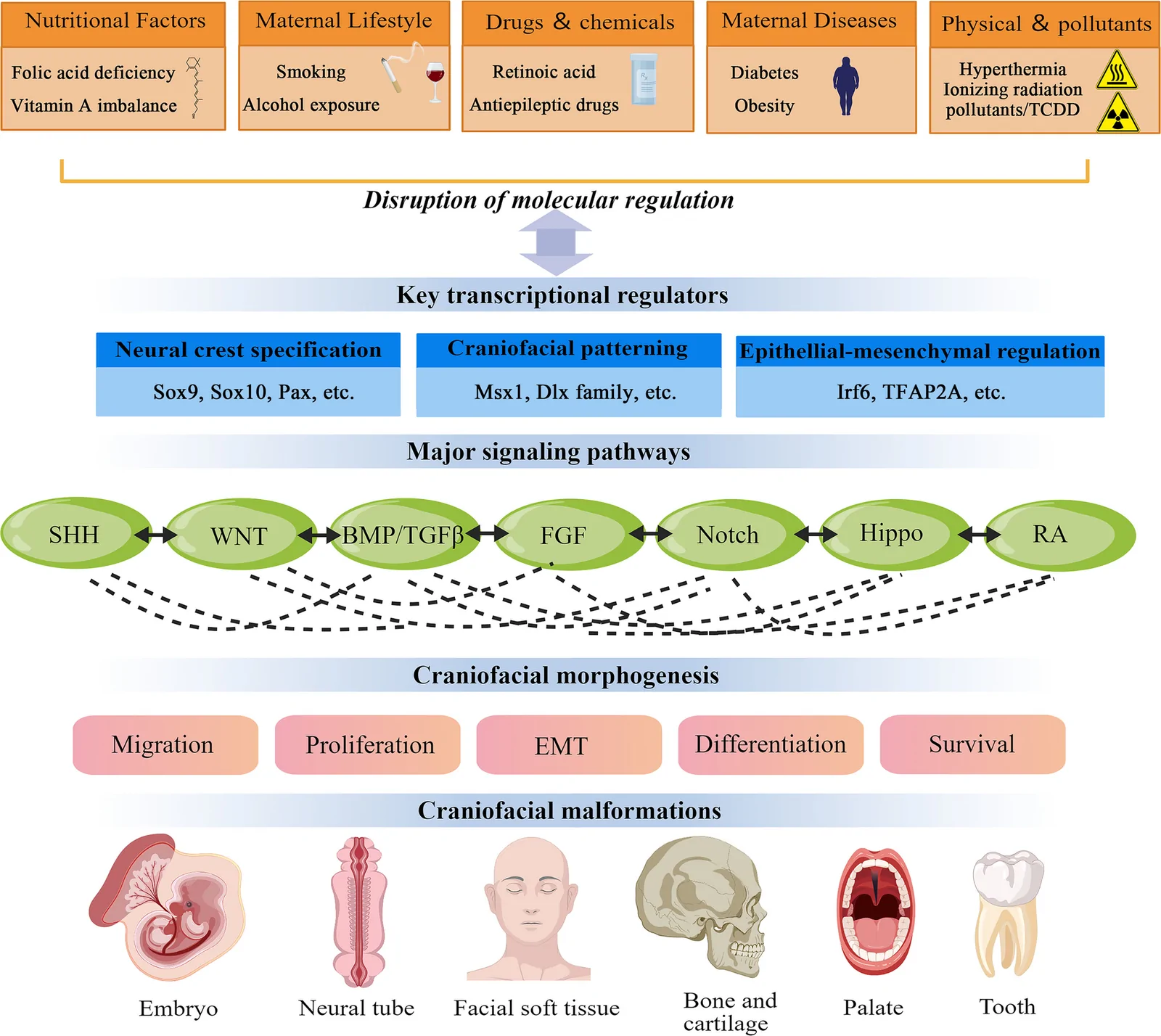
Environmental and molecular mechanisms underlying craniofacial malformations. Multiple maternal environmental factors during early pregnancy, including nutritional imbalance, adverse lifestyle behaviors, teratogenic drugs or chemicals, maternal metabolic diseases, and physical factors, can disrupt molecular regulatory networks involved in craniofacial development. These environmental influences affect key transcriptional regulators controlling neural crest cell specification, craniofacial patterning, and epithelial–mesenchymal interactions, including genes such as Sox9, Sox10, Pax, Msx1, Dlx family members, Irf6, and TFAP2A. Subsequently, multiple conserved developmental signaling pathways, which include SHH, WNT, BMP/TGF-β, FGF, Notch, Hippo, and retinoic acid pathways, interact to coordinate craniofacial morphogenesis. Dysregulation of these signaling networks can impair fundamental cellular processes such as neural crest cell migration, proliferation, differentiation, and survival, ultimately leading to a spectrum of craniofacial malformations affecting the embryo, neural tube, facial soft tissues, craniofacial skeleton, palate, and dentition. Sox9, SRY-box transcription factor 9; Sox10, SRY-box transcription factor 10; Pax, Paired box gene family; Msx1, Msh homeobox 1; Dlx, Distal-less homeobox gene family; Irf6, Interferon regulatory factor 6; TFAP2A, Transcription factor AP-2 alpha; SHH, Sonic hedgehog; WNT, Wingless-type MMTV integration site family; BMP, Bone morphogenetic protein; TGF-β, Transforming growth factor beta; FGF, Fibroblast growth factor; Notch, Notch receptor signaling pathway

Craniofacial development is a complicated biological process that requires genetic regulatory networks, signaling pathways, and environmental factors to perfectly work together at all levels (Fig. 3). Modern research is progressively transitioning from the examination of singular components to the exploration of numerous interactions. This change will help us better understand the basic rules that govern craniofacial development, make it easier to make early predictions, prevent problems before delivery, and allow for accurate treatments for congenital craniofacial defects in clinical settings.

## Data Availability

Not applicable. No datasets were generated or analyzed during the current study.

## References

[1] Roth DM, Bayona F, Baddam P, Graf D. Craniofacial Development: Neural Crest in Molecular Embryology. Head Neck Pathol. 2021;15(1):1–15. 10.1007/s12105-021-01301-z. PMC8010074 33723764

[2] Brugmann SA, Tapadia MD, Helms JA. The molecular origins of species-specific facial pattern. Curr Top Dev Biol. 2006;73:1–42. 10.1016/s0070-2153(05)73001-5. 16782454

[3] Smeriglio P, Zalc A. Cranial Neural Crest Cells Contribution to Craniofacial Bone Development and Regeneration. Curr Osteoporos Rep. 2023;21(5):624–31. 10.1007/s11914-023-00804-8. 37421571

[4] Akintoye SO, Adisa AO, Okwuosa CU, Mupparapu M. Craniofacial disorders and dysplasias: Molecular, clinical, and management perspectives. Bone Rep. 2024;20:101747. 10.1016/j.bonr.2024.101747. PMC10985038 38566929

[5] Swibel Rosenthal LH, Caballero N, Drake AF. Otolaryngologic manifestations of craniofacial syndromes. Otolaryngol Clin North Am. 2012;45(3):557–77, vii. 10.1016/j.otc.2012.03.009. 22588037

[6] Kotov A, Seal S, Alkobtawi M, Kappès V, Ruiz SM, Arbès H, et al. A time-resolved single-cell roadmap of the logic driving anterior neural crest diversification from neural border to migration stages. Proc Natl Acad Sci U S A. 2024;121(19):e2311685121. 10.1073/pnas.2311685121. PMC11087755 38683994

[7] Liao J, Huang Y, Wang Q, Chen S, Zhang C, Wang D, et al. Gene regulatory network from cranial neural crest cells to osteoblast differentiation and calvarial bone development. Cell Mol Life Sci. 2022;79(3):158. 10.1007/s00018-022-04208-2. PMC11072871 35220463

[8] Abdul NS, Shivakumar GC, Sangappa SB, Di Blasio M, Crimi S, Cicciù M, et al. Applications of artificial intelligence in the field of oral and maxillofacial pathology: a systematic review and meta-analysis. BMC Oral Health. 2024;24(1):122. 10.1186/s12903-023-03533-7. PMC10804575 38263027

[9] Adams MS, Gammill LS, Bronner-Fraser M. Discovery of transcription factors and other candidate regulators of neural crest development. Dev Dyn. 2008;237(4):1021–33. 10.1002/dvdy.21513. PMC3097249 18351660

[10] Seelan RS, Pisano MM, Greene RM. MicroRNAs as epigenetic regulators of orofacial development. Differentiation. 2022;124:1–16. 10.1016/j.diff.2022.01.002. 35144134

[11] Sauka-Spengler T, Bronner-Fraser M. A gene regulatory network orchestrates neural crest formation. Nat Rev Mol Cell Biol. 2008;9(7):557–68. 10.1038/nrm2428. 18523435

[12] Kim S, Morgunova E, Naqvi S, Goovaerts S, Bader M, Koska M, et al. DNA-guided transcription factor cooperativity shapes face and limb mesenchyme. Cell. 2024;187(3):692-711.e26. 10.1016/j.cell.2023.12.032. PMC10872279 38262408

[13] Wilderman A, VanOudenhove J, Kron J, Noonan JP, Cotney J. High-Resolution Epigenomic Atlas of Human Embryonic Craniofacial Development. Cell Rep. 2018;23(5):1581–97. 10.1016/j.celrep.2018.03.129. PMC5965702 29719267

[14] Knight RD, Javidan Y, Zhang T, Nelson S, Schilling TF. AP2-dependent signals from the ectoderm regulate craniofacial development in the zebrafish embryo. Development. 2005;132(13):3127–38. 10.1242/dev.01879. 15944192

[15] Milunsky JM, Maher TA, Zhao G, Roberts AE, Stalker HJ, Zori RT, et al. TFAP2A mutations result in branchio-oculo-facial syndrome. Am J Hum Genet. 2008;82(5):1171–7. 10.1016/j.ajhg.2008.03.005. PMC2427243 18423521

[16] Satoda M, Zhao F, Diaz GA, Burn J, Goodship J, Davidson HR, et al. Mutations in TFAP2B cause Char syndrome, a familial form of patent ductus arteriosus. Nat Genet. 2000;25(1):42–6. 10.1038/75578. 10802654

[17] Kim K, Orvis J, Stolfi A. Pax3/7 regulates neural tube closure and patterning in a non-vertebrate chordate. Front Cell Dev Biol. 2022;10:999511. 10.3389/fcell.2022.999511. PMC9511217 36172287

[18] Qiu K, Xu D, Wang L, Zhang X, Jiao N, Gong L, et al. Association analysis of single-cell RNA sequencing and proteomics reveals a vital role of Ca(2+) signaling in the determination of skeletal muscle development potential. Cells. 2020;9(4). 10.3390/cells9041045. PMC7225978 32331484

[19] Prince NB, Liang JH, Rosato TM, Lang D. PAX3: A driver of normal development and disease. Biomolecules. 2026;16(3). 10.3390/biom16030450. PMC13024339 41897383

[20] Sweat YY, Sweat M, Mansaray M, Cao H, Eliason S, Adeyemo WL, et al. Six2 regulates Pax9 expression, palatogenesis and craniofacial bone formation. Dev Biol. 2020;458(2):246–56. 10.1016/j.ydbio.2019.11.010. PMC7568837 31765609

[21] Bhol CS, Patil S, Sahu BB, Patra SK, Bhutia SK. The clinical significance and correlative signaling pathways of paired box gene 9 in development and carcinogenesis. Biochim Biophys Acta Rev Cancer. 2021;1876(1):188561. 10.1016/j.bbcan.2021.188561. 33965511

[22] Van Otterloo E, Li W, Garnett A, Cattell M, Medeiros DM, Cornell RA. Novel Tfap2-mediated control of soxE expression facilitated the evolutionary emergence of the neural crest. Development. 2012;139(4):720–30. 10.1242/dev.071308. PMC3265060 22241841

[23] Schock EN, LaBonne C. Sorting Sox: Diverse Roles for Sox Transcription Factors During Neural Crest and Craniofacial Development. Front Physiol. 2020;11:606889. 10.3389/fphys.2020.606889. PMC7793875 33424631

[24] Mori-Akiyama Y, Akiyama H, Rowitch DH, de Crombrugghe B. Sox9 is required for determination of the chondrogenic cell lineage in the cranial neural crest. Proc Natl Acad Sci U S A. 2003;100(16):9360–5. 10.1073/pnas.1631288100. PMC170923 12878728

[25] Mundell NA, Labosky PA. Neural crest stem cell multipotency requires Foxd3 to maintain neural potential and repress mesenchymal fates. Development. 2011;138(4):641–52. 10.1242/dev.054718. PMC3026411 21228004

[26] Costa R, Muccioli S, Brillo V, Bachmann M, Szabò I, Leanza L. Mitochondrial dysfunction interferes with neural crest specification through the FoxD3 transcription factor. Pharmacol Res. 2021;164:105385. 10.1016/j.phrs.2020.105385. 33348025

[27] Nieto MA. The snail superfamily of zinc-finger transcription factors. Nat Rev Mol Cell Biol. 2002;3(3):155–66. 10.1038/nrm757. 11994736

[28] Acloque H, Ocaña OH, Abad D, Stern CD, Nieto MA. Snail2 and Zeb2 repress P-cadherin to define embryonic territories in the chick embryo. Development. 2017;144(4):649–56. 10.1242/dev.142562. 28087626

[29] Losa M, Risolino M, Li B, Hart J, Quintana L, Grishina I, et al. Face morphogenesis is promoted by Pbx-dependent EMT via regulation of Snail1 during frontonasal prominence fusion. Development. 2018;145(5). 10.1242/dev.157628. PMC5868993 29437830

[30] Martínez-Alvarez C, Blanco MJ, Pérez R, Rabadán MA, Aparicio M, Resel E, et al. Snail family members and cell survival in physiological and pathological cleft palates. Dev Biol. 2004;265(1):207–18. 10.1016/j.ydbio.2003.09.022. 14697364

[31] Tu M, Ge B, Li J, Pan Y, Zhao B, Han J, et al. Emerging biological functions of Twist1 in cell differentiation. Dev Dyn. 2025;254(1):8–25. 10.1002/dvdy.736. 39254141

[32] Bertol JW, Johnston S, Ahmed R, Xie VK, Hubka KM, Cruz L, et al. TWIST1 interacts with β/δ-catenins during neural tube development and regulates fate transition in cranial neural crest cells. Development. 2022;149(15). 10.1242/dev.200068. PMC9440756 35781329

[33] Adhikari N, Wu Z, Huang Y, Lan Y, Jiang R. Twist1 Acts Upstream of the Dlx5-Hand2 Pathway to Pattern the Mammalian Jaw. J Dent Res. 2025;104(3):310–9. 10.1177/00220345241291527. PMC12331465 39707586

[34] Erdogan-Yildirim Z, Carlson JC, Mukhopadhyay N, Leslie-Clarkson EJ, Padilla CD, Murray JC, et al. Gene-by-environment interactions involving maternal exposures with orofacial cleft risk in Filipinos. Genes (Basel). 2025;16(8). 10.3390/genes16080876. PMC12386167 40869924

[35] Da Fontoura CSG, Eliason S, Amendt BA, Petrin AL, Moreno Uribe LM. Exploring the role of TWIST1 in malocclusion and craniofacial morphology. Front Physiol. 2026;17:1749243. 10.3389/fphys.2026.1749243. PMC12952720 41778196

[36] Bildsoe H, Loebel DA, Jones VJ, Hor AC, Braithwaite AW, Chen YT, et al. The mesenchymal architecture of the cranial mesoderm of mouse embryos is disrupted by the loss of Twist1 function. Dev Biol. 2013;374(2):295–307. 10.1016/j.ydbio.2012.12.004. PMC6644677 23261931

[37] Kim S, Twigg SRF, Scanlon VA, Chandra A, Hansen TJ, Alsubait A, et al. Localized TWIST1 and TWIST2 basic domain substitutions cause four distinct human diseases that can be modeled in Caenorhabditis elegans. Hum Mol Genet. 2017;26(11):2118–32. 10.1093/hmg/ddx107. PMC5438873 28369379

[38] Qin Q, Xu Y, He T, Qin C, Xu J. Normal and disease-related biological functions of Twist1 and underlying molecular mechanisms. Cell Res. 2012;22(1):90–106. 10.1038/cr.2011.144. PMC3351934 21876555

[39] Marchegiani S, Davis T, Tessadori F, van Haaften G, Brancati F, Hoischen A, et al. Recurrent Mutations in the Basic Domain of TWIST2 Cause Ablepharon Macrostomia and Barber-Say Syndromes. Am J Hum Genet. 2015;97(1):99–110. 10.1016/j.ajhg.2015.05.017. PMC4572501 26119818

[40] Shin JO, Nakagawa E, Kim EJ, Cho KW, Lee JM, Cho SW, et al. miR-200b regulates cell migration via Zeb family during mouse palate development. Histochem Cell Biol. 2012;137(4):459–70. 10.1007/s00418-012-0915-6. 22261924

[41] Jin JZ, Li Q, Higashi Y, Darling DS, Ding J. Analysis of Zfhx1a mutant mice reveals palatal shelf contact-independent medial edge epithelial differentiation during palate fusion. Cell Tissue Res. 2008;333(1):29–38. 10.1007/s00441-008-0612-x. PMC2516965 18470539

[42] Vandewalle C, Van Roy F, Berx G. The role of the ZEB family of transcription factors in development and disease. Cell Mol Life Sci. 2009;66(5):773–87. 10.1007/s00018-008-8465-8. PMC11131515 19011757

[43] Charney RM, Prasad MS, Juan-Sing C, Patel LJ, Hernandez JC, Wu J, et al. Mowat-Wilson syndrome factor ZEB2 controls early formation of human neural crest through BMP signaling modulation. Stem Cell Reports. 2023;18(11):2254–67. 10.1016/j.stemcr.2023.10.002. PMC10679662 37890485

[44] Hossain WA, St Peter C, Lovell S, Rafi SK, Butler MG. ZEB2 Gene pathogenic variants across protein-coding regions and impact on clinical manifestations: a review. Int J Mol Sci. 2025;26(3). 10.3390/ijms26031307. PMC11818587 39941075

[45] Tan Y, Testa JR. DLX Genes: Roles in Development and Cancer. Cancers (Basel). 2021;13(12). 10.3390/cancers13123005. PMC8232755 34203994

[46] Acampora D, Merlo GR, Paleari L, Zerega B, Postiglione MP, Mantero S, et al. Craniofacial, vestibular and bone defects in mice lacking the Distal-less-related gene Dlx5. Development. 1999;126(17):3795–809. 10.1242/dev.126.17.3795. 10433909

[47] Alappat S, Zhang ZY, Chen YP. Msx homeobox gene family and craniofacial development. Cell Res. 2003;13(6):429–42. 10.1038/sj.cr.7290185. 14728799

[48] Tribulo C, Aybar MJ, Nguyen VH, Mullins MC, Mayor R. Regulation of Msx genes by a Bmp gradient is essential for neural crest specification. Development. 2003;130(26):6441–52. 10.1242/dev.00878. 14627721

[49] Liang J, Von den Hoff J, Lange J, Ren Y, Bian Z, Carels CE. MSX1 mutations and associated disease phenotypes: genotype-phenotype relations. Eur J Hum Genet. 2016;24(12):1663–70. 10.1038/ejhg.2016.78. PMC5117928 27381090

[50] Carroll SH, Macias Trevino C, Li EB, Kawasaki K, Myers N, Hallett SA, et al. An Irf6-Esrp1/2 regulatory axis controls midface morphogenesis in vertebrates. Development. 2020;147(24). 10.1242/dev.194498. PMC7774891 33234718

[51] Iwata J, Suzuki A, Pelikan RC, Ho TV, Sanchez-Lara PA, Urata M, et al. Smad4-Irf6 genetic interaction and TGFβ-mediated IRF6 signaling cascade are crucial for palatal fusion in mice. Development. 2013;140(6):1220–30. 10.1242/dev.089615. PMC3585659 23406900

[52] Kumari P, Friedman RZ, Curtis SW, Pi L, Paraiso K, Visel A, et al. Identification of functional non-coding variants associated with orofacial cleft. Nat Commun. 2025;16(1):6545. 10.1038/s41467-025-61734-w. PMC12267437 40670354

[53] Kousa YA, Zhu H, Fakhouri WD, Lei Y, Kinoshita A, Roushangar RR, et al. The TFAP2A-IRF6-GRHL3 genetic pathway is conserved in neurulation. Hum Mol Genet. 2019;28(10):1726–37. 10.1093/hmg/ddz010. PMC6494790 30689861

[54] Schock EN, York JR, LaBonne C. The developmental and evolutionary origins of cellular pluripotency in the vertebrate neural crest. Semin Cell Dev Biol. 2023;138:36–44. 10.1016/j.semcdb.2022.04.008. PMC11513157 35534333

[55] Ducos B, Bensimon D, Scerbo P. Vertebrate cell differentiation, evolution, and diseases: the vertebrate-specific developmental potential guardians VENTX/NANOG and POU5/OCT4 enter the stage. Cells. 2022;11(15). 10.3390/cells11152299. PMC9331430 35892595

[56] Rothstein M, Simoes-Costa M. Heterodimerization of TFAP2 pioneer factors drives epigenomic remodeling during neural crest specification. Genome Res. 2020;30(1):35–48. 10.1101/gr.249680.119. PMC6961570 31848212

[57] Pla P, Monsoro-Burq AH. The neural border: Induction, specification and maturation of the territory that generates neural crest cells. Dev Biol. 2018;444(Suppl 1):S36-s46. 10.1016/j.ydbio.2018.05.018. 29852131

[58] Van Otterloo E, Li H, Jones K L, Williams T. AP-2α and AP-2β cooperatively orchestrate homeobox gene expression during branchial arch patterning. Development. 2018;145(2). 10.1242/dev.157438. PMC5825845 29229773

[59] Monsoro-Burq AH. PAX transcription factors in neural crest development. Semin Cell Dev Biol. 2015;44:87–96. 10.1016/j.semcdb.2015.09.015. 26410165

[60] Wang K, Li N, Liu W, Li YG, Ren F. Genetic architecture of craniofacial morphogenesis: roles of PAX3, PAX7, and PAX9. Front Cell Dev Biol. 2026;14:1786426. 10.3389/fcell.2026.1786426. PMC13171511 42148305

[61] Hong CS, Saint-Jeannet JP. Sox proteins and neural crest development. Semin Cell Dev Biol. 2005;16(6):694–703. 10.1016/j.semcdb.2005.06.005. 16039883

[62] Akhurst RJ. From shape-shifting embryonic cells to oncology: The fascinating history of epithelial mesenchymal transition. Semin Cancer Biol. 2023;96:100–14. 10.1016/j.semcancer.2023.10.003. PMC10883734 37852342

[63] Guarino M. EMT: from embryogenesis, through cancer progression, to the development of carcinosarcoma. Differentiation. 2025;146:100903. 10.1016/j.diff.2025.100903. 40902315

[64] den Hollander P, Maddela JJ, Mani SA. Spatial and Temporal Relationship between Epithelial-Mesenchymal Transition (EMT) and Stem Cells in Cancer. Clin Chem. 2024;70(1):190–205. 10.1093/clinchem/hvad197. PMC11246550 38175600

[65] Kalluri R, Weinberg RA. The basics of epithelial-mesenchymal transition. J Clin Invest. 2009;119(6):1420–8. 10.1172/jci39104. PMC2689101 19487818

[66] Lamouille S, Xu J, Derynck R. Molecular mechanisms of epithelial-mesenchymal transition. Nat Rev Mol Cell Biol. 2014;15(3):178–96. 10.1038/nrm3758. PMC4240281 24556840

[67] Park MK, Lee H, Lee CH. Post-translational modification of ZEB family members in cancer progression. Int J Mol Sci. 2022;23(23). 10.3390/ijms232315127. PMC9737314 36499447

[68] Gou Y, Zhang T, Xu J. Transcription Factors in Craniofacial Development: From Receptor Signaling to Transcriptional and Epigenetic Regulation. Curr Top Dev Biol. 2015;115:377–410. 10.1016/bs.ctdb.2015.07.009. 26589933

[69] Tavares AL, Artinger KB, Clouthier DE. Regulating Craniofacial Development at the 3’ End: MicroRNAs and Their Function in Facial Morphogenesis. Curr Top Dev Biol. 2015;115:335–75. 10.1016/bs.ctdb.2015.08.001. 26589932

[70] Yoshioka H, Li A, Suzuki A, Ramakrishnan SS, Zhao Z, Iwata J. Identification of microRNAs and gene regulatory networks in cleft lip common in humans and mice. Hum Mol Genet. 2021;30(19):1881–93. 10.1093/hmg/ddab151. PMC8444451 34104955

[71] Yan F, Simon LM, Suzuki A, Iwaya C, Jia P, Iwata J, et al. Spatiotemporal MicroRNA-Gene Expression Network Related to Orofacial Clefts. J Dent Res. 2022;101(11):1398–407. 10.1177/00220345221105816. PMC9516630 35774010

[72] Yoshioka H, Jun G, Suzuki A, Iwata J. Dexamethasone suppresses palatal cell proliferation through miR-130a-3p. Int J Mol Sci. 2021;22(22). 10.3390/ijms222212453. PMC8621257 34830336

[73] Iwaya C, Suzuki A, Iwata J. Overexpression of miR-320–3p, miR-381–3p, and miR-27a-3p suppresses genes related to midline facial cleft in mouse cranial neural crest cells. Int J Mol Sci. 2025;26(21). 10.3390/ijms262110730. PMC12609918 41226766

[74] Antonaci M, Wheeler GN. MicroRNAs in neural crest development and neurocristopathies. Biochem Soc Trans. 2022;50(2):965–74. 10.1042/bst20210828. PMC9162459 35383827

[75] Richbourg HA, Hu DP, Xu Y, Barczak AJ, Marcucio RS. miR-199 family contributes to regulation of sonic hedgehog expression during craniofacial development. Dev Dyn. 2020;249(9):1062–76. 10.1002/dvdy.191. PMC7484444 32391617

[76] Kennedy EM, Hermetz K, Burt A, Pei D, Koestler DC, Hao K, et al. Placental microRNAs relate to early childhood growth trajectories. Pediatr Res. 2023;94(1):341–8. 10.1038/s41390-022-02386-0. PMC10183479 36380070

[77] Dong S, Wang L, Kato H, Hirofuji S, Zhou Z, Ito Y, et al. MECP2 Insufficiency Attenuates RUNX2-Dependent Osteoblast Differentiation via miR-126-3p/DKK1-Mediated Canonical Wnt Signaling Inhibition in Rett Syndrome. Faseb j. 2026;40(4):e71570. 10.1096/fj.202503014RR. PMC12908107 41693618

[78] Kong C, Liu F, Yao S, Ma L. Emerging roles of RNA modifications in normal development and congenital craniofacial malformations. Epigenomics. 2025;17(18):1601–19. 10.1080/17501911.2025.2600381. PMC12826736 41404794

[79] Zhang X, Hong R, Chen W, Xu M, Wang L. The role of long noncoding RNA in major human disease. Bioorg Chem. 2019;92:103214. 10.1016/j.bioorg.2019.103214. 31499258

[80] Ng SY, Johnson R, Stanton LW. Human long non-coding RNAs promote pluripotency and neuronal differentiation by association with chromatin modifiers and transcription factors. Embo j. 2012;31(3):522–33. 10.1038/emboj.2011.459. PMC3273385 22193719

[81] Pauli A, Rinn JL, Schier AF. Non-coding RNAs as regulators of embryogenesis. Nat Rev Genet. 2011;12(2):136–49. 10.1038/nrg2904. PMC4081495 21245830

[82] El-Husseiny AA, Sayed SM, Ali NS, Fakhry MM, Salman RM, Haroun MA, et al. Decoding the modulatory role of long non-coding RNAs in embryogenesis: a comprehensive review. Mol Biol Rep. 2026;53(1). 10.1007/s11033-026-11532-4. 41779233

[83] Wu C, Liu H, Zhan Z, Zhang X, Zhang M, You J, et al. Unveiling dysregulated lncRNAs and networks in non-syndromic cleft lip with or without cleft palate pathogenesis. Sci Rep. 2024;14(1):1047. 10.1038/s41598-024-51747-8. PMC10781966 38200098

[84] He Z, Liu X, Liu X, Cui L, Yuan Y, Zhang H, et al. The role of MEG3 in the proliferation of palatal mesenchymal cells is related to the TGFβ/Smad pathway in TCDD inducing cleft palate. Toxicol Appl Pharmacol. 2021;419:115517. 10.1016/j.taap.2021.115517. 33812962

[85] Yu Z, Su H, Gao Z, Chen Y, Zhang Y, Duan W, et al. Meg3-NONO-RAR axis mediates RA pathway activation in TCDD-induced cleft palate. Toxicol. 2025;514:154099. 10.1016/j.tox.2025.154099. 40024514

[86] Li X, Gao Y, Cheng T, Lin J, Lou S, Wang L, et al. Genetic modulation of lncPSMB1 confers non-syndromic cleft lip with or without cleft palate susceptibility by promoting cell apoptosis. Commun Biol. 2025;8(1):1123. 10.1038/s42003-025-08563-1. PMC12307944 40730884

[87] Saaoud F, Drummer IVC, Shao Y, Sun Y, Lu Y, Xu K, et al. Circular RNAs are a novel type of non-coding RNAs in ROS regulation, cardiovascular metabolic inflammations and cancers. Pharmacol Ther. 2021;220:107715. 10.1016/j.pharmthera.2020.107715. PMC7969425 33141028

[88] Shen W, Sun B, Zhou C, Ming W, Zhang S, Wu X. CircFOXP1/FOXP1 promotes osteogenic differentiation in adipose-derived mesenchymal stem cells and bone regeneration in osteoporosis via miR-33a-5p. J Cell Mol Med. 2020;24(21):12513–24. 10.1111/jcmm.15792. PMC7687013 32996692

[89] Yang L, Wilusz JE, Chen LL. Biogenesis and Regulatory Roles of Circular RNAs. Annu Rev Cell Dev Biol. 2022;38:263–89. 10.1146/annurev-cellbio-120420-125117. PMC10119891 35609906

[90] Kuang L, Lei M, Li C, Guo Z, Ren Y, Zhang X, et al. Whole transcriptome sequencing reveals that non-coding RNAs are related to embryo morphogenesis and development in rabbits. Genomics. 2020;112(3):2203–12. 10.1016/j.ygeno.2019.12.016. 31881265

[91] Wang M, Wu J, Wu P, Li Y. Emerging roles of circular RNAs in stem cells. Genes Dis. 2023;10(5):1920–36. 10.1016/j.gendis.2022.05.015. PMC10363585 37492713

[92] Qian C, Xu J, Li H, Li Q, Du M, Ma L, et al. An Integrative omics approach identifies genetic effect of CircRNA on nonsyndromic cleft lip with or without cleft palate susceptibility. Cleft Palate Craniofac J. 2026; 10556656261447540. 10.1177/10556656261447540. 42116574

[93] Yu Z, Zhang Y, Wang G, Song S, Su H, Duan W, et al. Identification of competing endogenous RNA networks associated with circRNA and lncRNA in TCDD-induced cleft palate development. Toxicol Lett. 2024;401:71–81. 10.1016/j.toxlet.2024.09.001. 39270811

[94] Shu X, Cheng L, Dong Z, Shu S. Identification of circular RNA-associated competing endogenous RNA network in the development of cleft palate. J Cell Biochem. 2019;120(9):16062–74. 10.1002/jcb.28888. 31074068

[95] Gopinathan G, Diekwisch TGH. Epigenetics and early development. J Dev Biol. 2022;10(2). 10.3390/jdb10020026. PMC9225096 35735917

[96] Shull LC, Artinger KB. Epigenetic regulation of craniofacial development and disease. Birth Defects Res. 2024;116(1):e2271. 10.1002/bdr2.2271. PMC10872612 37964651

[97] Cedar H, Bergman Y. Linking DNA methylation and histone modification: patterns and paradigms. Nat Rev Genet. 2009;10(5):295–304. 10.1038/nrg2540. 19308066

[98] Turner BM. Defining an epigenetic code. Nat Cell Biol. 2007;9(1):2–6. 10.1038/ncb0107-2. 17199124

[99] Suzuki MM, Bird A. DNA methylation landscapes: provocative insights from epigenomics. Nat Rev Genet. 2008;9(6):465–76. 10.1038/nrg2341. 18463664

[100] Gillespie CA, Chowdhury A, Quinn KA, Jenkins MW, Rollins AM, Watanabe M, et al. Fundamentals of DNA methylation in development. Pediatr Res. 2025;98(2):458–69. 10.1038/s41390-024-03674-7. PMC12955732 39658604

[101] Greenberg MVC, Bourc’his D. The diverse roles of DNA methylation in mammalian development and disease. Nat Rev Mol Cell Biol. 2019;20(10):590–607. 10.1038/s41580-019-0159-6. 31399642

[102] Caldwell BA, Bartolomei MS. DNA methylation reprogramming of genomic imprints in the mammalian germline: A TET-centric view. Andrology. 2023;11(5):884–90. 10.1111/andr.13303. 36150101

[103] Zeng Y, Chen T. DNA methylation reprogramming during mammalian development. Genes (Basel). 2019;10(4). 10.3390/genes10040257. PMC6523607 30934924

[104] Yamazaki T, Hatano Y, Taniguchi R, Kobayashi N, Yamagata K. Editing DNA methylation in mammalian embryos. Int J Mol Sci. 2020;21(2). 10.3390/ijms21020637. PMC7014263 31963664

[105] Chen Z, Zhang Y. Role of Mammalian DNA Methyltransferases in Development. Annu Rev Biochem. 2020;89:135–58. 10.1146/annurev-biochem-103019-102815. 31815535

[106] Ulschmid CM, Sun MR, Jabbarpour CR, Steward AC, Rivera-González KS, Cao J, et al. Disruption of DNA methylation-mediated cranial neural crest proliferation and differentiation causes orofacial clefts in mice. Proc Natl Acad Sci U S A. 2024;121(3):e2317668121. 10.1073/pnas.2317668121. PMC10801837 38194455

[107] Sharp GC, Ho K, Davies A, Stergiakouli E, Humphries K, McArdle W, et al. Distinct DNA methylation profiles in subtypes of orofacial cleft. Clin Epigenetics. 2017;9:63. 10.1186/s13148-017-0362-2. PMC5465456 28603561

[108] Charoenvicha C, Sirimaharaj W, Khwanngern K, Chattipakorn N, Chattipakorn SC. Alterations in DNA methylation in orofacial clefts. Int J Mol Sci. 2022;23(21). 10.3390/ijms232112727. PMC9654384 36361518

[109] Bannister AJ, Kouzarides T. Regulation of chromatin by histone modifications. Cell Res. 2011;21(3):381–95. 10.1038/cr.2011.22. PMC3193420 21321607

[110] Li K, Han J, Wang Z. Histone modifications centric-regulation in osteogenic differentiation. Cell Death Discov. 2021;7(1):91. 10.1038/s41420-021-00472-6. PMC8093204 33941771

[111] Liu R, Wu J, Guo H, Yao W, Li S, Lu Y, et al. Post-translational modifications of histones: Mechanisms, biological functions, and therapeutic targets. MedComm (2020). 2023;4(3):e292. 10.1002/mco2.292. PMC10200003 37220590

[112] Liu YH, Schneider R. Histone modifications in development. Development. 2025;152(12). 10.1242/dev.204384. 40514762

[113] Deng P, Chen QM, Hong C, Wang CY. Histone methyltransferases and demethylases: regulators in balancing osteogenic and adipogenic differentiation of mesenchymal stem cells. Int J Oral Sci. 2015;7(4):197–204. 10.1038/ijos.2015.41. PMC5153596 26674421

[114] Matsukawa S, Miwata K, Asashima M, Michiue T. The requirement of histone modification by PRDM12 and Kdm4a for the development of pre-placodal ectoderm and neural crest in *Xenopus*. Dev Biol. 2015;399(1):164–76. 10.1016/j.ydbio.2014.12.028. 25576027

[115] Yao W, Hu X, Wang X. Crossing epigenetic frontiers: the intersection of novel histone modifications and diseases. Signal Transduct Target Ther. 2024;9(1):232. 10.1038/s41392-024-01918-w. PMC11403012 39278916

[116] Ho L, Crabtree GR. Chromatin remodelling during development. Nature. 2010;463(7280):474–84. 10.1038/nature08911. PMC3060774 20110991

[117] Morrison AJ. Chromatin-remodeling links metabolic signaling to gene expression. Mol Metab. 2020;38:100973. 10.1016/j.molmet.2020.100973. PMC7300377 32251664

[118] Dai Z, Dai X, Xiang Q, Feng J, Wang J, Deng Y, et al. Genome-wide analysis of interactions between ATP-dependent chromatin remodeling and histone modifications. BMC Genomics. 2009;10 304. 10.1186/1471-2164-10-304. PMC2713269 19586523

[119] Hada A, Hota SK, Luo J, Lin YC, Kale S, Shaytan AK, et al. Histone Octamer Structure Is Altered Early in ISW2 ATP-Dependent Nucleosome Remodeling. Cell Rep. 2019;28(1):282-294.e6. 10.1016/j.celrep.2019.05.106. 31269447

[120] Wang H, Anwaier G, Bai S, Liao L, Wang Y, Li S. How ATP-Dependent chromatin remodeling complexes regulate vertebrate embryonic development. Int J Mol Sci. 2026;27(2). 10.3390/ijms27020835. PMC12841152 41596483

[121] Petty E, Pillus L. Balancing chromatin remodeling and histone modifications in transcription. Trends Genet. 2013;29(11):621–9. 10.1016/j.tig.2013.06.006. PMC4408995 23870137

[122] Guillen Sacoto MJ, Tchasovnikarova IA, Torti E, Forster C, Andrew EH, Anselm I, et al. De Novo Variants in the ATPase Module of MORC2 Cause a Neurodevelopmental Disorder with Growth Retardation and Variable Craniofacial Dysmorphism. Am J Hum Genet. 2020;107(2):352–63. 10.1016/j.ajhg.2020.06.013. PMC7413887 32693025

[123] Barish S, Barakat TS, Michel BC, Mashtalir N, Phillips JB, Valencia AM, et al. BICRA, a SWI/SNF Complex Member, Is Associated with BAF-Disorder Related Phenotypes in Humans and Model Organisms. Am J Hum Genet. 2020;107(6):1096–112. 10.1016/j.ajhg.2020.11.003. PMC7820627 33232675

[124] Bi-Lin KW, Seshachalam PV, Tuoc T, Stoykova A, Ghosh S, Singh MK. Critical role of the BAF chromatin remodeling complex during murine neural crest development. PLoS Genet. 2021;17(3):e1009446. 10.1371/journal.pgen.1009446. PMC8016319 33750945

[125] Williams RM, Taylor G, Ling ITC, Candido-Ferreira I, Fountain DM, Mayes S, et al. Chromatin remodeller Chd7 is developmentally regulated in the neural crest by tissue-specific transcription factors. PLoS Biol. 2024;22(10):e3002786. 10.1371/journal.pbio.3002786. PMC11521297 39418292

[126] Marszałek-Kruk BA, Wójcicki P, Dowgierd K, Śmigiel R. Treacher collins syndrome: genetics, clinical features and management. Genes (Basel). 2021;12(9). 10.3390/genes12091392. PMC8470852 34573374

[127] Ulhaq ZS, Nurputra DK, Soraya GV, Kurniawati S, Istifiani LA, Pamungkas SA, et al. A systematic review on Treacher Collins syndrome: Correlation between molecular genetic findings and clinical severity. Clin Genet. 2023;103(2):146–55. 10.1111/cge.14243. 36203321

[128] Noden DM, Trainor PA. Relations and interactions between cranial mesoderm and neural crest populations. J Anat. 2005;207(5):575–601. 10.1111/j.1469-7580.2005.00473.x. PMC1571569 16313393

[129] Tse WK. Treacher Collins syndrome: New insights from animal models. Int J Biochem Cell Biol. 2016;81(Pt A):44–7. 10.1016/j.biocel.2016.10.016. 27777025

[130] van Gijn DR, Tucker AS, Cobourne MT. Craniofacial development: current concepts in the molecular basis of Treacher Collins syndrome. Br J Oral Maxillofac Surg. 2013;51(5):384–8. 10.1016/j.bjoms.2012.09.008. 23036831

[131] Luo M, Yu X. NBS1 facilitates preribosomal RNA biogenesis. Proc Natl Acad Sci U S A. 2025;122(11):e2422029122. 10.1073/pnas.2422029122. PMC11929472 40067889

[132] Falcon KT, Watt KEN, Dash S, Zhao R, Sakai D, Moore EL, et al. Dynamic regulation and requirement for ribosomal RNA transcription during mammalian development. Proc Natl Acad Sci U S A. 2022;119(31):e2116974119. 10.1073/pnas.2116974119. PMC9351356 35881792

[133] Silvey S, Lovell S, Butler MG. RNA Polymerase I Dysfunction Underlying Craniofacial Syndromes: Integrated Genetic Analysis Reveals Parallels to 22q11.2 Deletion Syndrome. Genes (Basel). 2025;16(9). 10.3390/genes16091063. PMC12470080 41010008

[134] Moore ER. Primary cilia: the new face of craniofacial research. Biomolecules. 2022. 10.3390/biom12121724. PMC9776107 36551151

[135] Sun Y. Regulatory role and mechanism of primary cilia in craniofacial and dental development. Zhonghua Kou Qiang Yi Xue Za Zhi. 2023;58(8):791–8. 10.3760/cma.j.cn112144-20230503-00180. 37550039

[136] Li N, Xu Y, Chen H, Lin J, AlAbdi L, Bekheirnia MR, et al. Bi-allelic variants in CEP295 cause Seckel-like syndrome presenting with primary microcephaly, developmental delay, intellectual disability, short stature, craniofacial and digital abnormalities. EBioMedicine. 2024;99:104940. 10.1016/j.ebiom.2023.104940. PMC10784679 38154379

[137] Suzuki A, Ogata K, Yoshioka H, Shim J, Wassif CA, Porter FD, et al. Disruption of Dhcr7 and Insig1/2 in cholesterol metabolism causes defects in bone formation and homeostasis through primary cilium formation. Bone Res. 2020;8:1. 10.1038/s41413-019-0078-3. PMC6946666 31934493

[138] Iwaya C, Suzuki A, Iwata J. Loss of Sc5d results in micrognathia due to a failure in osteoblast differentiation. J Adv Res. 2024;65:153–65. 10.1016/j.jare.2023.12.008. PMC11519736 38086515

[139] Alam S S, Kumar S, Beauchamp M C, Bareke E, Boucher A, Nzirorera N, et al. Snrpb is required in murine neural crest cells for proper splicing and craniofacial morphogenesis. Dis Model Mech. 2022;15(6). 10.1242/dmm.049544. PMC9235875 35593225

[140] Kumar S, Bareke E, Lee J, Carlson E, Merkuri F, Schwager EE, et al. Etiology of craniofacial and cardiac malformations in a mouse model of SF3B4-related syndromes. Proc Natl Acad Sci U S A. 2024;121(39):e2405523121. 10.1073/pnas.2405523121. PMC11441570 39292749

[141] Gerstner S, Berger-Santangelo H, Kastens G, Scholtes T, Wäschenbach S, Pauli S, et al. Fbrsl1 is required for cranial neural crest development and reflects a conserved function of the human disease-associated protein. Dis Model Mech. 2025;18(11). 10.1242/dmm.052472. PMC12690526 41283296

[142] Luo S, Sun H, Bian Q, Liu Z, Wang X. The etiology, clinical features, and treatment options of hemifacial microsomia. Oral Dis. 2023;29(6):2449–62. 10.1111/odi.14508. 36648381

[143] Timberlake AT, Griffin C, Heike CL, Hing AV, Cunningham ML, Chitayat D, et al. Haploinsufficiency of SF3B2 causes craniofacial microsomia. Nat Commun. 2021;12(1):4680. 10.1038/s41467-021-24852-9. PMC8333351 34344887

[144] Mao K, Borel C, Ansar M, Jolly A, Makrythanasis P, Froehlich C, et al. FOXI3 pathogenic variants cause one form of craniofacial microsomia. Nat Commun. 2023;14(1):2026. 10.1038/s41467-023-37703-6. PMC10090152 37041148

[145] Song W, Xia X, Fan Y, Zhang B, Chen X. Functional and genetic analyses unveil the implication of CDC27 in hemifacial microsomia. Int J Mol Sci. 2024;25(9). 10.3390/ijms25094707. PMC11083823 38731925

[146] Xia X, Song W, Zhang F, Fan Y, Zhang B, Chen X. ctdsp2 Knockout Induces Zebrafish Craniofacial Dysplasia via p53 Signaling Activation. Int J Mol Sci. 2025;26(3). 10.3390/ijms26031297. PMC11818092 39941065

[147] Vincent M, Geneviève D, Ostertag A, Marlin S, Lacombe D, Martin-Coignard D, et al. Treacher Collins syndrome: a clinical and molecular study based on a large series of patients. Genet Med. 2016;18(1):49–56. 10.1038/gim.2015.29. 25790162

[148] Kaikkonen MU, Lam MT, Glass CK. Non-coding RNAs as regulators of gene expression and epigenetics. Cardiovasc Res. 2011;90(3):430–40. 10.1093/cvr/cvr097. PMC3096308 21558279

[149] Rajderkar SS, Paraiso K, Amaral ML, Kosicki M, Cook LE, Darbellay F, et al. Dynamic enhancer landscapes in human craniofacial development. Nat Commun. 2024;15(1):2030. 10.1038/s41467-024-46396-4. PMC10917818 38448444

[150] Zhu S, Tao H, Cornell RA, Liu H. Functional Validation of Noncoding Variants Associated With Nonsyndromic Orofacial Cleft. Hum Mutat. 2025;2025:6824122. 10.1155/humu/6824122. PMC12411058 40918684

[151] Wilderman A, D’Haene E, Baetens M, Yankee TN, Winchester EW, Glidden N, et al. A distant global control region is essential for normal expression of anterior HOXA genes during mouse and human craniofacial development. Nat Commun. 2024;15(1):136. 10.1038/s41467-023-44506-2. PMC10762089 38167838

[152] Leem YE, Choi HK, Jung SY, Kim BJ, Lee KY, Yoon K, et al. Esco2 promotes neuronal differentiation by repressing Notch signaling. Cell Signal. 2011;23(11):1876–84. 10.1016/j.cellsig.2011.07.006. 21777673

[153] Estévez-Arroyo B, Gómez-Mendo I, Romero-Maroto M, Solano-Reina E, Iglesias-Linares A. Craniofacial characteristics in Van der Woude syndrome. Oral Dis. 2023;29(4):1680–91. 10.1111/odi.14187. 35286743

[154] Lam AK, David DJ, Townsend GC, Anderson PJ. Van der Woude syndrome: dentofacial features and implications for clinical practice. Aust Dent J. 2010;55(1):51–8. 10.1111/j.1834-7819.2009.01178.x. 20415912

[155] Sunny AP, Arunachal G, Danda S. Van der Woude syndrome: IRF6 mutations. Indian J Pediatr. 2019;86(11):1070–1. 10.1007/s12098-019-03058-4. 31468312

[156] Zhao Z, Cui R, Chi H, Wan T, Ma D, Zhang J, et al. A novel IRF6 gene mutation impacting the regulation of TGFβ2-AS1 in the TGFβ pathway: A mechanism in the development of Van der Woude syndrome. Front Genet. 2024;15:1397410. 10.3389/fgene.2024.1397410. PMC11188484 38903762

[157] Pan Z, Lin G, Liu H, Li G, Zhang X, Dai J. Mechanisms of ribosomopathy and phase separation-related ribosomopathy. J Zhejiang Univ Sci B. 2025;26(6):503–26. 10.1631/jzus.B2300904. PMC12219498 40571657

[158] Stuhlmiller TJ, García-Castro MI. Current perspectives of the signaling pathways directing neural crest induction. Cell Mol Life Sci. 2012;69(22):3715–37. 10.1007/s00018-012-0991-8. PMC3478512 22547091

[159] Xu J, Iyyanar PPR, Lan Y, Jiang R. Sonic hedgehog signaling in craniofacial development. Differentiation. 2023;13:60–76. 10.1016/j.diff.2023.07.002. PMC10529669 37481904

[160] Sigafoos AN, Paradise BD, Fernandez-Zapico ME. Hedgehog/GLI signaling pathway: transduction, regulation, and implications for disease. Cancers (Basel). 2021;13(14). 10.3390/cancers13143410. PMC8304605 34298625

[161] Hammond NL, Brookes KJ, Dixon MJ. Ectopic hedgehog signaling causes cleft palate and defective osteogenesis. J Dent Res. 2018;97(13):1485–93. 10.1177/0022034518785336. PMC6262265 29975848

[162] Wierbowski BM, Petrov K, Aravena L, Gu G, Xu Y, Salic A. Hedgehog Pathway Activation Requires Coreceptor-Catalyzed, Lipid-Dependent Relay of the Sonic Hedgehog Ligand. Dev Cell. 2020;55(4):450-467.e8. 10.1016/j.devcel.2020.09.017. PMC7686162 33038332

[163] Hayat R, Manzoor M, Hussain A. Wnt signaling pathway: A comprehensive review. Cell Biol Int. 2022;46(6):863–77. 10.1002/cbin.11797. 35297539

[164] Ji Y, Hao H, Reynolds K, McMahon M, Zhou CJ. Wnt signaling in neural crest ontogenesis and oncogenesis. Cells. 2019. 10.3390/cells8101173. PMC6829301 31569501

[165] Hari L, Miescher I, Shakhova O, Suter U, Chin L, Taketo M, et al. Temporal control of neural crest lineage generation by Wnt/β-catenin signaling. Development. 2012;139(12):2107–17. 10.1242/dev.073064. 22573620

[166] Siebel C, Lendahl U. Notch Signaling in Development, Tissue Homeostasis, and Disease. Physiol Rev. 2017;97(4):1235–94. 10.1152/physrev.00005.2017. 28794168

[167] Wang BQ, Gao ST, Chen K, Xu Z Q, Sun JM, Xia Y, et al. Association of the WNT3 polymorphisms and non-syndromic cleft lip with or without cleft palate: evidence from a meta-analysis. Biosci Rep. 2018;38(6). 10.1042/bsr20181676. PMC6250811 30355643

[168] Jin Y R, Han X H, Nishimori K, Ben-Avraham D, Oh Y J, Shim J W, et al. Canonical WNT/β-Catenin Signaling Activated by WNT9b and RSPO2 Cooperation Regulates Facial Morphogenesis in Mice. Front Cell Dev Biol. 2020;8 264. 10.3389/fcell.2020.00264. PMC7225269 32457899

[169] Sutton G, Kelsh RN, Scholpp S. Review: The Role of Wnt/β-Catenin Signalling in Neural Crest Development in Zebrafish. Front Cell Dev Biol. 2021;9:782445. 10.3389/fcell.2021.782445. PMC8667473 34912811

[170] Chen X, Wang H, Yu M, Kim JK, Qi H, Ha P, et al. Cumulative inactivation of Nell-1 in Wnt1 expressing cell lineages results in craniofacial skeletal hypoplasia and postnatal hydrocephalus. Cell Death Differ. 2020;27(4):1415–30. 10.1038/s41418-019-0427-1. PMC7206096 31582804

[171] Sun L, Ping L, Fan X, Fan Y, Zhang B, Chen X. amer1 regulates zebrafish craniofacial development by Interacting with the Wnt/β-Catenin Pathway. Int J Mol Sci. 2024;25(2). 10.3390/ijms25020734. PMC10815499 38255806

[172] Lei R, Zhang K, Liu K, Shao X, Ding Z, Wang F, et al. Transferrin receptor facilitates TGF-β and BMP signaling activation to control craniofacial morphogenesis. Cell Death Dis. 2016;7(6):e2282. 10.1038/cddis.2016.170. PMC5108332 27362800

[173] Fox SC, Waskiewicz AJ. Transforming growth factor beta signaling and craniofacial development: modeling human diseases in zebrafish. Front Cell Dev Biol. 2024;12:1338070. 10.3389/fcell.2024.1338070. PMC10879340 38385025

[174] Wu M, Chen G, Li YP. TGF-β and BMP signaling in osteoblast, skeletal development, and bone formation, homeostasis and disease. Bone Res. 2016;4:16009. 10.1038/boneres.2016.9. PMC4985055 27563484

[175] Hu X, Lin C, Ruan N, Huang Z, Zhang Y, Hu X. Operation of the atypical canonical bone morphogenetic protein signaling pathway during early human odontogenesis. Front Physiol. 2022;13:823275. 10.3389/fphys.2022.823275. PMC8863179 35211032

[176] Sun J, Ishii M, Ting MC, Maxson R. Foxc1 controls the growth of the murine frontal bone rudiment by direct regulation of a Bmp response threshold of Msx2. Development. 2013;140(5):1034–44. 10.1242/dev.085225. PMC3583041 23344708

[177] Solloway MJ, Robertson EJ. Early embryonic lethality in Bmp5;Bmp7 double mutant mice suggests functional redundancy within the 60A subgroup. Development. 1999;126(8):1753–68. 10.1242/dev.126.8.1753. 10079236

[178] Winnier G, Blessing M, Labosky PA, Hogan BL. Bone morphogenetic protein-4 is required for mesoderm formation and patterning in the mouse. Genes Dev. 1995;9(17):2105–16. 10.1101/gad.9.17.2105. 7657163

[179] Zheng X, Liang X, Wu X, He Q, Yin C, Jiao Y, et al. Rare variants in NRSN2 cause non-syndromic orofacial cleft through dysregulation of TGF-β signaling. Genes Dis. 2026;13(3):101865. 10.1016/j.gendis.2025.101865. PMC12854861 41624910

[180] Pereur R, Dambroise E. Insights into Craniofacial Development and Anomalies: Exploring Fgf Signaling in Zebrafish Models. Curr Osteoporos Rep. 2024;22(3):340–52. 10.1007/s11914-024-00873-3. 38739352

[181] Moosa S, Wollnik B. Altered FGF signalling in congenital craniofacial and skeletal disorders. Semin Cell Dev Biol. 2016;53:115–25. 10.1016/j.semcdb.2015.12.005. 26686047

[182] Zhao X, Erhardt S, Sung K, Wang J. FGF signaling in cranial suture development and related diseases. Front Cell Dev Biol. 2023;11:1112890. 10.3389/fcell.2023.1112890. PMC10267317 37325554

[183] Holmes G, O'Rourke C, Motch Perrine SM, Lu N, van Bakel H, Richtsmeier JT, et al. Midface and upper airway dysgenesis in FGFR2-related craniosynostosis involves multiple tissue-specific and cell cycle effects. Development. 2018;145(19). 10.1242/dev.166488. PMC6198473 30228104

[184] Agochukwu NB, Solomon BD, Gropman AL, Muenke M. Epilepsy in Muenke syndrome: FGFR3-related craniosynostosis. Pediatr Neurol. 2012;47(5):355–61. 10.1016/j.pediatrneurol.2012.07.004. PMC4133743 23044018

[185] Raina D, Fabris F, Morelli LG, Schröter C. Intermittent ERK oscillations downstream of FGF in mouse embryonic stem cells. Development. 2022;149(4). 10.1242/dev.199710. PMC8918804 35175328

[186] Ornitz DM, Itoh N. New developments in the biology of fibroblast growth factors. WIREs Mech Dis. 2022;14(4):e1549. 10.1002/wsbm.1549. PMC10115509 35142107

[187] Ray AT, Mazot P, Brewer JR, Catela C, Dinsmore CJ, Soriano P. FGF signaling regulates development by processes beyond canonical pathways. Genes Dev. 2020;34(23–24):1735–52. 10.1101/gad.342956.120. PMC7706708 33184218

[188] Hao Y, Tang S, Yuan Y, Liu R, Chen Q. Roles of FGF8 subfamily in embryogenesis and oral-maxillofacial diseases (Review). Int J Oncol. 2019;54(3):797–806. 10.3892/ijo.2019.4677. 30628659

[189] Yin H, Duan L, Wang Z, Liu L, Shen J. Fibroblast growth factor 8: Multifaceted role in development and developmental disorder. Genes Dis. 2025;12(5):101524. 10.1016/j.gendis.2025.101524. PMC12197990 40575596

[190] Zieba JT, Chen YT, Lee BH, Bae Y. Notch signaling in skeletal development, homeostasis and pathogenesis. Biomolecules. 2020;10(2). 10.3390/biom10020332. PMC7072615 32092942

[191] Six E, Ndiaye D, Laabi Y, Brou C, Gupta-Rossi N, Israel A, et al. The Notch ligand Delta1 is sequentially cleaved by an ADAM protease and gamma-secretase. Proc Natl Acad Sci U S A. 2003;100(13):7638–43. 10.1073/pnas.1230693100. PMC164639 12794186

[192] Khamaisi B, Luca VC, Blacklow SC, Sprinzak D. Functional Comparison between Endogenous and Synthetic Notch Systems. ACS Synth Biol. 2022;11(10):3343–53. 10.1021/acssynbio.2c00247. PMC9594772 36107643

[193] Xu T, Park SS, Giaimo BD, Hall D, Ferrante F, Ho DM, et al. RBPJ/CBF1 interacts with L3MBTL3/MBT1 to promote repression of Notch signaling via histone demethylase KDM1A/LSD1. Embo j. 2017;36(21):3232–3249. 10.15252/embj.201796525. PMC5666606 29030483

[194] Fu M, Hu Y, Lan T, Guan KL, Luo T, Luo M. The Hippo signalling pathway and its implications in human health and diseases. Signal Transduct Target Ther. 2022;7(1):376. 10.1038/s41392-022-01191-9. PMC9643504 36347846

[195] Wu Z, Guan KL. Hippo Signaling in Embryogenesis and Development. Trends Biochem Sci. 2021;46(1):51–63. 10.1016/j.tibs.2020.08.008. PMC7749079 32928629

[196] Chen J, Cheng J, Zhao C, Zhao B, Mi J, Li W. The Hippo pathway: a renewed insight in the craniofacial diseases and hard tissue remodeling. Int J Biol Sci. 2021;17(14):4060–72. 10.7150/ijbs.63305. PMC8495397 34671220

[197] Meng Z, Moroishi T, Guan KL. Mechanisms of Hippo pathway regulation. Genes Dev. 2016;30(1):1–17. 10.1101/gad.274027.115. PMC4701972 26728553

[198] Zhao X, Tang L, Le TP, Nguyen BH, Chen W, Zheng M, et al. Yap and Taz promote osteogenesis and prevent chondrogenesis in neural crest cells in vitro and in vivo. Sci Signal. 2022;15(757):eabn9009. 10.1126/scisignal.abn9009. PMC9938793 36282910

[199] Zhao X, Le TP, Erhardt S, Findley TO, Wang J. Hippo-Yap Pathway Orchestrates Neural Crest Ontogenesis. Front Cell Dev Biol. 2021;9:706623. 10.3389/fcell.2021.706623. PMC8298320 34307386

[200] Goodwin AF, Chen CP, Vo NT, Bush JO, Klein OD. YAP/TAZ Regulate Elevation and Bone Formation of the Mouse Secondary Palate. J Dent Res. 2020;99(12):1387–96. 10.1177/0022034520935372. PMC7580170 32623954

[201] Bonnard C, Navaratnam N, Ghosh K, Chan PW, Tan TT, Pomp O, et al. A loss-of-function NUAK2 mutation in humans causes anencephaly due to impaired Hippo-YAP signaling. J Exp Med. 2020;217(12). 10.1084/jem.20191561. PMC7953732 32845958

[202] Ye J, Wang J, Zhao J, Xia M, Wang H, Sun L, et al. RhoA/ROCK-TAZ Axis regulates bone formation within calvarial trans-sutural distraction osteogenesis. Cell Signal. 2024;121:111300. 10.1016/j.cellsig.2024.111300. 39004327

[203] Wang J, Xiao Y, Hsu CW, Martinez-Traverso IM, Zhang M, Bai Y, et al. Yap and Taz play a crucial role in neural crest-derived craniofacial development. Development. 2016;143(3):504–15. 10.1242/dev.126920. PMC4760309 26718006

[204] Kumar D, Nitzan E, Kalcheim C. YAP promotes neural crest emigration through interactions with BMP and Wnt activities. Cell Commun Signal. 2019;17(1):69. 10.1186/s12964-019-0383-x. PMC6589182 31228951

[205] Rekler D, Ofek S, Kagan S, Friedlander G, Kalcheim C. Retinoic acid, an essential component of the roof plate organizer, promotes the spatiotemporal segregation of dorsal neural fates. Development. 2024;151(19). 10.1242/dev.202973. PMC11463963 39250350

[206] Berenguer M, Duester G. Retinoic acid, RARs and early development. J Mol Endocrinol. 2022;69(4):T59-t67. 10.1530/jme-22-0041. PMC9561040 35593389

[207] Huebner H, Hartner A, Rascher W, Strick RR, Kehl S, Heindl F, et al. Expression and regulation of retinoic acid receptor responders in the human placenta. Reprod Sci. 2018;25(9):1357–70. 10.1177/1933719117746761. 29246089

[208] Williams AL, Bohnsack BL. What’s retinoic acid got to do with it? Retinoic acid regulation of the neural crest in craniofacial and ocular development. Genesis. 2019;57(7–8):e23308. 10.1002/dvg.23308. 31157952

[209] Wu Y, Kurosaka H, Wang Q, Inubushi T, Nakatsugawa K, Kikuchi M, et al. Retinoic acid deficiency underlies the etiology of midfacial defects. J Dent Res. 2022;101(6):686–94. 10.1177/00220345211062049. 35001679

[210] Peng Y, Wang XH, Su CN, Qiao WW, Gao Q, Sun XF, et al. RNA-seq analysis of palatal transcriptome changes in all-trans retinoic acid-induced cleft palate of mice. Environ Toxicol Pharmacol. 2020;80:103438. 10.1016/j.etap.2020.103438. 32569741

[211] Yoshioka H, Suzuki A, Iwaya C, Iwata J. Suppression of microRNA 124–3p and microRNA 340–5p ameliorates retinoic acid-induced cleft palate in mice. Development. 2022;149(9). 10.1242/dev.200476. PMC9148563 35420127

[212] Watanabe T, Kanai Y, Matsukawa S, Michiue T. Specific induction of cranial placode cells from *Xenopus* ectoderm by modulating the levels of BMP, Wnt, and FGF signaling. Genesis. 2015;53(10):652–9. 10.1002/dvg.22881. 26249012

[213] Thomason HA, Dixon MJ, Dixon J. Facial clefting in Tp63 deficient mice results from altered Bmp4, Fgf8 and Shh signaling. Dev Biol. 2008;321(1):273–82. 10.1016/j.ydbio.2008.06.030. 18634775

[214] Miyamoto T, Hosoba K, Itabashi T, Iwane AH, Akutsu SN, Ochiai H, et al. Insufficiency of ciliary cholesterol in hereditary Zellweger syndrome. Embo j. 2020;39(12):e103499. 10.15252/embj.2019103499. PMC7298307 32368833

[215] Marchini M, Hu D, Lo Vercio L, Young NM, Forkert ND, Hallgrímsson B, et al. Wnt Signaling Drives Correlated Changes in Facial Morphology and Brain Shape. Front Cell Dev Biol. 2021;9:644099. 10.3389/fcell.2021.644099. PMC8039397 33855022

[216] Aurrekoetxea M, Irastorza I, García-Gallastegui P, Jiménez-Rojo L, Nakamura T, Yamada Y, et al. Wnt/β-Catenin Regulates the Activity of Epiprofin/Sp6, SHH, FGF, and BMP to Coordinate the Stages of Odontogenesis. Front Cell Dev Biol. 2016;4:25. 10.3389/fcell.2016.00025. PMC4811915 27066482

[217] Pakvasa M, Haravu P, Boachie-Mensah M, Jones A, Coalson E, Liao J, et al. Notch signaling: Its essential roles in bone and craniofacial development. Genes Dis. 2021;8(1):8–24. 10.1016/j.gendis.2020.04.006. PMC7859553 33569510

[218] Díaz-Hernández ME, Galván-Hernández CI, Marín-Llera JC, Camargo-Sosa K, Bustamante M, Wischin S, et al. Activation of the WNT-BMP-FGF Regulatory Network Induces the Onset of Cell Death in Anterior Mesodermal Cells to Establish the ANZ. Front Cell Dev Biol. 2021;9:703836. 10.3389/fcell.2021.703836. PMC8606791 34820367

[219] Al-Obaidi S, Papageorgiou SN, Saade M, Caradonna KM, Kantarci A, Will L, et al. Influence of genetic and environmental factors on transverse growth. Eur J Orthod. 2025;47(2). 10.1093/ejo/cjaf003. 39963946

[220] Sui H, Du M, Chen J, Yang R, Shi B, Huang H, et al. The impact of maternal systemic diseases on the occurrence of cleft lip and palate in newborns: a narrative review. Front Public Health. 2025;13:1568140. 10.3389/fpubh.2025.1568140. PMC12354372 40823220

[221] Martinelli M, Palmieri A, Carinci F, Scapoli L. Non-syndromic Cleft Palate: An Overview on Human Genetic and Environmental Risk Factors. Front Cell Dev Biol. 2020;8:592271. 10.3389/fcell.2020.592271. PMC7606870 33195260

[222] Pisek A, McKinney CM, Muktabhant B, Pitiphat W. Maternal Metabolic Status and Orofacial Cleft Risk: A Case-Control Study in Thailand. Int Dent J. 2024;74(6):1413–23. 10.1016/j.identj.2024.02.005. PMC11551577 38614877

[223] Fitriasari S, Fiorino R, Truong TH, McKinney MC, Dixon J, Dixon M J, et al. Gene-environment interactions modulate the phenotype severity in mouse models of congenital craniofacial syndromes. J Clin Invest. 2025;135(19). 10.1172/jci181705. PMC12483615 40699891

[224] Fitriasari S, Trainor PA. Diabetes, Oxidative Stress, and DNA Damage Modulate Cranial Neural Crest Cell Development and the Phenotype Variability of Craniofacial Disorders. Front Cell Dev Biol. 2021;9:644410. 10.3389/fcell.2021.644410. PMC8174788 34095113

[225] Huang W, Wu T, Au WW, Wu K. Impact of environmental chemicals on craniofacial skeletal development: insights from investigations using zebrafish embryos. Environ Pollut. 2021;286:117541. 10.1016/j.envpol.2021.117541. 34118758

[226] Ornoy A, Echefu B, Becker M. Valproic acid in pregnancy revisited: neurobehavioral, biochemical and molecular changes affecting the embryo and fetus in humans and in animals: a narrative review. Int J Mol Sci. 2023;25(1). 10.3390/ijms25010390. PMC10779436 38203562

[227] Jackson A, Bromley R, Morrow J, Irwin B, Clayton-Smith J. In utero exposure to valproate increases the risk of isolated cleft palate. Arch Dis Child Fetal Neonatal Ed. 2016;101(3):F207–11. 10.1136/archdischild-2015-308278. 26408639

[228] Parodi C, Di Fede E, Peron A, Viganò I, Grazioli P, Castiglioni S, et al. Chromatin Imbalance as the Vertex Between Fetal Valproate Syndrome and Chromatinopathies. Front Cell Dev Biol. 2021;9:654467. 10.3389/fcell.2021.654467. PMC8093873 33959609

[229] Fujimura K, Mitsuhashi T, Shibata S, Shimozato S, Takahashi T. In Utero Exposure to Valproic Acid Induces Neocortical Dysgenesis via Dysregulation of Neural Progenitor Cell Proliferation/Differentiation. J Neurosci. 2016;36(42):10908–19. 10.1523/jneurosci.0229-16.2016. PMC6601887 27798144

[230] Park G, Jang WE, Kim S, Gonzales EL, Ji J, Choi S, et al. Dysregulation of the Wnt/β-catenin signaling pathway via Rnf146 upregulation in a VPA-induced mouse model of autism spectrum disorder. Exp Mol Med. 2023;55(8):1783–94. 10.1038/s12276-023-01065-2. PMC10474298 37524878

[231] Dawson JE, Raymond AM, Winn LM. Folic acid and pantothenic acid protection against valproic acid-induced neural tube defects in CD-1 mice. Toxicol Appl Pharmacol. 2006;211(2):124–32. 10.1016/j.taap.2005.07.008. 16112698

[232] Suzuki R, Imai H. Effect of valproic acid on the formation and migration of cranial neural crest cells at the early developmental stages in rat embryos. Congenit Anom (Kyoto). 2024;64(2):47–60. 10.1111/cga.12553. 38403785

[233] Melnik BC. Acne transcriptomics: fundamentals of acne pathogenesis and isotretinoin treatment. Cells. 2023;12(22). 10.3390/cells12222600. PMC10670572 37998335

[234] Draghici CC, Miulescu RG, Petca RC, Petca A, Dumitrașcu MC, Șandru F. Teratogenic effect of isotretinoin in both fertile females and males (Review). Exp Ther Med. 2021;21(5):534. 10.3892/etm.2021.9966. PMC8014951 33815607

[235] Mondal DSRS, Mishra S. Retinoic Acid Embryopathy. Int J Appl Basic Med Res. 2017;7(4):264–5. 10.4103/ijabmr.IJABMR_469_16. PMC5752814 29308367

[236] Nau H. Teratogenicity of isotretinoin revisited: species variation and the role of all-*trans*-retinoic acid. J Am Acad Dermatol. 2001;45(5):S183–7. 10.1067/mjd.2001.113720. 11606951

[237] Vargesson N. Thalidomide-induced limb defects: resolving a 50-year-old puzzle. BioEssays. 2009;31(12):1327–36. 10.1002/bies.200900103. 19921660

[238] Zhao Y, Chen Q, Chen L, Shen SGF, Dai J. Thalidomide leads to mandible hypoplasia through inhibiting angiogenesis and secondary hemorrhage in the fetal craniofacial region in rabbits. Toxicol Lett. 2020;319:250–5. 10.1016/j.toxlet.2019.11.023. 31778774

[239] Siamwala JH, Veeriah V, Priya MK, Rajendran S, Saran U, Sinha S, et al. Nitric oxide rescues thalidomide mediated teratogenicity. Sci Rep. 2012;2:679. 10.1038/srep00679. PMC3447183 22997553

[240] Ito T, Ando H, Handa H. Teratogenic effects of thalidomide: molecular mechanisms. Cell Mol Life Sci. 2011;68(9):1569–79. 10.1007/s00018-010-0619-9. PMC11114848 21207098

[241] Ito T, Ando H, Suzuki T, Ogura T, Hotta K, Imamura Y, et al. Identification of a primary target of thalidomide teratogenicity. Science. 2010;327(5971):1345–50. 10.1126/science.1177319. 20223979

[242] Gomes JDA, Kowalski TW, Fraga LR, Macedo GS, Sanseverino MTV, Schuler-Faccini L, et al. The role of ESCO2, SALL4 and TBX5 genes in the susceptibility to thalidomide teratogenesis. Sci Rep. 2019;9(1):11413. 10.1038/s41598-019-47739-8. PMC6684595 31388035

[243] Wozniak JR, Riley EP, Charness ME. Clinical presentation, diagnosis, and management of fetal alcohol spectrum disorder. Lancet Neurol. 2019;18(8):760–70. 10.1016/s1474-4422(19)30150-4. PMC6995665 31160204

[244] Chen SY, Kannan M. Neural crest cells and fetal alcohol spectrum disorders: mechanisms and potential targets for prevention. Pharmacol Res. 2023;194:106855. 10.1016/j.phrs.2023.106855. PMC10528842 37460002

[245] Zhang P, Wang G, Lin Z, Wu Y, Zhang J, Liu M, et al. Alcohol exposure induces chick craniofacial bone defects by negatively affecting cranial neural crest development. Toxicol Lett. 2017;281:53–64. 10.1016/j.toxlet.2017.09.010. 28919490

[246] Fan H, Li Y, Yuan F, Lu L, Liu J, Feng W, et al. Up-regulation of microRNA-34a mediates ethanol-induced impairment of neural crest cell migration in vitro and in zebrafish embryos through modulating epithelial-mesenchymal transition by targeting Snail1. Toxicol Lett. 2022;358:17–26. 10.1016/j.toxlet.2022.01.004. PMC9058190 35038560

[247] Li Y, Yuan F, Wu T, Lu L, Liu J, Feng W, et al. Sulforaphane protects against ethanol-induced apoptosis in neural crest cells through restoring epithelial-mesenchymal transition by epigenetically modulating the expression of Snail1. Biochim Biophys Acta Mol Basis Dis. 2019;1865(10):2586–94. 10.1016/j.bbadis.2019.07.002. PMC6708741 31295528

[248] Weeks O, Miller BM, Pepe-Mooney BJ, Oderberg IM, Freeburg SH, Smith CJ, et al. Embryonic alcohol exposure disrupts the ubiquitin-proteasome system. JCI Insight. 2022;7(23). 10.1172/jci.insight.156914. PMC9746913 36477359

[249] Yuan F, Yun Y, Fan H, Li Y, Lu L, Liu J, et al. MicroRNA-135a Protects Against Ethanol-Induced Apoptosis in Neural Crest Cells and Craniofacial Defects in Zebrafish by Modulating the Siah1/p38/p53 Pathway. Front Cell Dev Biol. 2020;8:583959. 10.3389/fcell.2020.583959. PMC7561719 33134300

[250] Tolosa EJ, Fernández-Zapico ME, Battiato NL, Rovasio RA. Sonic hedgehog is a chemotactic neural crest cell guide that is perturbed by ethanol exposure. Eur J Cell Biol. 2016;95(3–5):136–52. 10.1016/j.ejcb.2016.02.003. 26979762

[251] Higgins SL, Bhadsavle SS, Gaytan MN, Thomas KN, Golding MC. Chronic paternal alcohol exposures induce dose-dependent changes in offspring craniofacial shape and symmetry. Front Cell Dev Biol. 2024;12:1415653. 10.3389/fcell.2024.1415653. PMC11246915 39011393

[252] Liao H, Lu D, Reisinger SN, Mehrabadi MR, Gubert C, Hannan AJ. Epigenetic effects of paternal environmental exposures and experiences on offspring phenotypes. Trends Genet. 2025;41(9):735–61. 10.1016/j.tig.2025.04.015. 40467385

[253] Kummet CM, Moreno LM, Wilcox AJ, Romitti PA, DeRoo LA, Munger RG, et al. Passive Smoke Exposure as a Risk Factor for Oral Clefts-A Large International Population-Based Study. Am J Epidemiol. 2016;183(9):834–41. 10.1093/aje/kwv279. PMC4851990 27045073

[254] Vathulya M, Singh N, Naithani M, Kessler P. An intercontinental comparison of the influence of smoking on the occurrence of nonsyndromic cleft lip and palate: a meta-analysis and systematic review. Arch Craniofac Surg. 2024;25(2):51–61. 10.7181/acfs.2023.00437. PMC11098758 38742331

[255] Le Foll B, Piper ME, Fowler CD, Tonstad S, Bierut L, Lu L, et al. Tobacco and nicotine use. Nat Rev Dis Primers. 2022;8(1):19. 10.1038/s41572-022-00346-w. 35332148

[256] Lin Y, Li H, Zheng S, Han R, Wu K, Tang S, et al. Elucidating tobacco smoke-induced craniofacial deformities: Biomarker and MAPK signaling dysregulation unraveled by cross-species multi-omics analysis. Ecotoxicol Environ Saf. 2024;288:117343. 10.1016/j.ecoenv.2024.117343. 39549573

[257] Chou A, Hiatt O, Davidson B, Reynolds PR, Pickett BE, Arroyo JA. Vaping in pregnancy: unraveling molecular drivers of preeclampsia and fetal growth restriction. Int J Mol Sci. 2025;26(20). 10.3390/ijms262010009. PMC12562910 41155303

[258] Beck L, Kirkham MN, Shin M, Bikman BT, Reynolds PR, Arroyo JA. Impact of secondhand smoke and e-cigarette exposure on placental apoptotic and growth-regulatory proteins in mouse pregnancy. Cells. 2025;14(6). 10.3390/cells14060453. PMC11941361 40136702

[259] Durham E, Howie RN, Warren G, LaRue A, Cray J. Direct Effects of Nicotine Exposure on Murine Calvaria and Calvarial Cells. Sci Rep. 2019;9(1):3805. 10.1038/s41598-019-40796-z. PMC6405741 30846819

[260] Dickinson AJG, Turner SD, Wahl S, Kennedy AE, Wyatt BH, Howton DA. E-liquids and vanillin flavoring disrupts retinoic acid signaling and causes craniofacial defects in Xenopus embryos. Dev Biol. 2022;481:14–29. 10.1016/j.ydbio.2021.09.004. PMC8665092 34543654

[261] Stephenson J, Heslehurst N, Hall J, Schoenaker D, Hutchinson J, Cade JE, et al. Before the beginning: nutrition and lifestyle in the preconception period and its importance for future health. Lancet. 2018;391(10132):1830–41. 10.1016/s0140-6736(18)30311-8. PMC6075697 29673873

[262] Bowman CE, Arany Z, Wolfgang MJ. Regulation of maternal-fetal metabolic communication. Cell Mol Life Sci. 2021;78(4):1455–86. 10.1007/s00018-020-03674-w. PMC7904600 33084944

[263] Maldonado E, Martínez-Sanz E, Partearroyo T, Varela-Moreiras G, Pérez-Miguelsanz J. Maternal folic acid deficiency is associated to developing nasal and palate malformations in mice. Nutrients. 2021;13(1). 10.3390/nu13010251. PMC7830789 33467180

[264] Seyoum TF. The concept of folic acid supplementation and its role in prevention of neural tube defect among pregnant women: PRISMA. Medicine (Baltimore). 2024;103(19):e38154. 10.1097/md.0000000000038154. PMC11081602 38728462

[265] Ly A, Ishiguro L, Kim D, Im D, Kim SE, Sohn KJ, et al. Maternal folic acid supplementation modulates DNA methylation and gene expression in the rat offspring in a gestation period-dependent and organ-specific manner. J Nutr Biochem. 2016;33:103–10. 10.1016/j.jnutbio.2016.03.018. 27152636

[266] Wang L, Shangguan S, Xin Y, Chang S, Wang Z, Lu X, et al. Folate deficiency disturbs hsa-let-7 g level through methylation regulation in neural tube defects. J Cell Mol Med. 2017;21(12):3244–53. 10.1111/jcmm.13228. PMC5706510 28631291

[267] Dai Q, Sun S, Jin A, Gong X, Xu H, Yang Y, et al. Osteoblastic RAR Inhibition Causes VAD-Like Craniofacial Skeletal Deformity. J Dent Res. 2023;102(6):667–77. 10.1177/00220345231151691. 37036085

[268] Zhang TN, Huang XM, Zhao XY, Wang W, Wen R, Gao SY. Risks of specific congenital anomalies in offspring of women with diabetes: A systematic review and meta-analysis of population-based studies including over 80 million births. PLoS Med. 2022;19(2):e1003900. 10.1371/journal.pmed.1003900. PMC8806075 35104296

[269] Hong S, Lee KA, Jung YM, Jeong H, Sung JH, Seol HJ, et al. Fetal malformations in women with pregestational diabetes mellitus based on pre-pregnancy fasting plasma glucose levels. Sci Rep. 2025;15(1):41128. 10.1038/s41598-025-24954-0. PMC12635161 41266559

[270] Rotem RS, Weisskopf MG, Bateman B, Huybrechts K, Hernández-Diáz S. Maternal periconception hyperglycemia, preconception diabetes, and risk of major congenital malformations in offspring. Hum Reprod. 2024;39(12):2816–29. 10.1093/humrep/deae233. PMC11630054 39406385

[271] Wang XY, Li S, Wang G, Ma ZL, Chuai M, Cao L, et al. High glucose environment inhibits cranial neural crest survival by activating excessive autophagy in the chick embryo. Sci Rep. 2015;5:18321. 10.1038/srep18321. PMC4680872 26671447

[272] Chen X, Huang Y, Yang X, Lu H, Yang J. The molecular mechanism of craniofacial cartilage deformity induced by high glucose in zebrafish. Curr Issues Mol Biol. 2025;47(9). 10.3390/cimb47090687. PMC12468894 41020809

[273] Nishino T, Ranade SS, Pelonero A, van Soldt BJ, Ye L, Alexanian M, et al. Single Cell Multimodal Analyses Reveal Epigenomic and Transcriptomic Basis for Birth Defects in Maternal Diabetes. Nat Cardiovasc Res. 2023;2(12):1190–203. 10.1038/s44161-023-00367-y. PMC11343316 39183978

[274] de Souza LB, Sanches APV, Ferreira MS, de Oliveira JL, Cleal JK, Ignacio-Souza L. Maternal-placental axis and its impact on fetal outcomes, metabolism, and development. Biochim Biophys Acta Mol Basis Dis. 2024;1870(1):166855. 10.1016/j.bbadis.2023.166855. 37633470

[275] Brett KE, Ferraro ZM, Yockell-Lelievre J, Gruslin A, Adamo KB. Maternal-fetal nutrient transport in pregnancy pathologies: the role of the placenta. Int J Mol Sci. 2014;15(9):16153–85. 10.3390/ijms150916153. PMC4200776 25222554

[276] Satterfield MC, Edwards AK, Bazer FW, Dunlap KA, Steinhauser CB, Wu G. Placental adaptation to maternal malnutrition. Reproduction. 2021;162(4):R73-r83. 10.1530/rep-21-0179. 34314369

[277] Phuthong S, Reyes-Hernández CG, Rodríguez-Rodríguez P, Ramiro-Cortijo D, Gil-Ortega M, González-Blázquez R, et al. Sex Differences in placental protein expression and efficiency in a rat model of fetal programming induced by maternal undernutrition. Int J Mol Sci. 2020;22(1). 10.3390/ijms22010237. PMC7795805 33379399

[278] Carver AJ, Dunnwald M, Stevens HE. A head start: The relationship of placental factors to craniofacial and brain development. Dev Dyn. 2025;254(10):1096–114. 10.1002/dvdy.70018. PMC12353629 40105397

[279] Vangrieken P, Remels AHV, Al-Nasiry S, Bast A, Janssen GMJ, von Rango U, et al. Placental hypoxia-induced alterations in vascular function, morphology, and endothelial barrier integrity. Hypertens Res. 2020;43(12):1361–74. 10.1038/s41440-020-0528-8. 32733105

[280] Conway F, Portela A, Filippi V, Chou D, Kovats S. Climate change, air pollution and maternal and newborn health: An overview of reviews of health outcomes. J Glob Health. 2024;14:04128. 10.7189/jogh.14.04128. PMC11117177 38785109

[281] Zhang Y, Yu C, Wang L. Temperature exposure during pregnancy and birth outcomes: an updated systematic review of epidemiological evidence. Environ Pollut. 2017;225:700–12. 10.1016/j.envpol.2017.02.066. 28284544

[282] Padmanabhan R, Al-Menhali NM, Ahmed I, Kataya HH, Ayoub MA. Histological, histochemical and electron microscopic changes of the placenta induced by maternal exposure to hyperthermia in the rat. Int J Hyperthermia. 2005;21(1):29–44. 10.1080/02656730410001716614. 15764349

[283] Cowell W, Ard N, Herrera T, Medley EA, Trasande L. Ambient temperature, heat stress and fetal growth: A review of placenta-mediated mechanisms. Mol Cell Endocrinol. 2023;576:112000. 10.1016/j.mce.2023.112000. 37460007

[284] Guo H, Yang Y, Qiao Y, He J, Yao W, Zheng W. Heat stress affects fetal brain and intestinal function associated with the alterations of placental barrier in late pregnant mouse. Ecotoxicol Environ Saf. 2021;227:112916. 10.1016/j.ecoenv.2021.112916. 34695613

[285] Zhou JY, Huang DG, Zhu M, Gao CQ, Yan HC, Li XG, et al. Wnt/β-catenin-mediated heat exposure inhibits intestinal epithelial cell proliferation and stem cell expansion through endoplasmic reticulum stress. J Cell Physiol. 2020;235(7–8):5613–27. 10.1002/jcp.29492. 31960439

[286] Saada M, Sanchez-Jimenez E, Roguin A. Risk of ionizing radiation in pregnancy: just a myth or a real concern? Europace. 2023;25(2):270–6. 10.1093/europace/euac158. PMC10103573 36125209

[287] Streffer C, Hande MP. Effects of ionizing radiation exposure in offspring and next generations. Int J Radiat Biol. 2024;100(9):1237–9. 10.1080/09553002.2024.2384834. 39136525

[288] Dartnell LR. Ionizing radiation and life. Astrobiology. 2011;11(6):551–82. 10.1089/ast.2010.0528. 21774684

[289] Hurem S, Martín LM, Lindeman L, Brede DA, Salbu B, Lyche JL, et al. Parental exposure to gamma radiation causes progressively altered transcriptomes linked to adverse effects in zebrafish offspring. Environ Pollut. 2018;234:855–63. 10.1016/j.envpol.2017.12.023. 29248853

[290] Tharmalingam S, Sreetharan S, Brooks AL, Boreham DR. Re-evaluation of the linear no-threshold (LNT) model using new paradigms and modern molecular studies. Chem Biol Interact. 2019;301:54–67. 10.1016/j.cbi.2018.11.013. 30763548

[291] Kanter DJ, O’Brien MB, Shi XH, Chu T, Mishima T, Beriwal S, et al. The impact of ionizing radiation on placental trophoblasts. Placenta. 2014;35(2):85–91. 10.1016/j.placenta.2013.12.011. PMC3954710 24418702

[292] Chen S, Wang Q, Han B, Wu J, Liu DK, Zou JD, et al. Effects of leptin-modified human placenta-derived mesenchymal stem cells on angiogenic potential and peripheral inflammation of human umbilical vein endothelial cells (HUVECs) after X-ray radiation. J Zhejiang Univ Sci B. 2020;21(4):327–40. 10.1631/jzus.B1900598. PMC7183445 32253842

[293] Hourtovenko C, Sreetharan S, Tharmalingam S, Tai TC. Impact of ionizing radiation exposure on placental function and implications for fetal programming. Int J Mol Sci. 2024;25(18). 10.3390/ijms25189862. PMC11432287 39337351

[294] Kamstra JH, Hurem S, Martin LM, Lindeman LC, Legler J, Oughton D, et al. Ionizing radiation induces transgenerational effects of DNA methylation in zebrafish. Sci Rep. 2018;8(1):15373. 10.1038/s41598-018-33817-w. PMC6193964 30337673

[295] Kong EY, Cheng SH, Yu KN. Zebrafish as an in vivo model to assess epigenetic effects of ionizing radiation. Int J Mol Sci. 2016;17(12). 10.3390/ijms17122108. PMC5187908 27983682

[296] Wang S, Meyer DH, Schumacher B. Inheritance of paternal DNA damage by histone-mediated repair restriction. Nature. 2023;613(7943):365–74. 10.1038/s41586-022-05544-w. PMC9834056 36544019

[297] Sholts SB, Korkalainen M, Simanainen U, Miettinen HM, Håkansson H, Viluksela M. In utero/lactational and adult exposures to 2,3,7,8-tetrachlorodibenzo-p-dioxin (TCDD) show differential effects on craniofacial development and growth in rats. Toxicology. 2015;337:30–8. 10.1016/j.tox.2015.08.010. 26320568

[298] Yoshioka W, Tohyama C. Mechanisms of developmental toxicity of dioxins and related compounds. Int J Mol Sci. 2019;20(3). 10.3390/ijms20030617. PMC6387164 30708991

[299] Cintrón-Rivera LG, Burns N, Patel R, Plavicki JS. Exposure to the aryl hydrocarbon receptor agonist dioxin disrupts formation of the muscle, nerves, and vasculature in the developing jaw. Environ Pollut. 2023;337:122499. 10.1016/j.envpol.2023.122499. 37660771

[300] Wang XM, Qin CM, Li D, Xu XR, Pan XJ, Xue H. Comprehensive three-dimensional microCT and signaling analysis reveal the teratogenic effect of 2,3,7,8-tetrachlorodibenzo-p-dioxin on craniofacial bone development in mice. Ecotoxicol Environ Saf. 2025;290:117743. 10.1016/j.ecoenv.2025.117743. 39823675

[301] Tao Y, Liu X, Cui L, Liu X, Chen Y, He Z, et al. Oct4 plays a role in 2, 3, 7, 8 - tetrachlorobenzo-p-dioxin (TCDD) inducing cleft palate and inhibiting mesenchymal proliferation. Toxicol. 2020;438:152444. 10.1016/j.tox.2020.152444. 32283119

[302] Coker SJ, Smith-Díaz CC, Dyson RM, Vissers MCM, Berry MJ. The Epigenetic role of vitamin C in Neurodevelopment. Int J Mol Sci. 2022;23(3). 10.3390/ijms23031208. PMC8836017 35163133

[303] Caton JS, Crouse MS, Dahlen CR, Ward AK, Diniz WJS, Hammer CJ, et al. International Symposium on Ruminant Physiology: Maternal nutrient supply-Impacts on physiological and whole-animal outcomes in offspring. J Dairy Sci. 2025;108(7):7696–709. 10.3168/jds.2024-25788. 39710263

[304] Beames TG, Lipinski RJ. Gene-environment interactions: aligning birth defects research with complex etiology. Development. 2020;147(21). 10.1242/dev.191064. PMC7375468 32680836

[305] Shi M, Christensen K, Weinberg CR, Romitti P, Bathum L, Lozada A, et al. Orofacial cleft risk is increased with maternal smoking and specific detoxification-gene variants. Am J Hum Genet. 2007;80(1):76–90. 10.1086/510518. PMC1785306 17160896

[306] Jugessur A, Wilcox AJ, Murray JC, Gjessing HK, Nguyen TT, Nilsen RM, et al. Assessing the impact of nicotine dependence genes on the risk of facial clefts: An example of the use of national registry and biobank data. Nor Epidemiol. 2012;21(2):241–50. 10.5324/nje.v21i2.1500. PMC4594948 26451072

[307] Machado RA, Moreira HSB, de Aquino SN, Martelli-Junior H, de Almeida Reis SR, Persuhn DC, et al. Interactions between RAD51 rs1801321 and maternal cigarette smoking as risk factor for nonsyndromic cleft lip with or without cleft palate. Am J Med Genet A. 2016;170a(2):536–539. 10.1002/ajmg.a.37281. 26507587

[308] Romitti PA, Lidral AC, Munger RG, Daack-Hirsch S, Burns TL, Murray JC. Candidate genes for nonsyndromic cleft lip and palate and maternal cigarette smoking and alcohol consumption: evaluation of genotype-environment interactions from a population-based case-control study of orofacial clefts. Teratology. 1999;59(1):39–50. 10.1002/(sici)1096-9926(199901)59:1<39::Aid-tera9>3.0.Co;2-7. 9988882

[309] Alvizi L, Nani D, Brito LA, Kobayashi GS, Passos-Bueno MR, Mayor R. Neural crest E-cadherin loss drives cleft lip/palate by epigenetic modulation via pro-inflammatory gene-environment interaction. Nat Commun. 2023;14(1):2868. 10.1038/s41467-023-38526-1. PMC10209087 37225711

[310] Yin B, Xu YC, Lu Y. Oxidative stress-mediated apoptosis via the SLC23A2-ascorbic acid interaction contributes to cleft lip development. Front Pediatr. 2025;13:1632778. 10.3389/fped.2025.1632778. PMC12527864 41113559

[311] Hong M, Krauss RS. Cdon mutation and fetal ethanol exposure synergize to produce midline signaling defects and holoprosencephaly spectrum disorders in mice. PLoS Genet. 2012;8(10):e1002999. 10.1371/journal.pgen.1002999. PMC3469434 23071453

[312] Gaynor SM, Joseph T, Bai X, Zou Y, Boutkov B, Maxwell EK, et al. Yield of genetic association signals from genomes, exomes and imputation in the UK Biobank. Nat Genet. 2024;56(11):2345–51. 10.1038/s41588-024-01930-4. PMC11549045 39322778

[313] Beers BJ, Similuk MN, Ghosh R, Seifert BA, Jamal L, Kamen M, et al. Chromosomal microarray analysis supplements exome sequencing to diagnose children with suspected inborn errors of immunity. Front Immunol. 2023;14:1172004. 10.3389/fimmu.2023.1172004. PMC10196392 37215141

[314] Topa A, Rohlin A, Fehr A, Lovmar L, Stenman G, Tarnow P, et al. The value of genome-wide analysis in craniosynostosis. Front Genet. 2023;14:1322462. 10.3389/fgene.2023.1322462. PMC10839781 38318288

[315] Slavec L, Geršak K, Eberlinc A, Hovnik T, Lovrečić L, Mlinarič-Raščan I, et al. A comprehensive genetic analysis of slovenian families with multiple cases of orofacial clefts reveals novel variants in the genes IRF6, GRHL3, and TBX22. Int J Mol Sci. 2023;24(5). 10.3390/ijms24054262. PMC10002089 36901693

[316] Yu Z, Feng C, Li T, Duan H, Zhang H. Advances in genetic mechanisms and precision medicine research for cleft lip and palate. Gene. 2026;988:150045. 10.1016/j.gene.2026.150045. 41687982

[317] Dauwerse JG, Dixon J, Seland S, Ruivenkamp CA, van Haeringen A, Hoefsloot LH, et al. Mutations in genes encoding subunits of RNA polymerases I and III cause Treacher Collins syndrome. Nat Genet. 2011;43(1):20–2. 10.1038/ng.724. 21131976

[318] Kaelin Agten A, Xia J, Servante JA, Thornton JG, Jones NW. Routine ultrasound for fetal assessment before 24 weeks' gestation. Cochrane Database Syst Rev. 2021;8(8):Cd014698. 10.1002/14651858.Cd014698. PMC8407184 34438475

[319] Clark A, Flouri D, Mufti N, James J, Clements E, Aughwane R, et al. Developments in functional imaging of the placenta. Br J Radiol. 2023;96(1147):20211010. 10.1259/bjr.20211010. PMC10321248 35234516

[320] Chen J, Kanekar S. Imaging of Congenital Craniofacial Anomalies and Syndromes. Clin Perinatol. 2022;49(3):771–90. 10.1016/j.clp.2022.04.005. 36113934

[321] Liao M, Wang L, Shang N, Hu X, He B, Liu X, et al. Ultrasound measurements of fetal facial profile markers and their associations with congenital malformations during early pregnancy. BMC Pregnancy Childbirth. 2023;23(1):772. 10.1186/s12884-023-06067-6. PMC10625258 37925422

[322] Kageyama S, Sato T, Iwama N, Hamada H, Saito M, Takase K. MR imaging of umbilical cord variations, abnormalities, and associated placental findings. Magn Reson Med Sci. 2025;24(3):366–86. 10.2463/mrms.rev.2025-0036. PMC12354972 40738692

[323] Carocha A, Vicente M, Bernardeco J, Rijo C, Cohen Á, Cruz J. 2nd trimester ultrasound (anomaly). Best Pract Res Clin Obstet Gynaecol. 2025;101:102628. 10.1016/j.bpobgyn.2025.102628. 40540853

[324] Shreeve N, Sproule C, Choy KW, Dong Z, Gajewska-Knapik K, Kilby MD, et al. Incremental yield of whole-genome sequencing over chromosomal microarray analysis and exome sequencing for congenital anomalies in prenatal period and infancy: systematic review and meta-analysis. Ultrasound Obstet Gynecol. 2024;63(1):15–23. 10.1002/uog.27491. 37725747

[325] Hu P, Zhang Q, Cheng Q, Luo C, Zhang C, Zhou R, et al. Whole genome sequencing vs chromosomal microarray analysis in prenatal diagnosis. Am J Obstet Gynecol. 2023;229(3):302.e1-302.e18. 10.1016/j.ajog.2023.03.005. 36907537

[326] Agache I, Akdis CA. Precision medicine and phenotypes, endotypes, genotypes, regiotypes, and theratypes of allergic diseases. J Clin Invest. 2019;129(4):1493–503. 10.1172/jci124611. PMC6436902 30855278

[327] Maron JL, Kingsmore S, Gelb BD, Vockley J, Wigby K, Bragg J, et al. Rapid Whole-Genomic Sequencing and a Targeted Neonatal Gene Panel in Infants With a Suspected Genetic Disorder. JAMA. 2023;330(2):161–9. 10.1001/jama.2023.9350. PMC10336625 37432431

[328] Li C, Huang W, Gan Z, Ling Y, Qiu L, Xiao Y, et al. Integrating imaging and genomics in prenatal Treacher Collins syndrome: evidence for practice and policy. Orphanet J Rare Dis. 2026;21(1). 10.1186/s13023-025-04094-4. PMC13001374 41857598

[329] Li J, Zhan Z, Zhang X, Wu B, Liu W. Comprehensive genotype-phenotype correlation analysis in 11 509 neonates carrying common deafness-associated pathogenic variants. J Med Genet. 2026;63(6):368–75. 10.1136/jmg-2025-111135. PMC13217119 41876127

[330] Than NG, Papp Z. Ethical issues in genetic counseling. Best Pract Res Clin Obstet Gynaecol. 2017;43:32–49. 10.1016/j.bpobgyn.2017.01.005. 28259715

[331] Maarse W, Rozendaal AM, Pajkrt E, Vermeij-Keers C, Mink van der Molen AB, van den Boogaard MJ. A systematic review of associated structural and chromosomal defects in oral clefts: when is prenatal genetic analysis indicated? J Med Genet. 2012;49(8):490–8. 10.1136/jmedgenet-2012-101013. 22889852

[332] Wilkes C, Graetz M, Downie L, Bethune M, Chong D. Prenatal diagnosis of cleft lip and/or palate: What do we tell prospective parents? Prenat Diagn. 2023;43(10):1310–9. 10.1002/pd.6418. 37552068

[333] Kingdom JC, Audette MC, Hobson SR, Windrim RC, Morgen E. A placenta clinic approach to the diagnosis and management of fetal growth restriction. Am J Obstet Gynecol. 2018;218(2s):S803-s817. 10.1016/j.ajog.2017.11.575. 29254754

[334] Ashwal E, Ferreira F, Mei-Dan E, Aviram A, Sherman C, Zaltz A, et al. The accuracy of fetoplacental Doppler in distinguishing between growth restricted and constitutionally small fetuses. Placenta. 2022;120:40–8. 10.1016/j.placenta.2022.02.007. 35189547

[335] Spencer R, Maksym K, Hecher K, Maršál K, Figueras F, Ambler G, et al. Maternal PlGF and umbilical Dopplers predict pregnancy outcomes at diagnosis of early-onset fetal growth restriction. J Clin Invest. 2023;133(18). 10.1172/jci169199. PMC10503803 37712421

[336] Mitchell LE. Maternal effect genes: Update and review of evidence for a link with birth defects. HGG Adv. 2022;3(1):100067. 10.1016/j.xhgg.2021.100067. PMC8756509 35047854

[337] Sharif M, Anvar Z, Chakchouk I, El-Dessouky SH, Zemet R, Kao EC, et al. Loss of the Maternal Effect Gene NLRP2 Impairs Embryonic and Extra-Embryonic Development, Revealing a Novel Genetic Cause of Congenital Anomalies†. Biol Reprod. 2025. 10.1093/biolre/ioaf290. PMC13079454 41454780

[338] Mennitti LV, Oliveira JL, Morais CA, Estadella D, Oyama LM, Oller do Nascimento CM, et al. Type of fatty acids in maternal diets during pregnancy and/or lactation and metabolic consequences of the offspring. J Nutr Biochem. 2015;26(2):99–111. 10.1016/j.jnutbio.2014.10.001. 25459884

[339] Belkacemi L, Nelson DM, Desai M, Ross MG. Maternal undernutrition influences placental-fetal development. Biol Reprod. 2010;83(3):325–31. 10.1095/biolreprod.110.084517. 20445129

[340] Hojeij B, Rousian M, Sinclair KD, Dinnyes A, Steegers-Theunissen RPM, Schoenmakers S. Periconceptional biomarkers for maternal obesity: a systematic review. Rev Endocr Metab Disord. 2023;24(2):139–75. 10.1007/s11154-022-09762-5. PMC10023635 36520252

[341] Capra ME, Bellani A, Berzieri M, Fradusco A, Esposito S, Biasucci G. Maternal nutrition during pregnancy and fetal outcome, short- and long-term health effects: a narrative review. Nutrients. 2026;18(9). 10.3390/nu18091375. PMC13165033 42123976

[342] Favara G, Maugeri A, Magnano San Lio R, Barchitta M, Agodi A. Exploring gene-diet interactions for mother-child health: a systematic review of epidemiological studies. Nutrients. 2024;16(7). 10.3390/nu16070994. PMC11013682 38613027

[343] Blanco R, Colombo A, Suazo J. Maternal obesity is a risk factor for orofacial clefts: a meta-analysis. Br J Oral Maxillofac Surg. 2015;53(8):699–704. 10.1016/j.bjoms.2015.05.017. 26073906

[344] Seelan RS, Greene RM, Pisano MM. Role of lncRNAs and circRNAs in Orofacial Clefts. Microrna. 2023;12(3):171–6. 10.2174/2211536612666230524153442. 38009000

[345] Hong L, Sun H, Amendt BA. MicroRNA function in craniofacial bone formation, regeneration and repair. Bone. 2021;144:115789. 10.1016/j.bone.2020.115789. PMC7869528 33309989

[346] Benizri S, Gissot A, Martin A, Vialet B, Grinstaff MW, Barthélémy P. Bioconjugated Oligonucleotides: Recent Developments and Therapeutic Applications. Bioconjug Chem. 2019;30(2):366–83. 10.1021/acs.bioconjchem.8b00761. PMC6766081 30608140

[347] Garland MA, Sun B, Zhang S, Reynolds K, Ji Y, Zhou CJ. Role of epigenetics and miRNAs in orofacial clefts. Birth Defects Res. 2020;112(19):1635–59. 10.1002/bdr2.1802. PMC7924905 32926553

[348] Hazrati P, Alanazi A, Alrmali AE, Galindo-Fernandez P, Kassem H, Kaigler D. Clinical stem cell therapy in oral and craniofacial bone regeneration: a systematic review and meta-analysis. Front Bioeng Biotechnol. 2026;14:1677400. 10.3389/fbioe.2026.1677400. PMC12876186 41658988

[349] Khalaf AT, Wei Y, Wan J, Zhu J, Peng Y, Abdul Kadir SY, et al. Bone tissue engineering through 3D bioprinting of bioceramic scaffolds: a review and update. Life (Basel). 2022. 10.3390/life12060903. PMC9225502 35743934

[350] Dziedzic A, Kubina R, Skonieczna M, Madej M, Fiegler-Rudol J, Abid M, et al. CRISPR genome editing in personalized therapy for oral and maxillofacial diseases: a scoping review. Biomedicines. 2025;13(11). 10.3390/biomedicines13112745. PMC12650159 41301838

[351] Fitriasari S, Trainor PA. Gene-environment interactions in the pathogenesis of common craniofacial anomalies. Curr Top Dev Biol. 2023;152:139–68. 10.1016/bs.ctdb.2022.10.005. 36707210

[352] Feng J, Janečková E, Guo T, Ziaei H, Zhang M, Geng JJ, et al. High-resolution spatial transcriptomics and cell lineage analysis reveal spatiotemporal cell fate determination during craniofacial development. Nat Commun. 2025;16(1):4396. 10.1038/s41467-025-59206-2. PMC12069723 40355462

[353] Zhang W, Venkataraghavan S, Hetmanski JB, Leslie EJ, Marazita ML, Feingold E, et al. Detecting Gene-Environment Interaction for Maternal Exposures Using Case-Parent Trios Ascertained Through a Case With Non-Syndromic Orofacial Cleft. Front Cell Dev Biol. 2021;9:621018. 10.3389/fcell.2021.621018. PMC8085423 33937227

[354] Shekhawat DS, Kulkarni U, Sharma C, Singh P, Singh K. Assessing the Ethical Considerations in the Era of Advanced Prenatal Testing and Genetic Counselling - Clinicians’ Perspective. Indian J Community Med. 2026;51(2):223–8. 10.4103/ijcm.ijcm_836_24. PMC13160193 42125582

[355] Bonaccorso A, Nespoli A, Ferioli E, Picozzi M. Ethical challenges and moral conflicts in periviability prenatal counseling: a scoping review of healthcare professionals’ perspectives. BMC Med Ethics. 2026. 10.1186/s12910-026-01464-w. PMC13366718 42121231

[356] Qi Q, Jiang Y, Zhou X, Lü Y, Xiao R, Bai J, et al. Whole-genome sequencing analysis in fetal structural anomalies: novel phenotype-genotype discoveries. Ultrasound Obstet Gynecol. 2024;63(5):664–71. 10.1002/uog.27517. 37842862

[357] Yang C, Ding L, Dong Y, Wang Y, Cao S, Yuan Z, et al. Advancements in Pathogenic Genes and Biomarkers for Non-syndromic Cleft Lip With or Without Cleft Palate Via Multiomics. Int Dent J. 2026;76(1):109282. 10.1016/j.identj.2025.109282. PMC12666830 41265165

[358] Piña JO, Raju R, Stipano E, Myo AC, Wang Z, Ono M, et al. Single cell spatial transcriptomics links Wnt signaling disruption to extracellular matrix development in a cleft palate model. Sci Rep. 2025;15(1):29639. 10.1038/s41598-025-14807-1. PMC12350917 40804270

[359] Khouri-Farah N, Manchel A, Wentworth Winchester E, Schilder BM, Robinson K, Curtis S W, et al. Gene expression dynamics of human and mouse craniofacial development at the single-cell level. Nat Commun. 2026;17(1). 10.1038/s41467-026-70232-6. PMC13102946 41803119

[360] Cianflone A, Coppola L, Primo P, Maisto G, Mastrodonato F, Di Palma MA, et al. Point of care testing, rapid next generation sequencing and artificial intelligence in pediatric and neonatal healthcare: a narrative review. Pharmaceuticals (Basel). 2025;18(11). 10.3390/ph18111721. PMC12655080 41304965

[361] Xu W, Cai H, Zhang Q, Wang Z, Yang J, Wu X, et al. U-FISH: a fluorescent spot detector for imaging-based spatial-omics analysis and AI-assisted FISH diagnosis. Genome Biol. 2025;26(1):261. 10.1186/s13059-025-03736-x. PMC12400573 40890868

[362] Rego S, Grove ME, Cho MK, Ormond KE. Informed consent in the genomics era. Cold Spring Harb Perspect Med. 2020. 10.1101/cshperspect.a036582. PMC7397836 31570382

[363] Ormond KE, Borensztein MJ, Hallquist MLG, Buchanan AH, Faucett WA, Peay HL, et al. Defining the Critical Components of Informed Consent for Genetic Testing. J Pers Med. 2021;11(12). 10.3390/jpm11121304. PMC8706495 34945775

[364] Hallquist MLG, Borensztein MJ, Coughlin CR 2nd, Buchanan H, Andrew Faucett W, Peay HL, et al. Defining critical educational components of informed consent for genetic testing: views of US-based genetic counselors and medical geneticists. Eur J Hum Genet. 2023;31(10):1165–74. 10.1038/s41431-023-01401-0. PMC10545703 37308598

